# Supplementary material for: Sesquiterpenoids from the Mangrove-Derived Aspergillus ustus 094102
Source: Mar Drugs. 2022 Jun 22;20(7):408. doi: 10.3390/md20070408 (PMC9322191; doi:10.3390/md20070408)
Supplement: Supplementary file 1 [file marinedrugs-20-00408-s001.zip › marinedrugs-1760413 SI .pdf]

# Supporting Information

# Sesquiterpenoids from the Mangrove-derived *Aspergillus ustus* 094102

**Pengyan Gui [1,Δ], Jie Fan [1,3,Δ], Tonghan Zhu [1,4], Peng Fu [1,2], Kui Hong [5,*] and Weiming Zhu [1,2,*]**

[1] Key Laboratory of Marine Drugs, Ministry of Education of China, School of Medicine and Pharmacy, Ocean University of China, Qingdao 266003, People's Republic of China; 11180811003@stu.ouc.edu.cn (P.G.)

[2] Laboratory for Marine Drugs and Bioproducts of Qingdao National Laboratory for Marine Science and Technology; Qingdao 266003, China; fupeng@ouc.edu.cn (P.F.)

[3] Qingdao Marine Technical College, Qingdao 266590, China; fj900622@163.com (J.F.)

[4] College of Civil Engineering and Architecture, Shandong University of Science and Technology, Qingdao 266590, China; sdueduzth@126.com (T.Z.)

[5] Key Laboratory of Combinatorial Biosynthesis and Drug Discovery, Ministry of Education and School of Pharmaceutical Sciences, Wuhan University, Wuhan 430071, China; kuihong31@whu.edu.cn (K.H.)

[Δ] Authors contributed equally to this paper.

* Correspondence: weimingzhu@ouc.edu.cn (W.Z.); Tel./Fax: +86-532-82031268 (W.Z.); kuihong31@whu.edu.cn (K.H.).

## List of Supporting Information


**Bioassay Protocols** ........ S5
**Figure S1**. The HRESIMS spectrum of compound **1** ........ S6
**Figure S2**. The IR spectrum of compound **1** ........ S7
**Figure S3**. The $^{1}$H-NMR spectrum of compound **1** in DMSO-$d_6$ ........ S7
**Figure S4**. The $^{13}$C-NMR and DEPT spectrum of compound **1** in DMSO-$d_6$ ........ S8
**Figure S5**. The DEPT spectrum of compound **1** in DMSO-$d_6$ ........ S8
**Figure S6**. The $^{1}$H-$^{1}$H COSY spectrum of compound **1** in DMSO-$d_6$ ........ S9
**Figure S7**. The HSQC spectrum of compound **1** in DMSO-$d_6$ ........ S9
**Figure S8**. The HMBC spectrum of compound **1** in DMSO-$d_6$ ........ S10
**Figure S9**. The NOESY spectrum of compound **1** in DMSO-$d_6$ ........ S10
**Figure S10**. The ECD curve of compound **1** ........ S11
**Figure S11**. The HRESIMS spectrum of compound **2** ........ S11
**Figure S12**. The IR spectrum of compound **2** ........ S12
**Figure S13**. The $^{1}$H-NMR spectrum of compound **2** in DMSO-$d_6$ ........ S12
**Figure S14**. The $^{13}$C-NMR spectrum of compound **2** in DMSO-$d_6$ ........ S13
**Figure S15**. The DEPT spectrum of compound **2** in DMSO-$d_6$ ........ S13
**Figure S16**. The $^{1}$H-$^{1}$H COSY spectrum of compound **2** in DMSO-$d_6$ ........ S14
**Figure S17**. The HSQC spectrum of compound **2** in DMSO-$d_6$ ........ S14
**Figure S18**. The HMBC spectrum of compound **2** in DMSO-$d_6$ ........ S15
**Figure S19**. The NOESY spectrum of compound **2** in DMSO-$d_6$ ........ S16
**Figure S20**. ECD curve for the complex of **2** with $Mo_2(OAc)_4$ subtracted from the inherent ECD ........ S16
**Figure S21**.The HRESIMS spectrum of compound **3** ........ S17
**Figure S22**. The IR spectrum of compound **3** ........ S17
**Figure S23**. The $^{1}$H-NMR spectrum of compound **3** in DMSO-$d_6$ ........ S18
**Figure S24**. The $^{13}$C-NMR spectrum of compound **3** in DMSO-$d_6$ ........ S18
**Figure S25**. The DEPT spectrum of compound **3** in DMSO-$d_6$ ........ S19
**Figure S26**. The $^{1}$H-$^{1}$H COSY spectrum of compound **3** in DMSO-$d_6$ ........ S19
**Figure S27**. The HSQC spectrum of compound **3** in DMSO-$d_6$ ........ S20
**Figure S28**. The HMBC spectrum of compound **3** in DMSO-$d_6$ ........ S20
**Figure S29**. NOE difference spectrum of compound **3** in DMSO-$d_6$ ........ S21
**Figure S30**. The ECD curve of compound **3** ........ S21
**Figure S31**. The ECD curves of **1**–**3** and **7** ........ S22
**Figure S32**. The HRESIMS spectrum of compound **4** ........ S22
**Figure S33**. The IR spectrum of compound **4** ........ S23
**Figure S34**. The $^{1}$H-NMR spectrum of compound **4** in DMSO-$d_6$ ........ S23
**Figure S35**. The $^{13}$C-NMR spectrum of compound **4** in DMSO-$d_6$ ........ S24
**Figure S36**. The DEPT spectrum of compound **4** in DMSO-$d_6$ ........ S24
**Figure S37**. The $^{1}$H-$^{1}$H COSY spectrum of compound **4** in DMSO-$d_6$ ........ S25
**Figure S38**. The HSQC spectrum of compound **4** in DMSO-$d_6$ ........ S25
**Figure S39**. The HMBC spectrum of compound **4** in DMSO-$d_6$ ........ S26
**Figure S40**. The NOESY spectrum of compound **4** in DMSO-$d_6$ ........ S26
**Figure S41**. The ECD curve of compound **4** ........ S27
**Figure S42**. The ESIMS spectrum of compound **5** ........ S28

**Figure S43**. The $^{1}$H-NMR spectrum of compound **5** in DMSO-$d_6$ ........ S28
**Figure S44**. The $^{13}$C-NMR spectrum of compound **5** in DMSO-$d_6$ ........ S29
**Figure S45**. The $^{13}$H-NMR spectrum of compound **5a** in DMSO-$d_6$ ........ S29
**Figure S46.** The $^{13}$C-NMR spectrum of compound **5a** in DMSO-$d_6$ ........ S30
**Figure S47**. The $^{1}$H-$^{1}$H COSY spectrum of compound **5a** in DMSO-$d_6$ ........ S30
**Figure S48**. The HSQC spectrum of compound **5a** in DMSO-$d_6$ ........ S31
**Figure S49**. The HMBC spectrum of compound **5a** in DMSO-$d_6$ ........ S32
**Figure S50.** The $^{1}$H-NMR spectrum of compound **5b** in DMSO-$d_6$ ........ S32
**Figure S51.** The $^{13}$C-NMR spectrum of compound **5b** in DMSO-$d_6$ ........ S33
**Figure S52.** The $^{1}$H-NMR spectrum of compound **5c** in DMSO-$d_6$ ........ S33
**Figure S53.** The $^{13}$C-NMR spectrum of compound **5c** in DMSO-$d_6$ ........ S34
**Figure S54**. The $^{1}$H-$^{1}$H COSY spectrum of compound **5c** in DMSO-$d_6$ ........ S34
**Figure S55**. The HSQC spectrum of compound **5c** in DMSO-$d_6$ ........ S35
**Figure S56**. The HMBC spectrum of compound **5c** in DMSO-$d_6$ ........ S35
**Figure S57**. The $^{1}$H-NMR spectrum of compound **5d** in DMSO-$d_6$ ........ S36
**Figure S58.** The $^{13}$C-NMR spectrum of compound **5d** in DMSO-$d_6$ ........ S36
**Figure S59.** The $^{1H}$C-NMR difference of compound **5a**, **5b**, **5c** and **5d** between $\delta_H$ 3.0-6.5 ........ S37
**Figure S60**. $^{13}$C-NMR difference of **5a**, **5b**, **5c** and **5d** between methyl carbon (C-8') ........ S37
**Figure S61**. The ECD curve of compound **5a** ........ S38
**Figure S62**. The ECD curve of compound **5b** ........ S38
**Figure S63**. The ECD curve of compound **5c** ........ S39
**Figure S64**. The ECD curve of compound **5d** ........ S39
**Figure S65**. The ESIMS spectrum of compound **5e** ........ S40
**Figure S66.** The $^{1}$H-NMR spectrum of compound **5e** in DMSO-$d_6$ ........ S40
**Figure S67.** The $^{13}$C-NMR spectrum of compound **5e** in DMSO-$d_6$ ........ S41
**Figure S68**. The $^{1}$H-$^{1}$H COSY spectrum of compound **5e** in DMSO-$d_6$ ........ S41
**Figure S69**. The HSQC spectrum of compound **5e** in DMSO-$d_6$ ........ S42
**Figure S70**. The HMBC spectrum of compound **5e** in DMSO-$d_6$ ........ S42
**Figure S71**. The NOESY spectrum of compound **5e** in DMSO-$d_6$ ........ S43
**Figure S72**. The ECD curve of compound **5e** ........ S44
**Figure S73**. ECD curve for the complex of **5c** with $Mo_2(OAc)_4$ subtracted from the inherent ECD ........ S44
**Figure S74**. ECD curve for the complex of **5d** with $Mo_2(OAc)_4$ subtracted from the inherent ECD ........ S45
**Figure S75**. The $^{1}$H-NMR spectrum of compound **6** in DMSO-$d_6$ ........ S45
**Figure S76**. The $^{13}$C-NMR spectrum of compound **6** in DMSO-$d_6$ ........ S46
**Figure S77**. The ECD curve of compound **6** ........ S46
**Figure S78**. The ECD curves of **4**–**6** ........ S47
**Figure S79**. The $^{1}$H-NMR spectrum of compound **7** in DMSO-$d_6$ ........ S47
**Figure S80**. The $^{13}$C-NMR spectrum of compound **7** in DMSO-$d_6$ ........ S48
**Figure S81**. The NOESY spectrum of compound **7** in DMSO-$d_6$ ........ S48
**Figure S81**. The ECD curve of compound **7** ........ S49
**Figure S82**. The HPLC separation and purification profiles of **5a-5d** ........ S49
**Figure S83**. The HPLC profile of compound **1** ........ S50
**Figure S84**. The HPLC profile of compound **2** ........ S50
**Figure S85**. The HPLC profile of compound **3** ........ S50

**Figure S86**. The HPLC profile of compound **4**........................................................................................... S50
**Figure S87**. The HPLC profile of compound **5**........................................................................................... S51
**Figure S88**. The HPLC profile of compound **6**........................................................................................... S51
**Figure S89**. The HPLC profile of compound **7**........................................................................................... S51
**Figure S90**. The HPLC profiles of compound **7**, Ustusol B and their mixture........................................................ S51
**Figure S91**. The HPLC profile of compound **7** and ustusol B…………………………………………………..S51
**Table S1**. $^1$H and $^{13}$C NMR Data for Compound **7** and Ustusol B in DMSO-$d_6$.....……………………………………S52

**Bioassay Protocols**

**Antimicrobial Assay.** The antibiotic activities against P*seudomonas aeruginosa* ATCC10145, *Escherichia coli* ATCC11775, *Staphylococcus aureus* ATCC6538, Methicillin-resistant *Staphylococcus aureus subsp. aureus* ATCC 43300, *Candida albicans* ATCC 10231, *Candida glabrata* ATCC 2001, *Edwardsiella tarda* ATCC15947, *Bacillus cereus* ATCC14579, *Vibrio parahaemolyticus* ATCC17802, *Vibrio vulnificus* ATCC27562, *Acinetobacter baumannii* ATCC19606 and *Enterobacter aerogenes* ATCC13048 were evaluated by an agar dilution method. [S1] The tested strains were cultivated in LB/YPD agar plates at 28 °C. Compounds **1−7** and positive control (ciprofloxacin for bacteria and ketoconazole for fungi) were dissolved in methanol at a concentration of 0.1 mg/mL. A 10 $\mu$L quantity of test solution was absorbed by a paper disk (5 mm diameter) and placed on the assay plates. After 12 h incubation, zones of inhibition (mm in diameter) were recorded

**Cytotoxic Assays.** By the CCK-8 assay [S2, S3], compounds **1−7** were evaluated for cytotoxic activity against A549 (lung cancer cell line), HCT116 (colon carcinoma cell line), K562 (human erythroleukemic cell line), MCF-7 (human breast adenocarcinoma cell line), MKN-45 (human gastric cancer cell line), HL60 (human promyelocytic leukemia cell line), MDA-MB-231 (breast cancer cell line), DU145 (human prostate cancer cell line), A673 (rhabdomyoma cell line), A-375 (malignant melanoma of skin), Calu-3 (lung adenocarcinoma), HOS (human osteosarcoma cells), CAL-62 (Human thyroid cancer cells), HepG2 (human hepatoma cells), PA-1 (human ovarian teratoma cells), T98G (human glioma cells), 786-O (human renal clear cell adenocarcinoma cells), Huh-7 (human hepatoma cells), GBC-SD (human gallbladder cancer cells), PATU8988T (human pancreatic cancer cells), MG-63 (human osteosarcoma cells), FaDu (human pharyngeal squamous cell carcinoma cells), SF126 (human brain tumor cells), THP-1 (human monocytic leukemia), TE-1 (human esophageal cancer cells), 5637 (bladder cancer cells), HeLa (human cervical cancer cells), PC-9 (human lung cancer cells), Jurkat, Clone E6-1 (human T lymphocyte leukemia cells) and 293T (human embryonic kidney cells). In the CCK-8 assay, 30 human tumor cells and one human normal cell lines above were grown in DMEM supplemented in RPMI-1640 supplemented with 10% FBS under a humidified atmosphere of 5% $CO_2$ and 95% air at 37 °C. 90 $\mu$L of cell culture medium (Adherent cell viewed $5\times10^4$/mL and Suspension cell viewed $9\times10^4$/mL) per well, then cultured at 5% $CO_2$ and 37 °C for 24 hours. One concentration was set for each sample during preliminary screening and three multiple holes were set for each concentration. Eight concentration gradients were set for each sample for $IC_{50}$ determination and three multiple holes were set for each concentration. The 96 well plates were cultured at 5% $CO_2$ and 37 °C for 72 hours. The experiment was divided into blank group, control group and drug group. The old culture medium and drug solution of adherent cells was sucked out, then 100 $\mu$L of CCK-8 solution (diluted ten times with the basic medium) was added and the suspension cells was directly added 10 $\mu$L of CCK-8 stock solution. Culture at 37 °C with 5% $CO_2$ for 1–4 h (dark operation, real-time observation). The absorbance was measured at 450nm with an enzyme labeling instrument and the original data and results were recorded. Doxorubicin hydrochloride was used as the positive control with the $IC_{50}$ values of 0.0413 and 0.0961 $\mu$M, respectively.

**References:**


S1. Zaika, L.L. Spices and Herbs: their antimicrobial activity and its determination. *J. Food Safety* 1988, 9, 97-118.

S2. Tominaga H, Ishiyama M, Ohseto F, Sasamoto K, Hamamoto T, Suzuki T and Watanabe M. A water-soluble tetrazolium salt useful for colorimetric cell viability assay [J]. *Anal. Commun*. 1999, 36: 47-50.

S3. Wang, D.Y.; Wang, C.; Gui, P.Y.; Liu, H.S.; Khalaf, S.M.H.; Elsayed, E.A.; Wadaan, M.A.M.; Hozzein, W.N.; Zhu, W.M. Identification, bioactivity, and productivity of *Actinomycins* from the marine-derived *Streptomyces heliomycini*. *Front. Microbiol*. 2017, 8, 1147.

**Figure S1**. The HRESIMS spectrum of compound **1**

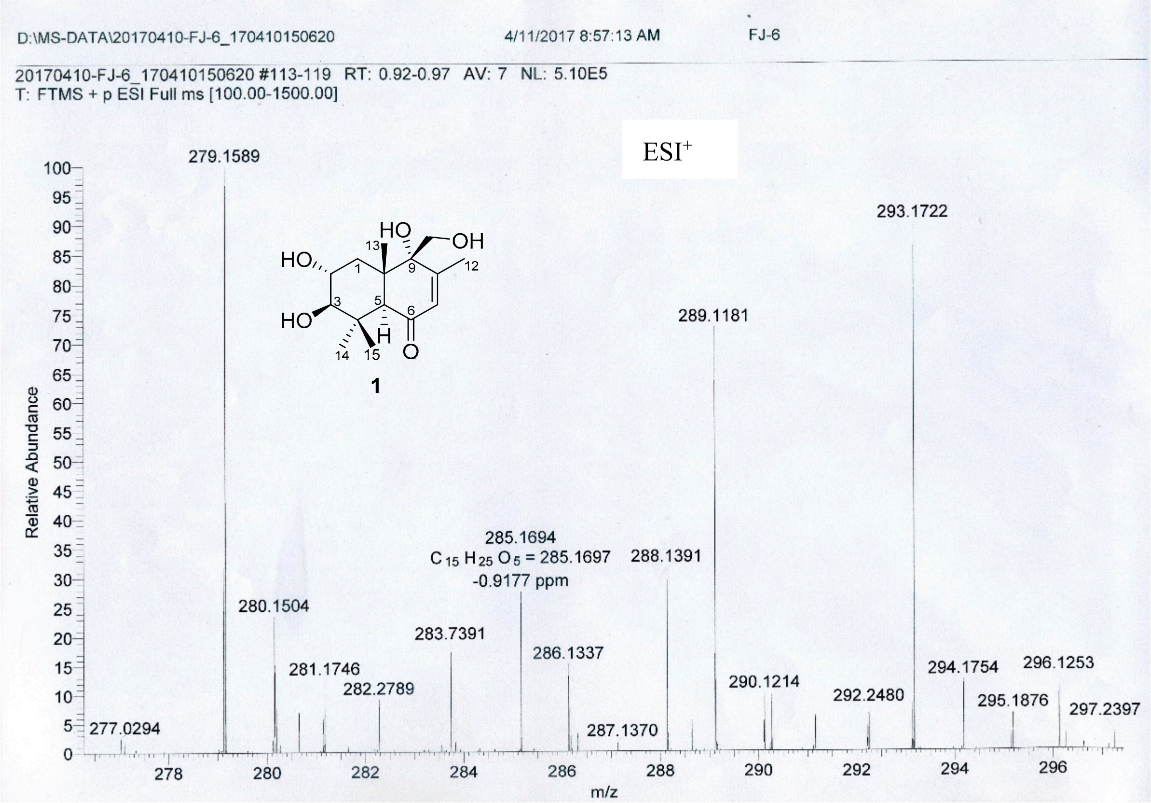


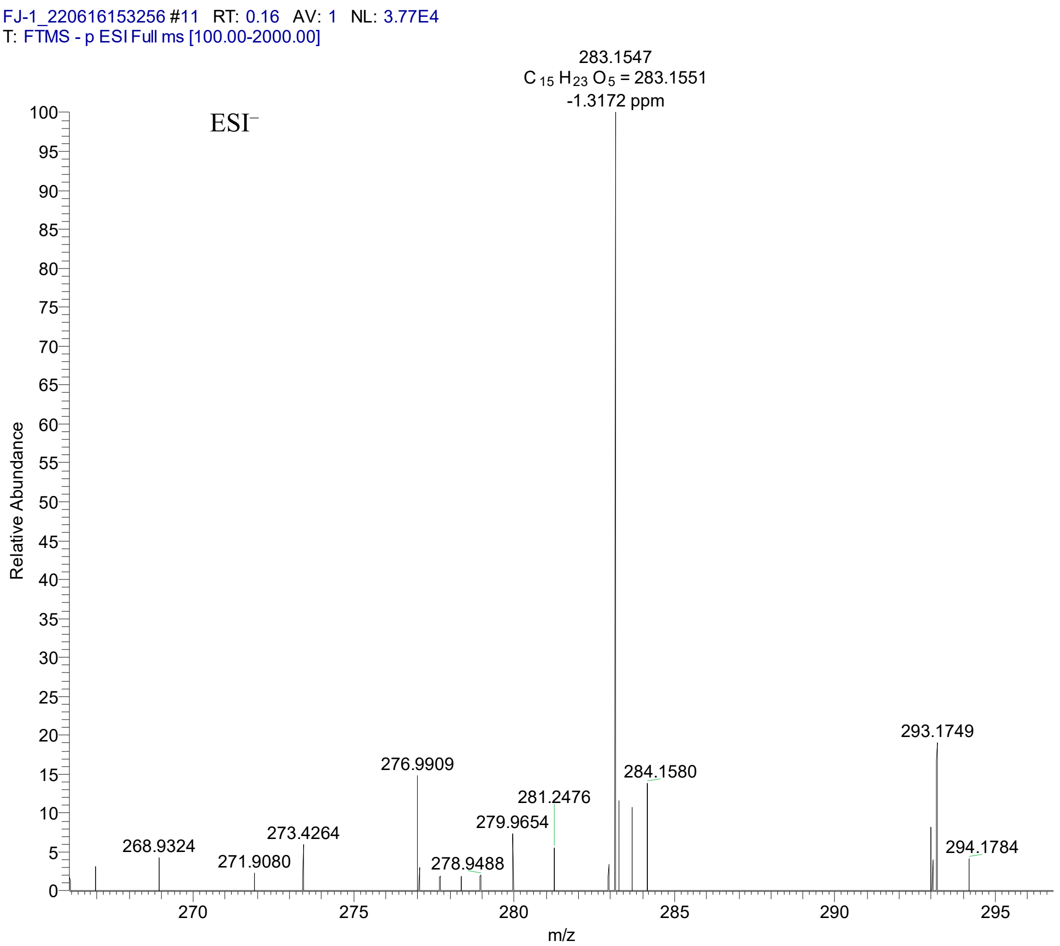

**Figure S2**. The IR spectrum of compound **1**

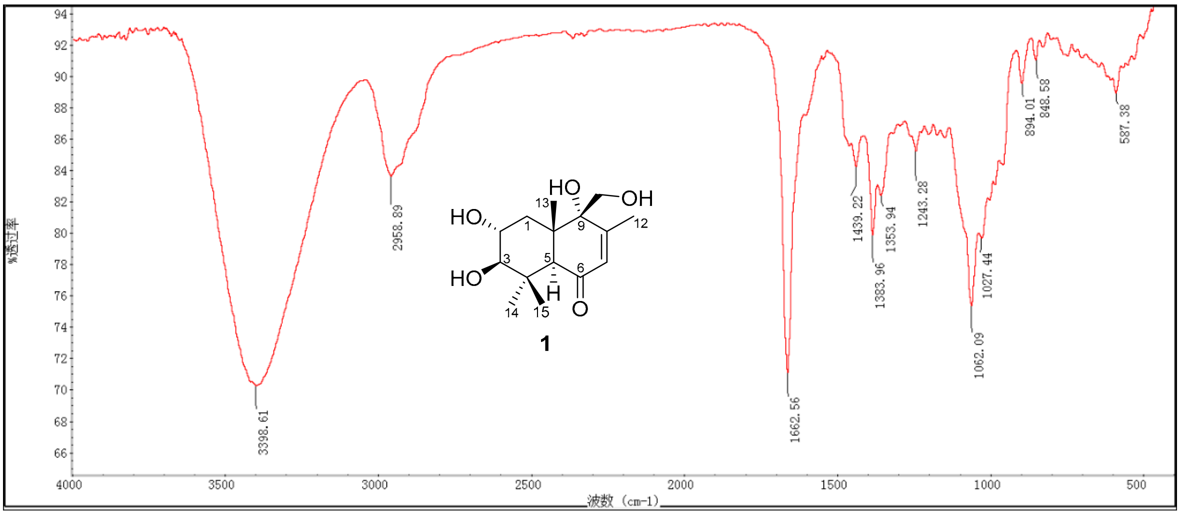


**Figure S3**. The $^{1}$H-NMR spectrum of compound **1** in DMSO-$d_6$

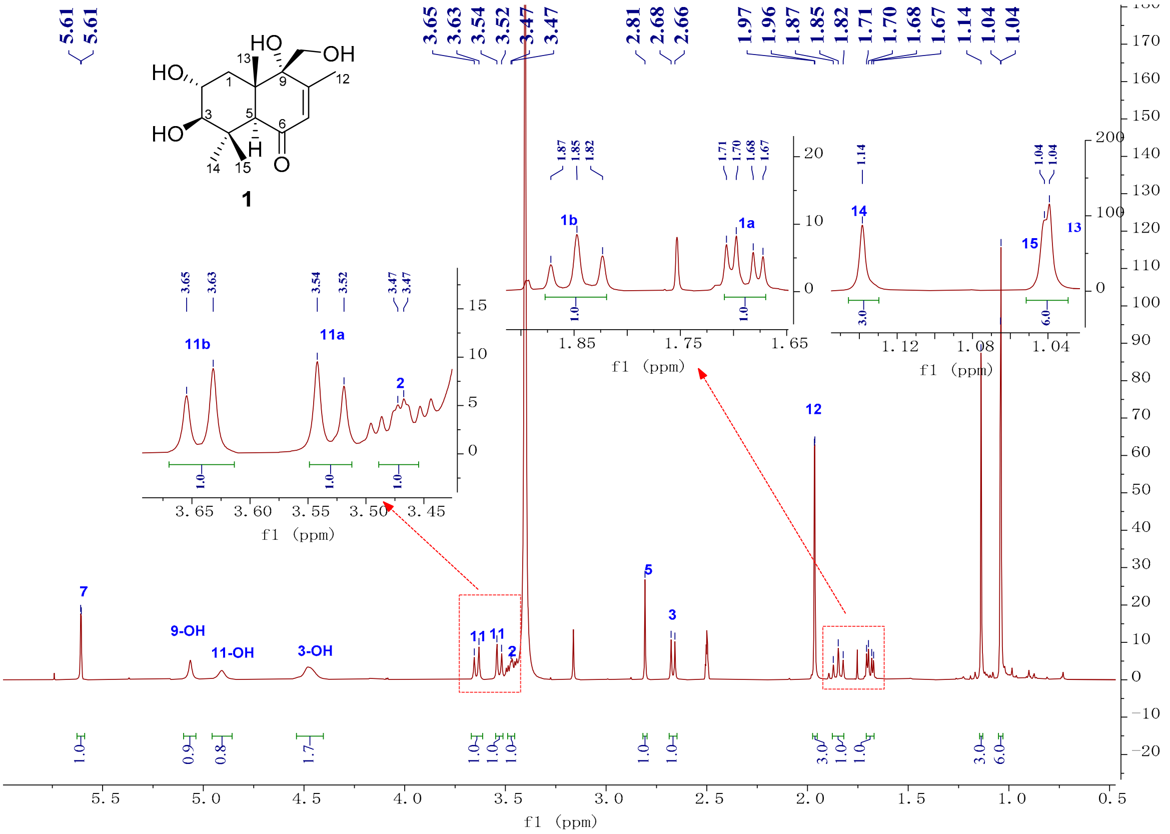

**Figure S4**. The $^{13}$C-NMR and DEPT spectrum of compound **1** in DMSO-$d_6$

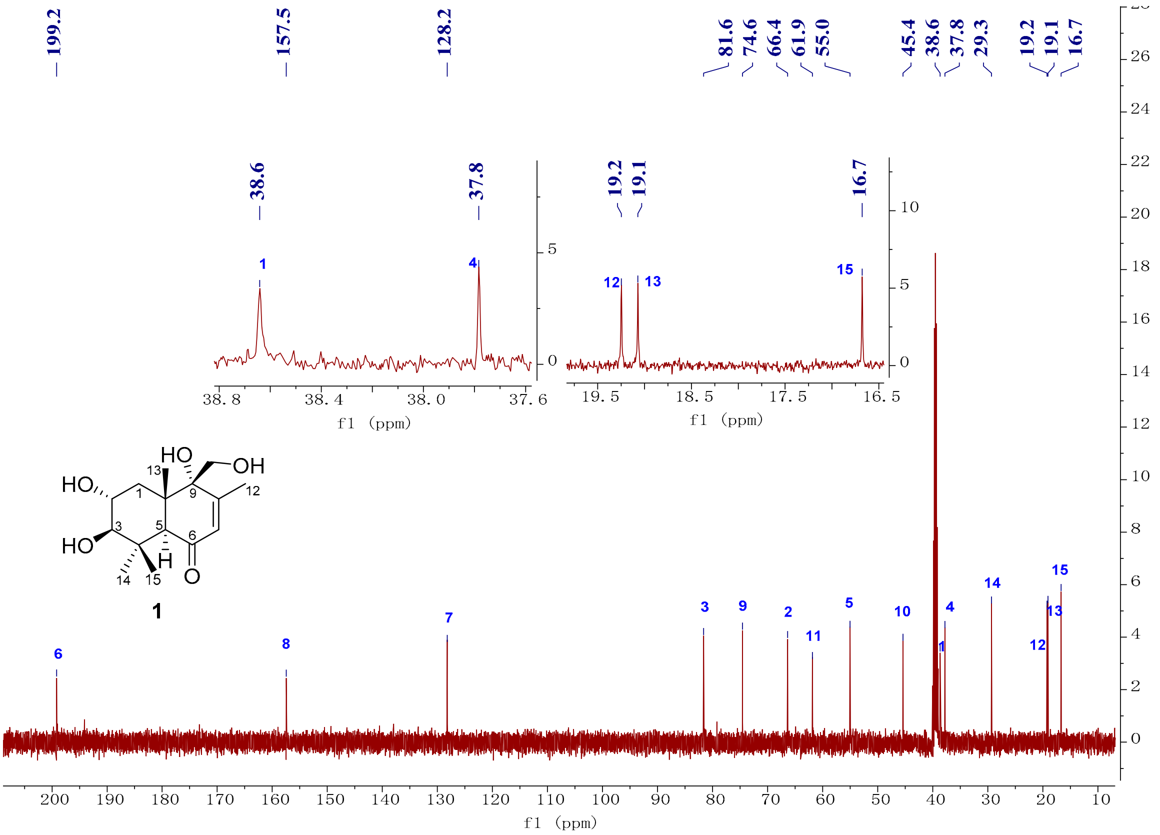


**Figure S5**. The DEPT spectrum of compound **1** in DMSO-$d_6$

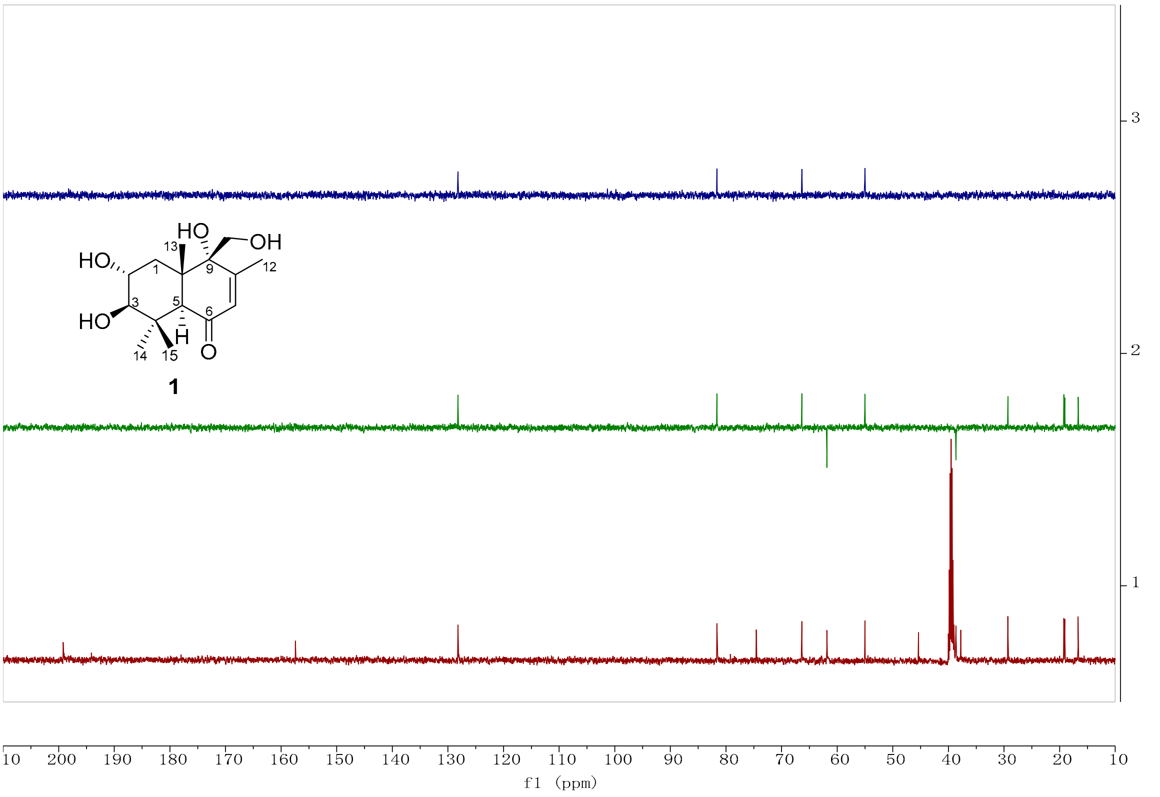

**Figure S6**. The $^{1}$H-$^{1}$H COSY spectrum of compound **1** in DMSO-$d_6$

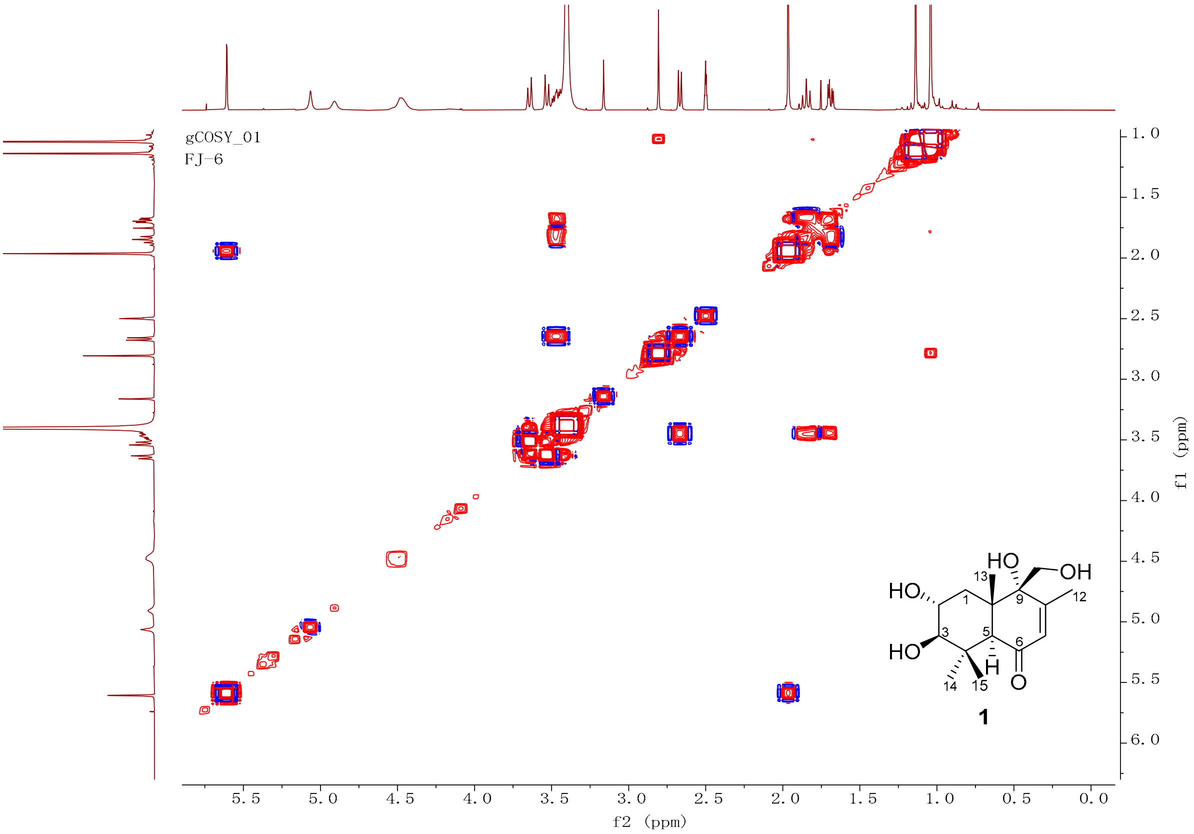


**Figure S7**. The HSQC spectrum of compound **1** in DMSO-$d_6$

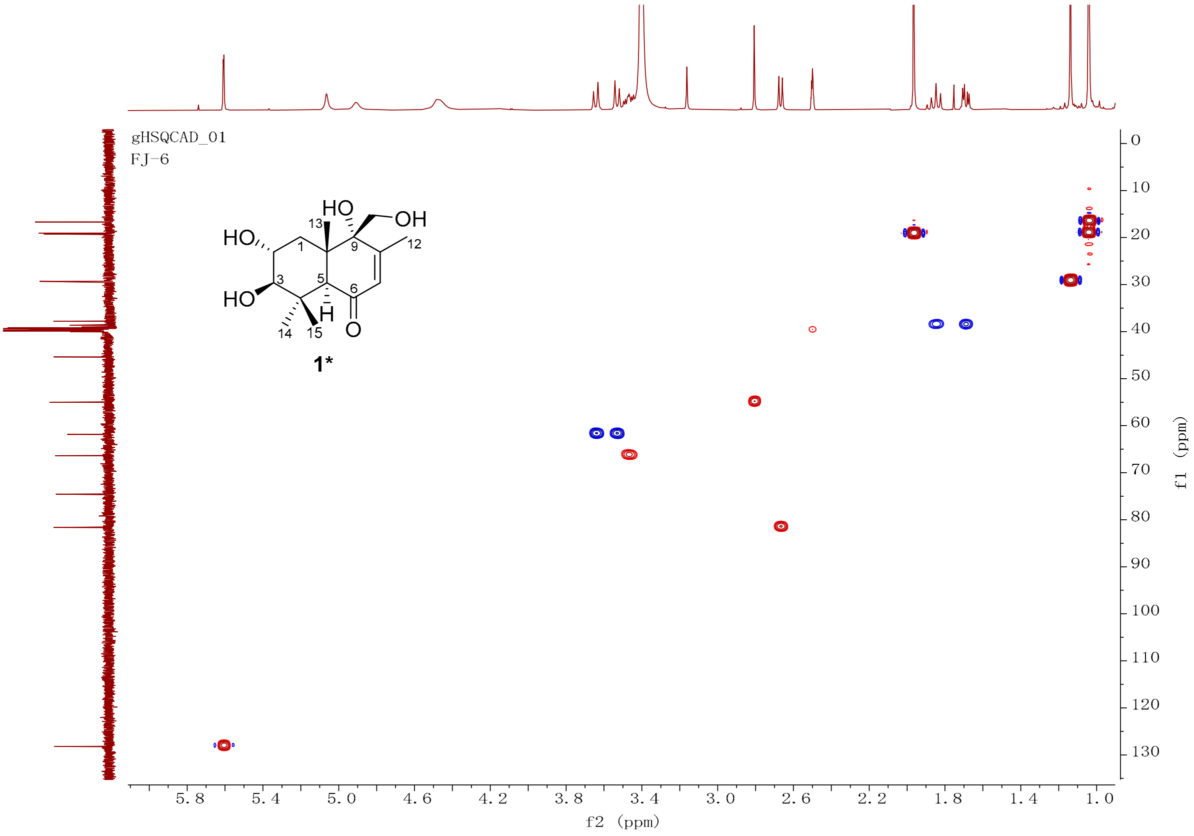

**Figure S8**. The HMBC spectrum of compound **1** in DMSO-$d_6$

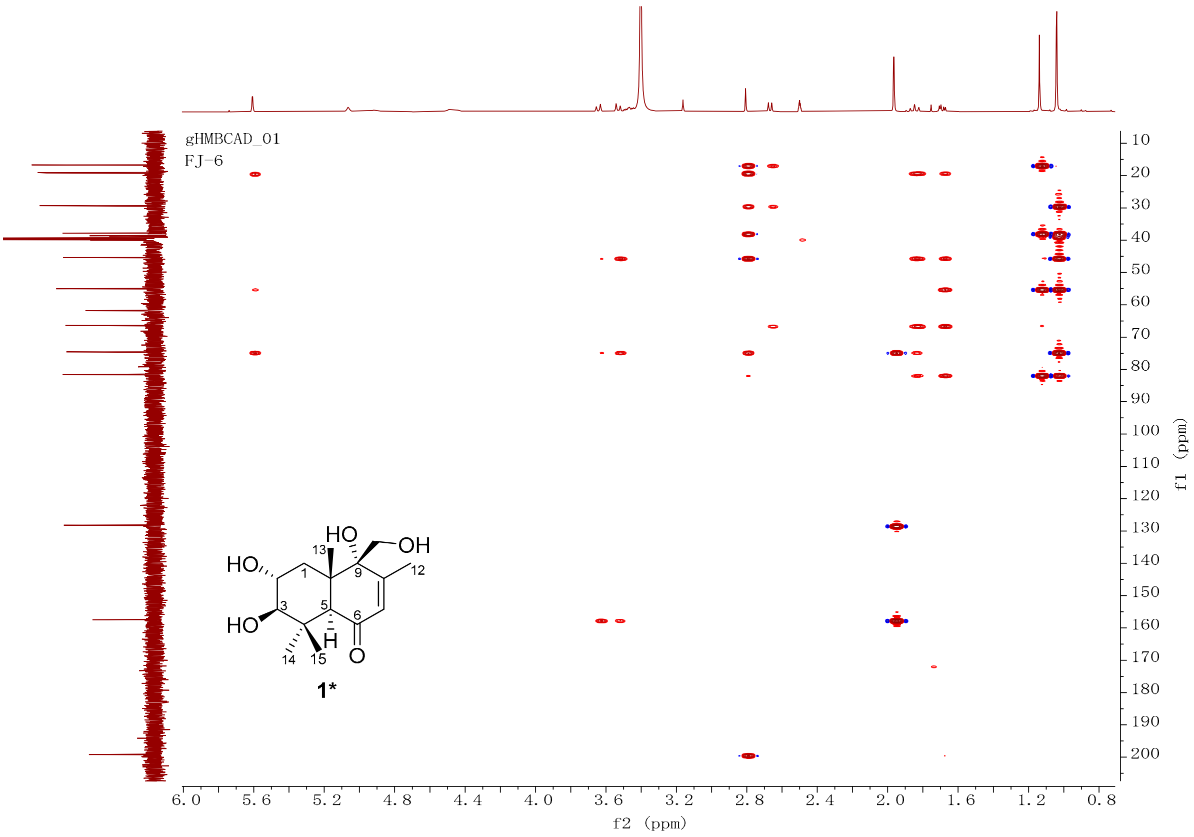


**Figure S9**. The NOESY spectrum of compound **1** in DMSO-$d_6$

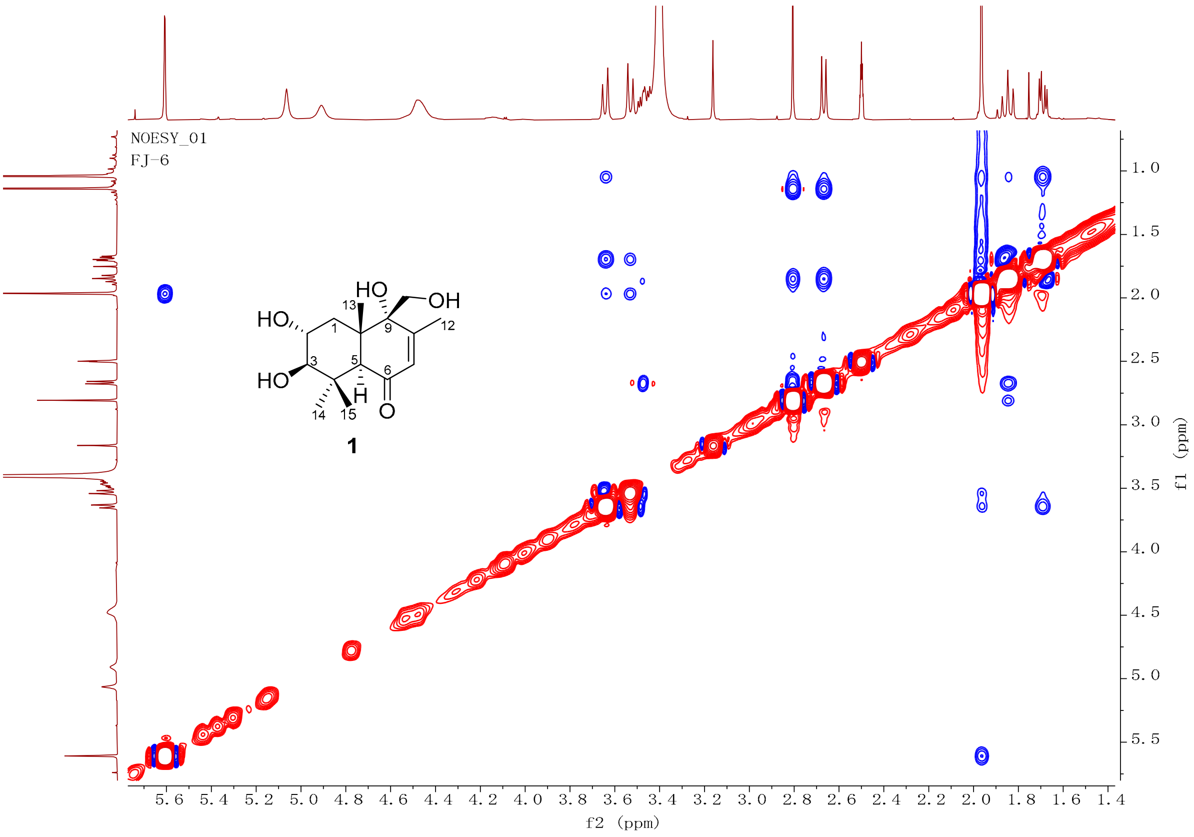

**Figure S10**. The ECD curve of compound **1**

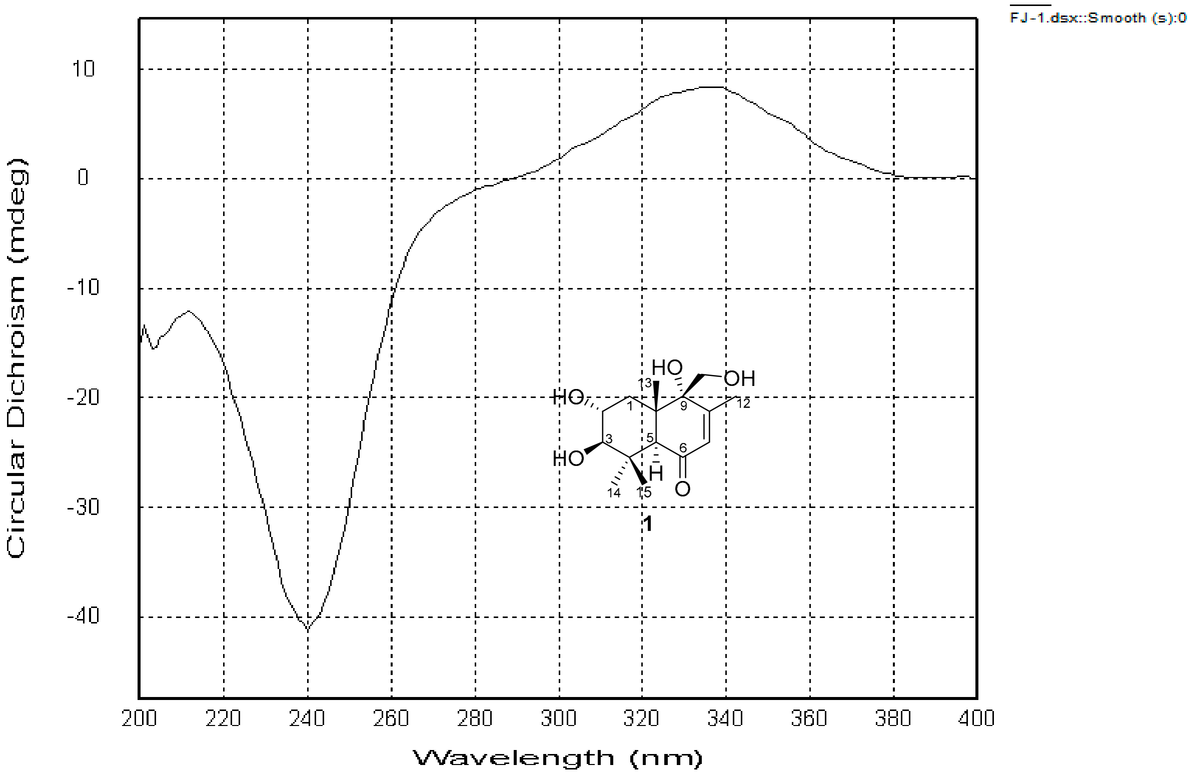


**Figure S11**. The HRESIMS spectrum of compound **2**

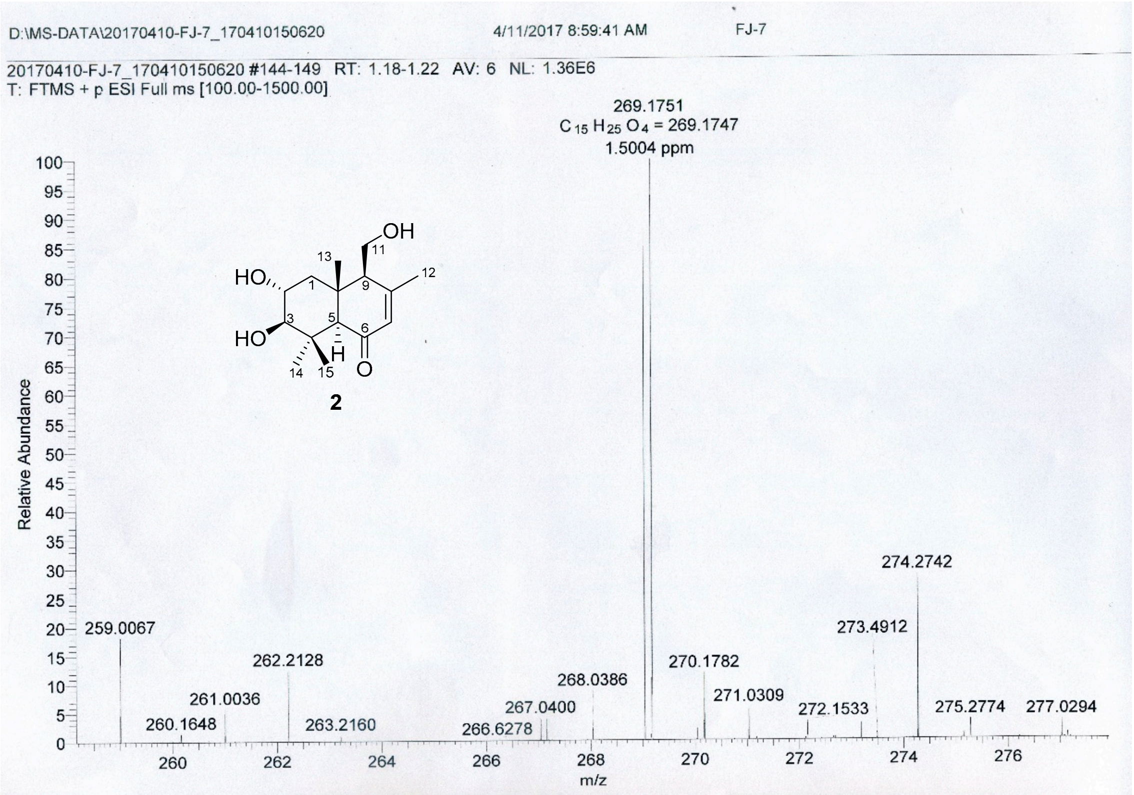

**Figure S12**. The IR spectrum of compound **2**

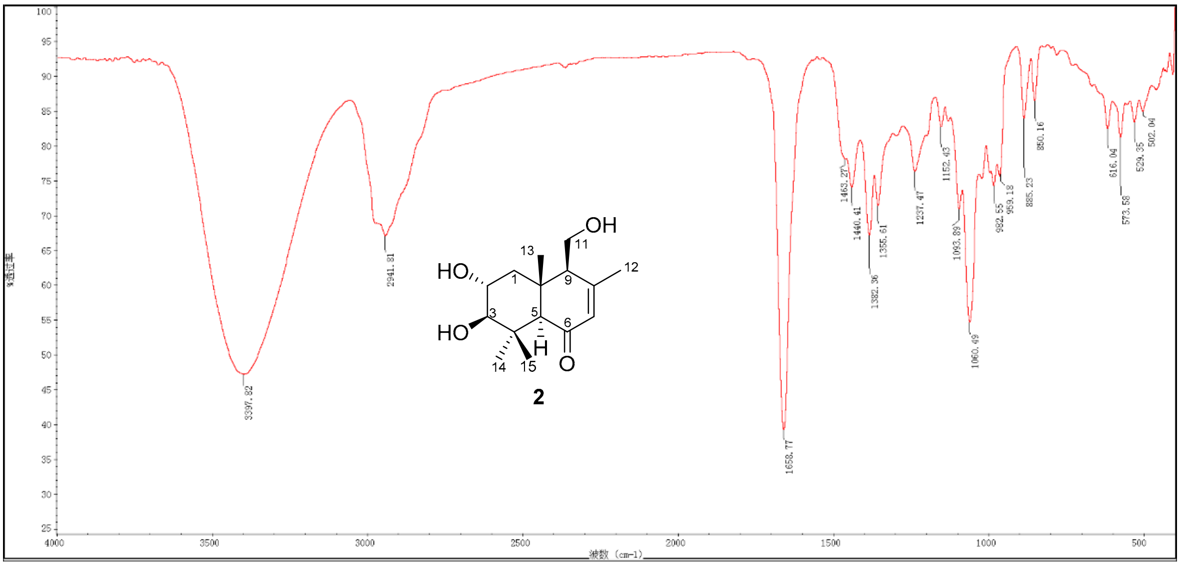

**Figure S13**. The $^{1}$H-NMR spectrum of compound **2** in DMSO-$d_6$

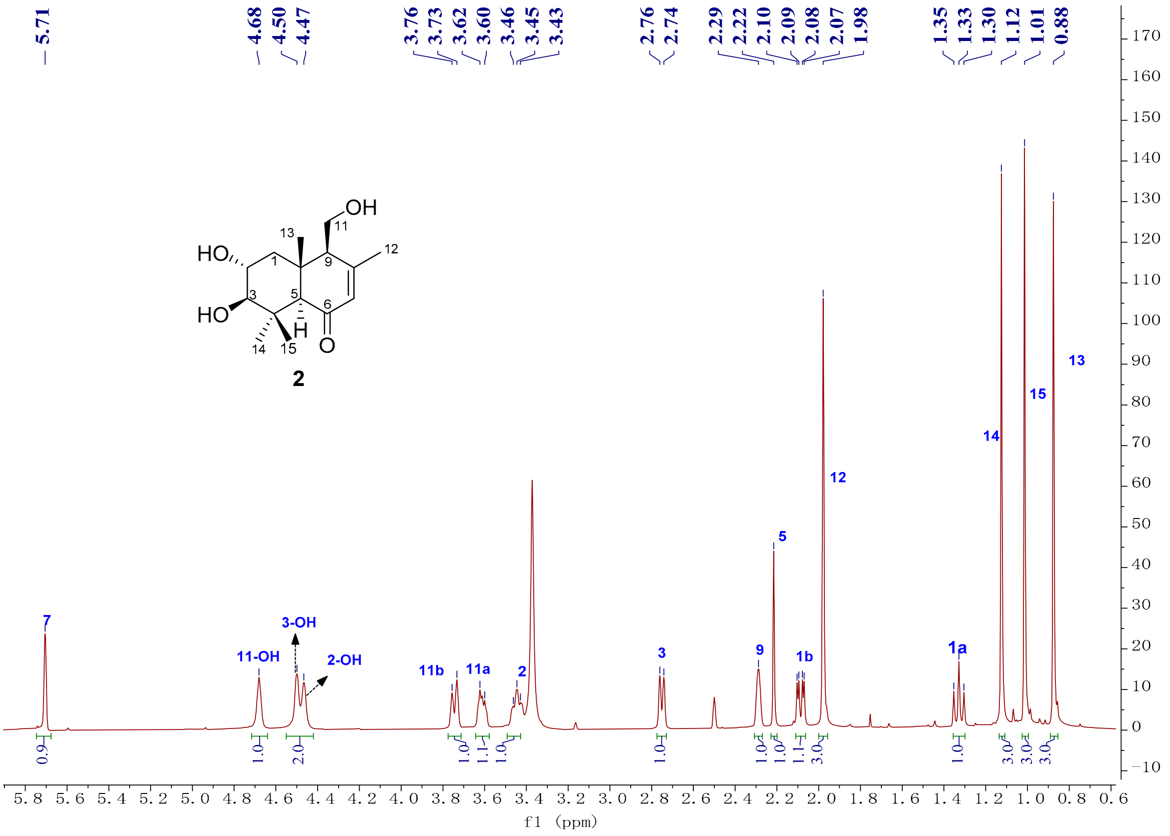

**Figure S14**. The $^{13}$C-NMR spectrum of compound **2** in DMSO-$d_6$

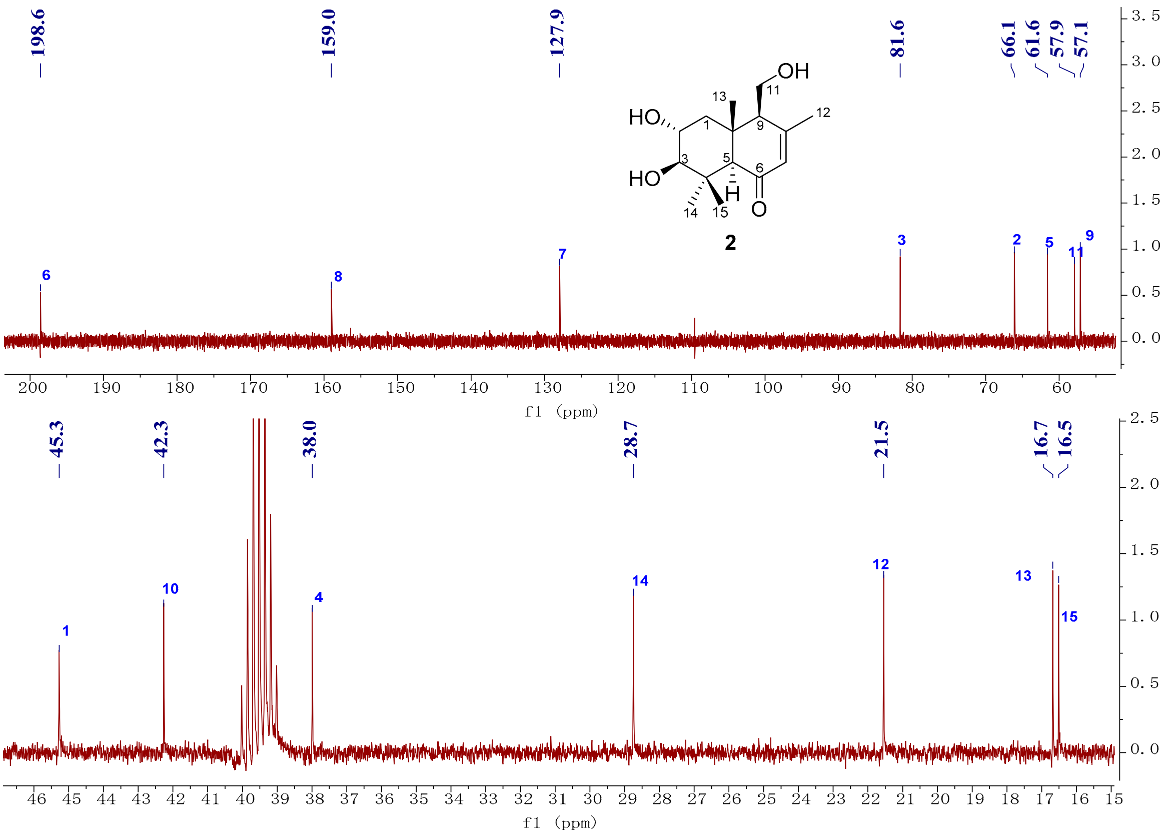


**Figure S15**. The DEPT spectrum of compound **2** in DMSO-$d_6$

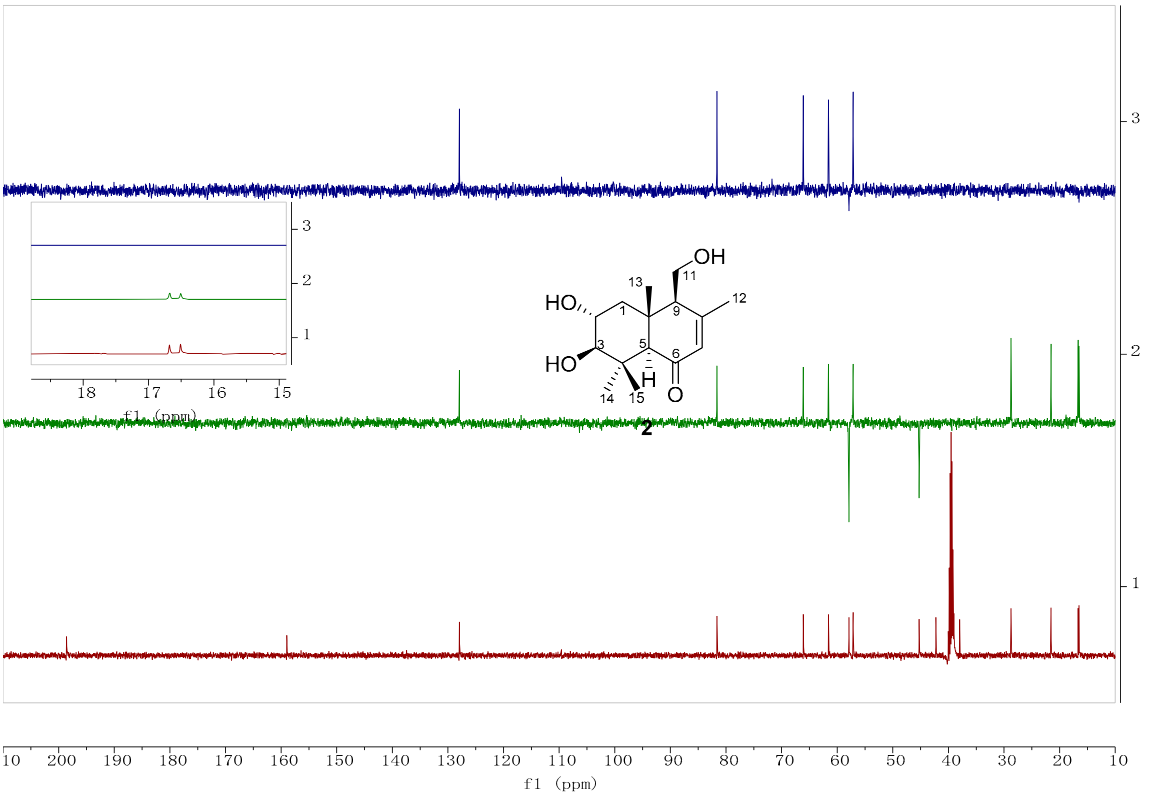

**Figure S16**. The $^1H$-$^1H$ COSY spectrum of compound **2** in DMSO-$d_6$

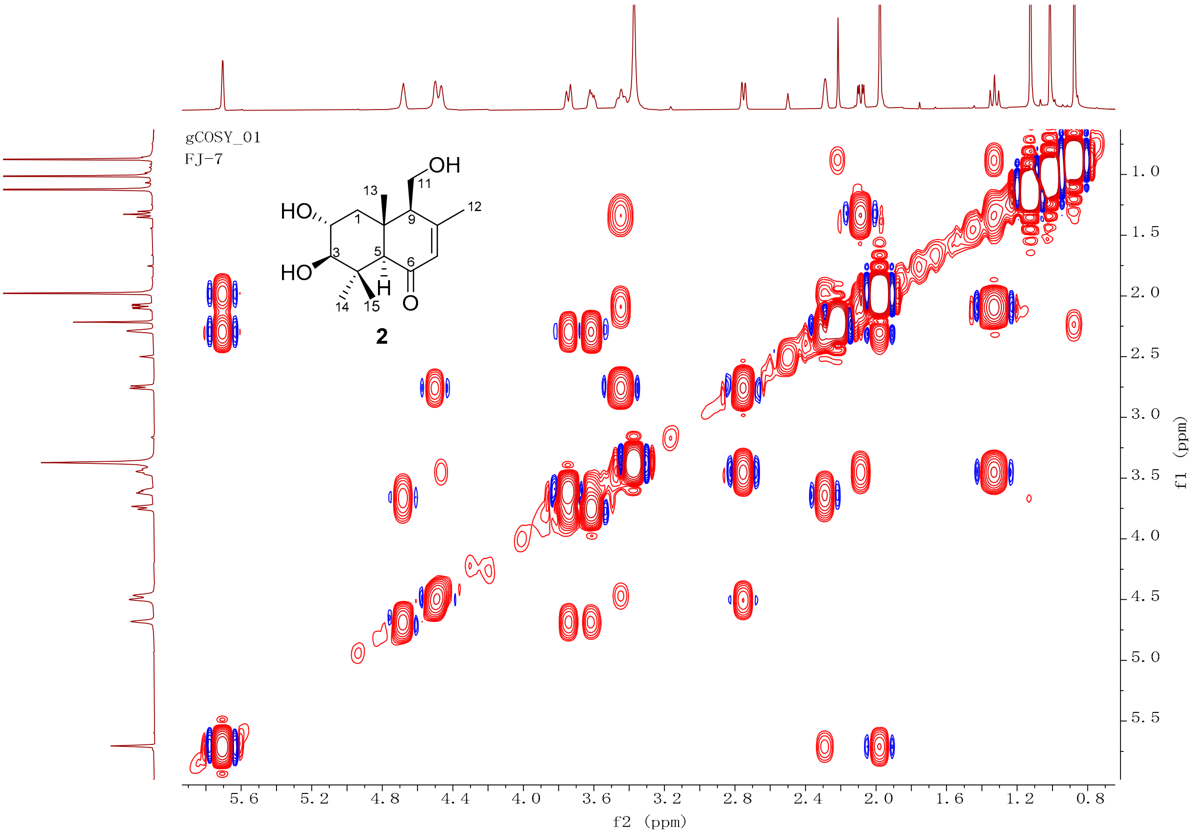


**Figure S17**. The HSQC spectrum of compound **2** in DMSO-$d_6$

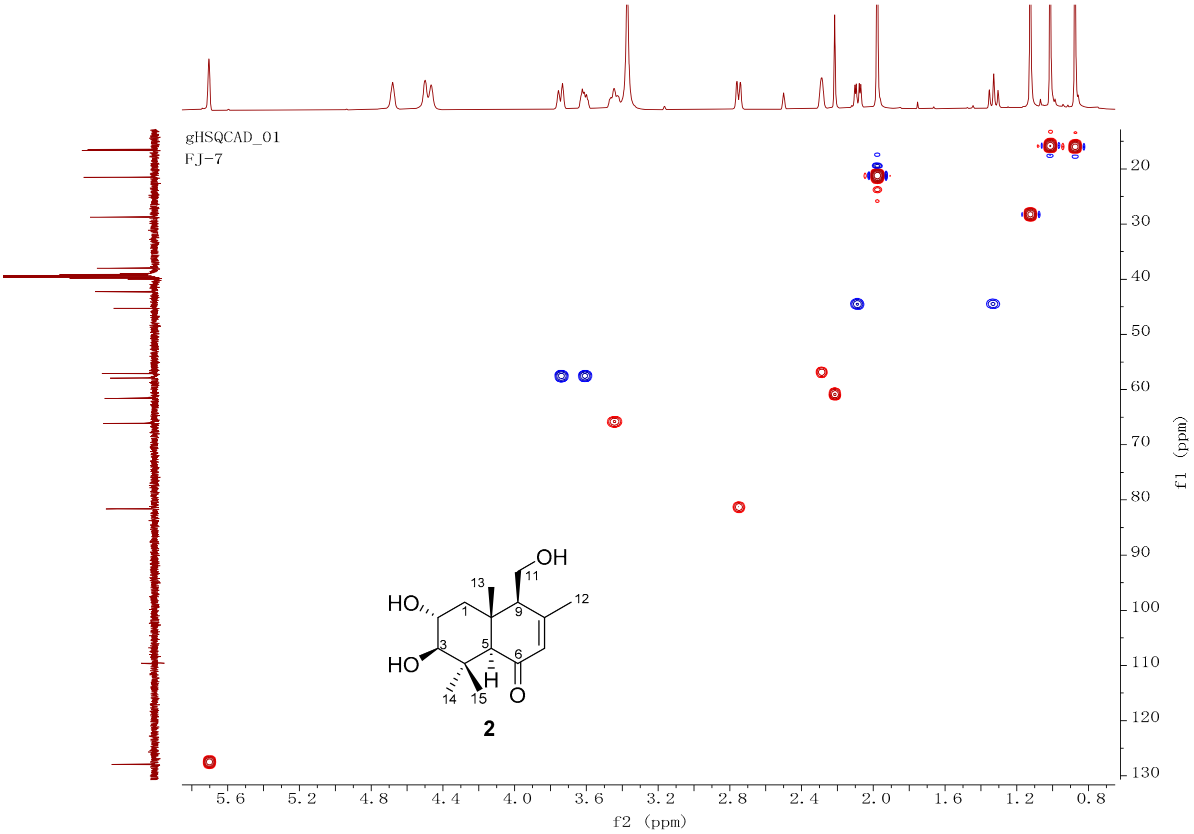

**Figure S18**. The HMBC spectrum of compound **2** in DMSO-$d_6$

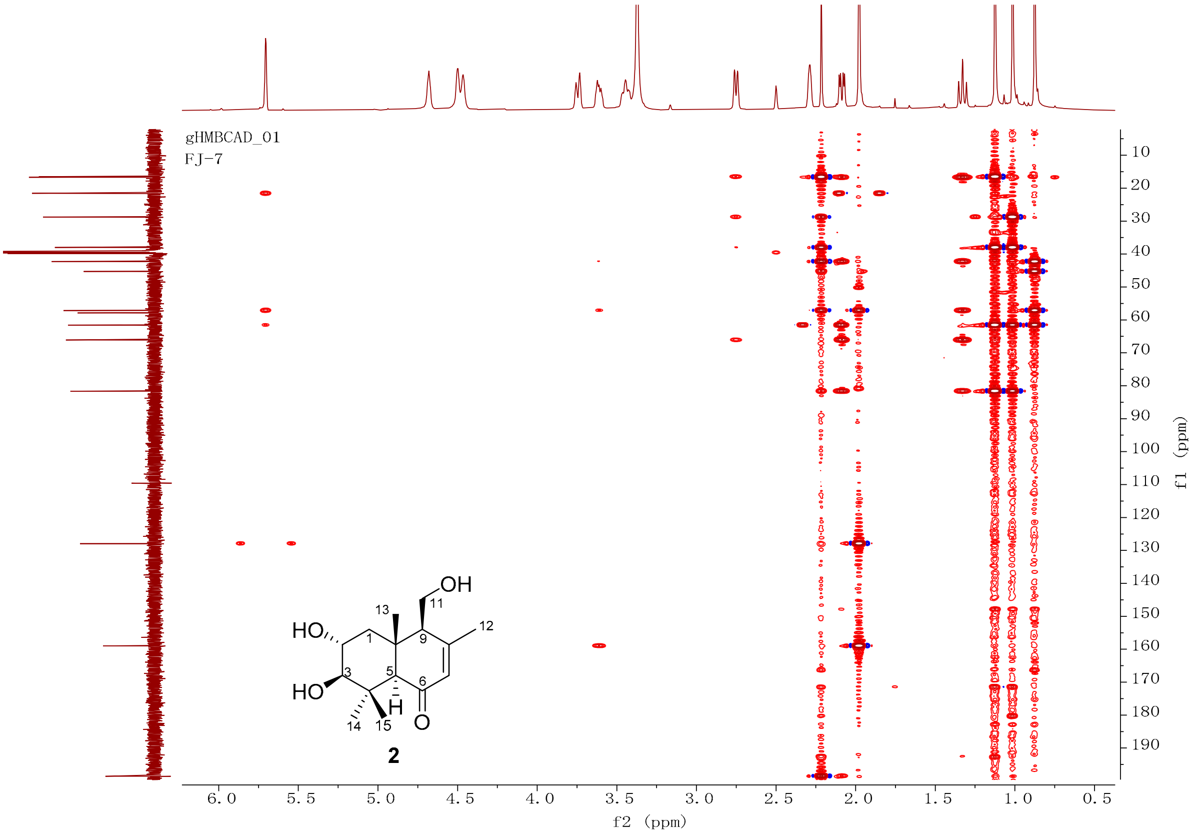


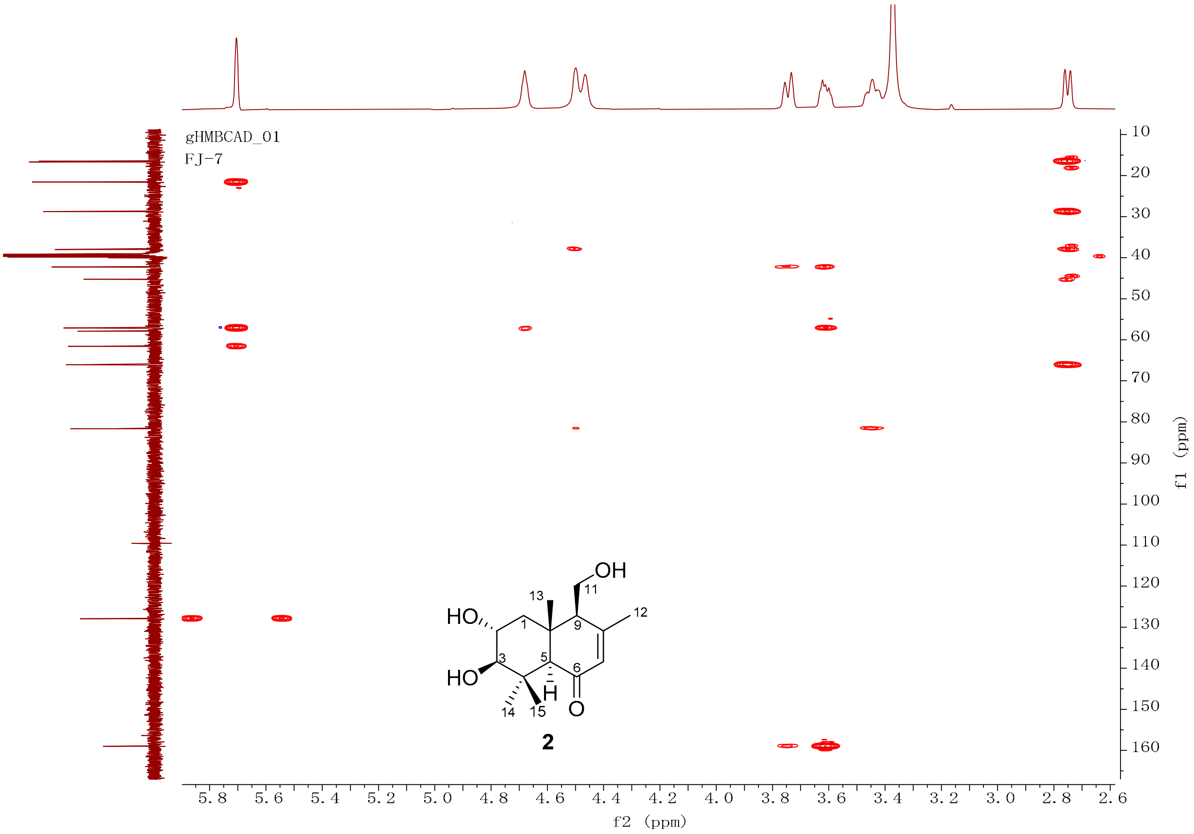

**Figure S19**. The NOESY spectrum of compound **2** in DMSO-$d_6$

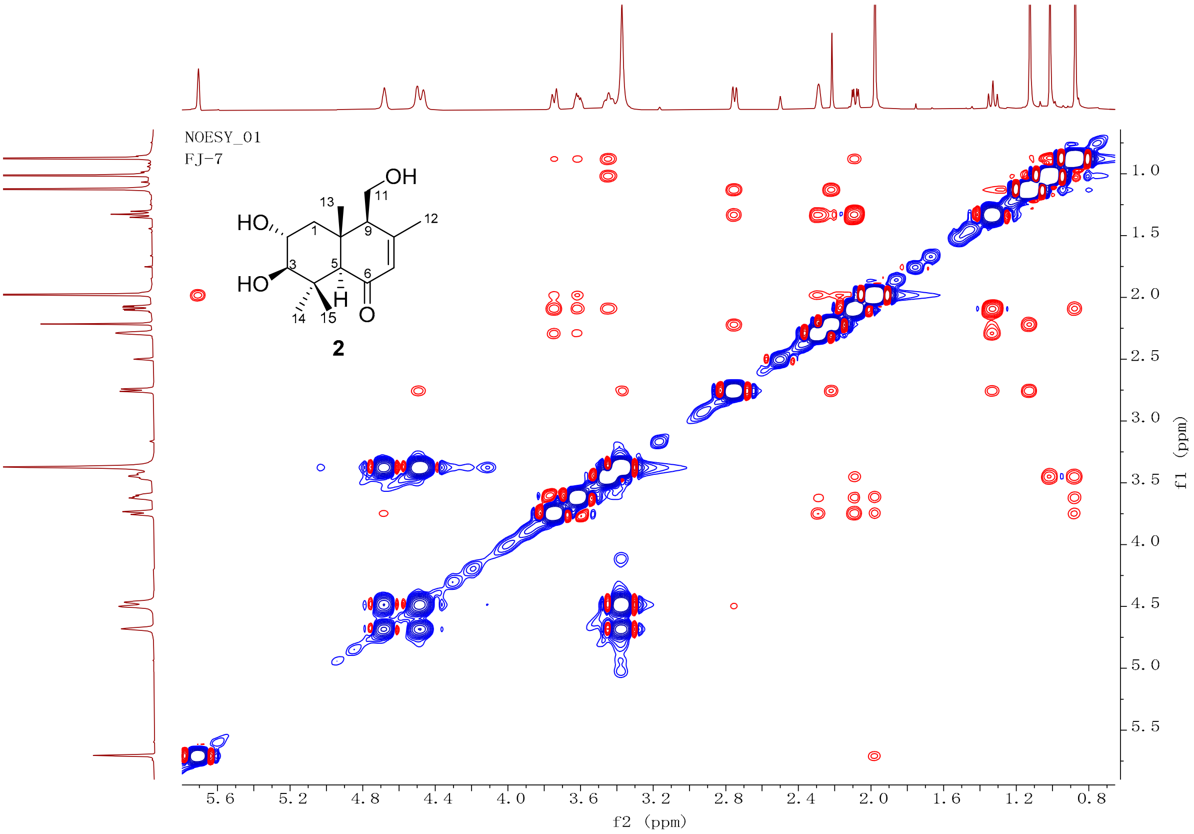


**Figure S20**. ECD curve for the complex of **2** with $Mo_2(OAc)_4$ subtracted from the inherent ECD

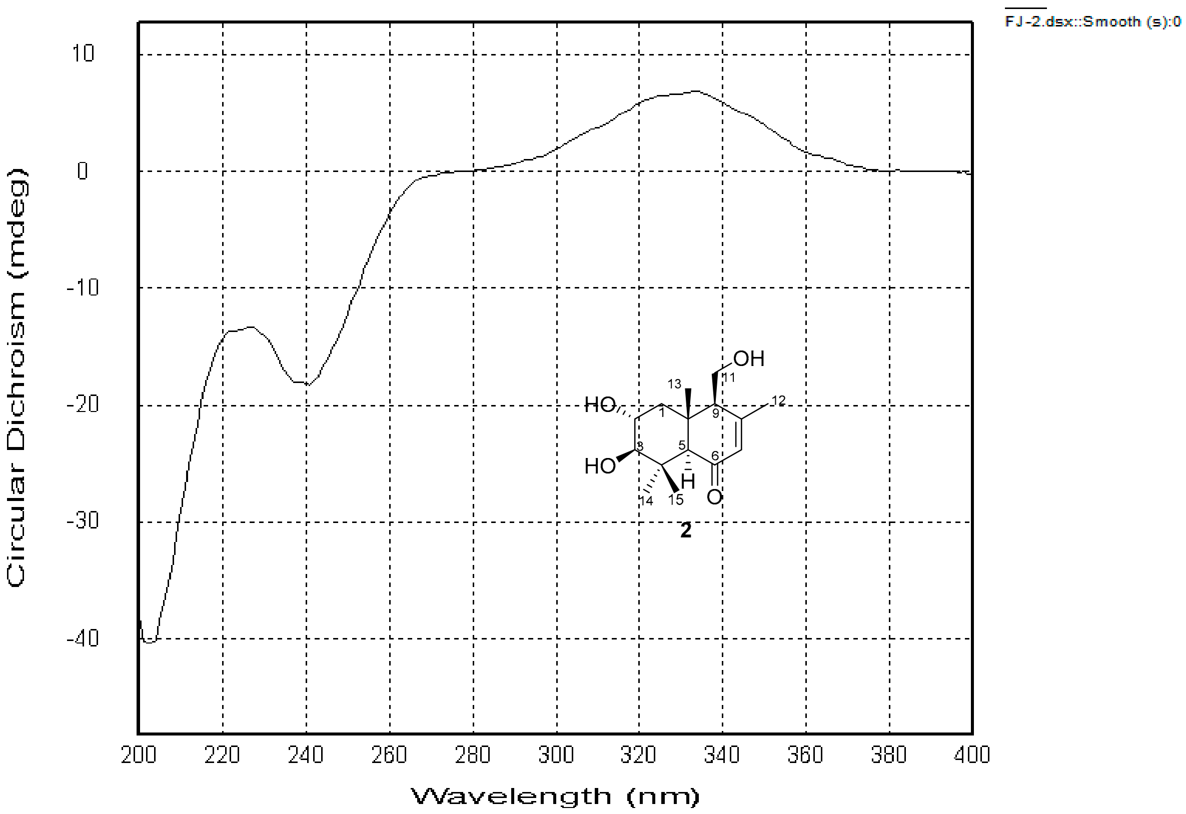

**Figure S21**.The HRESIMS spectrum of compound **3**

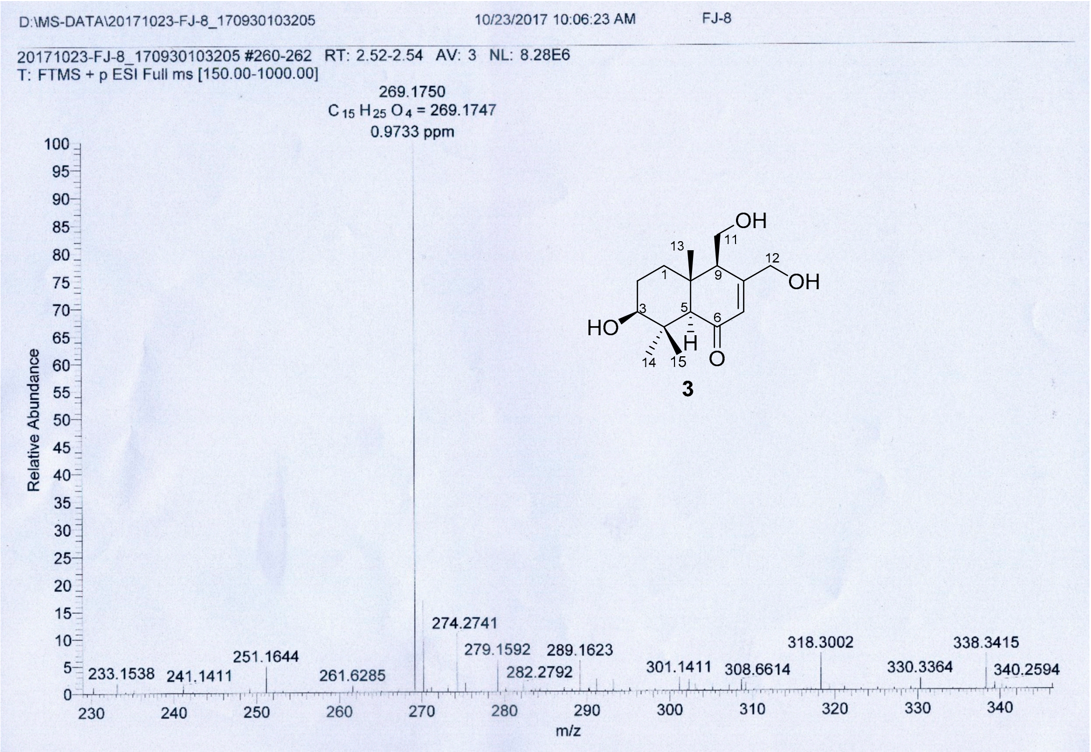


**Figure S22**. The IR spectrum of compound **3**

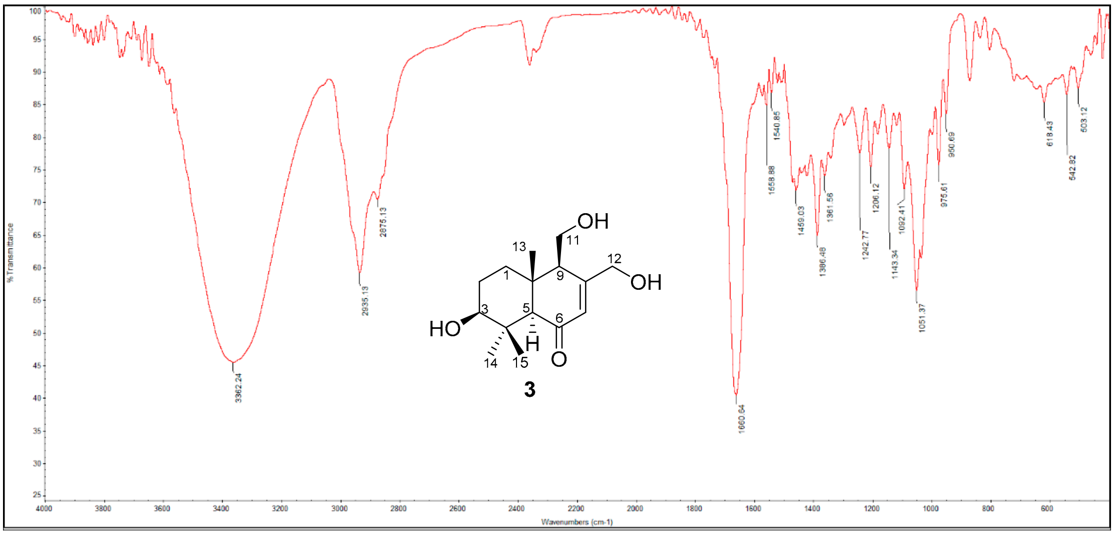

**Figure S23**. The $^{1}$H-NMR spectrum of compound **3** in DMSO-$d_6$

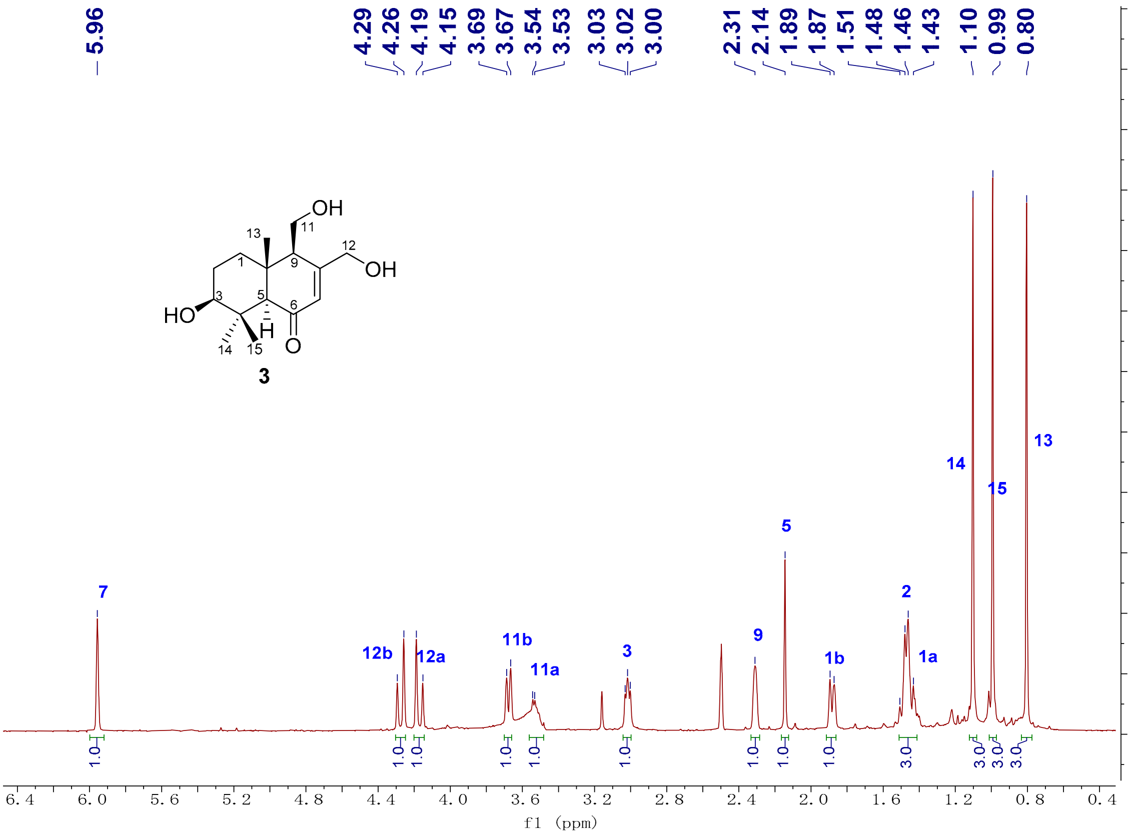


**Figure S24**. The $^{13}$C-NMR spectrum of compound **3** in DMSO-$d_6$

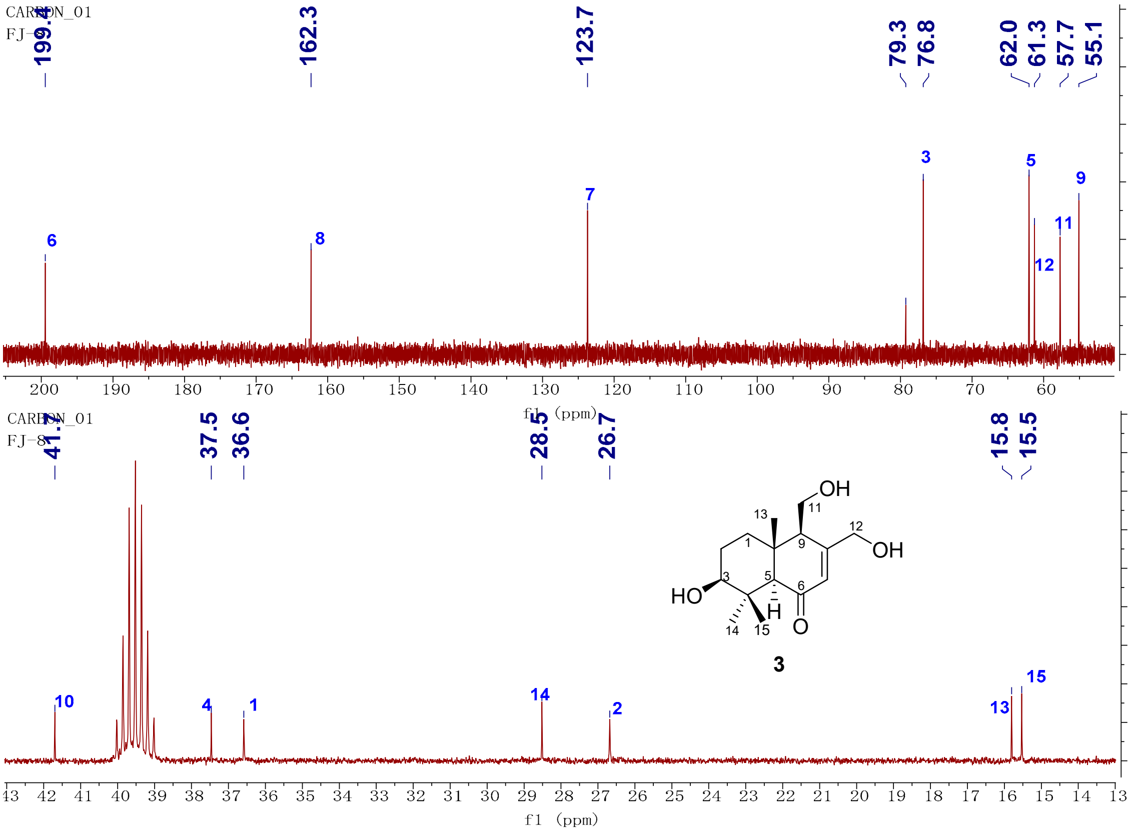

**Figure S25**. The DEPT spectrum of compound **3** in DMSO-$d_6$

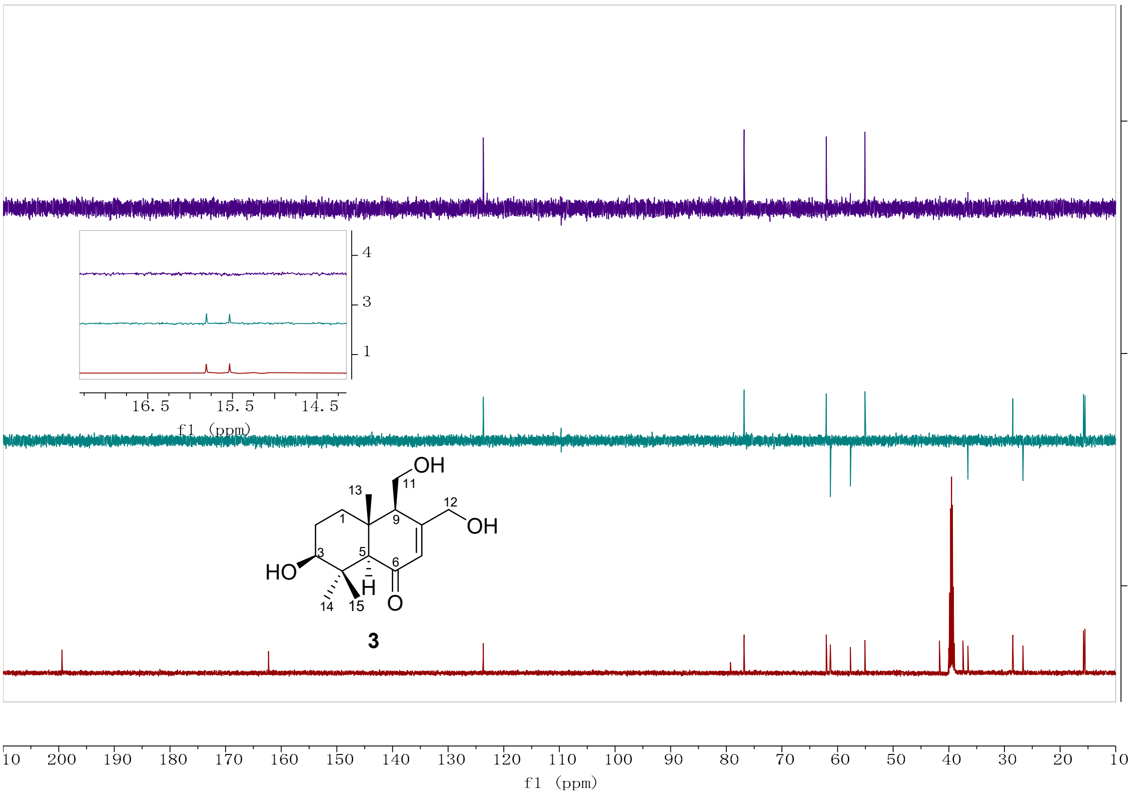


**Figure S26**. The $^{1}$H-$^{1}$H COSY spectrum of compound **3** in DMSO-$d_6$

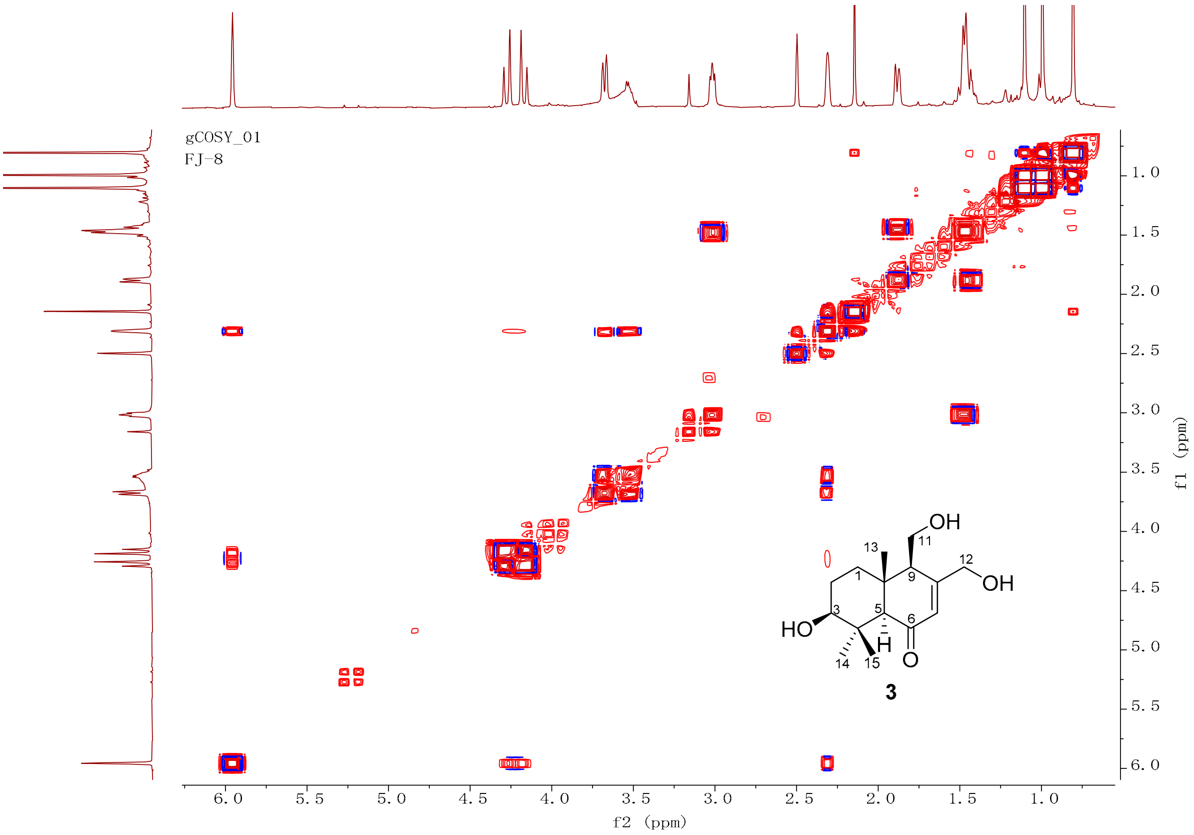

**Figure S27**. The HSQC spectrum of compound **3** in DMSO-$d_6$

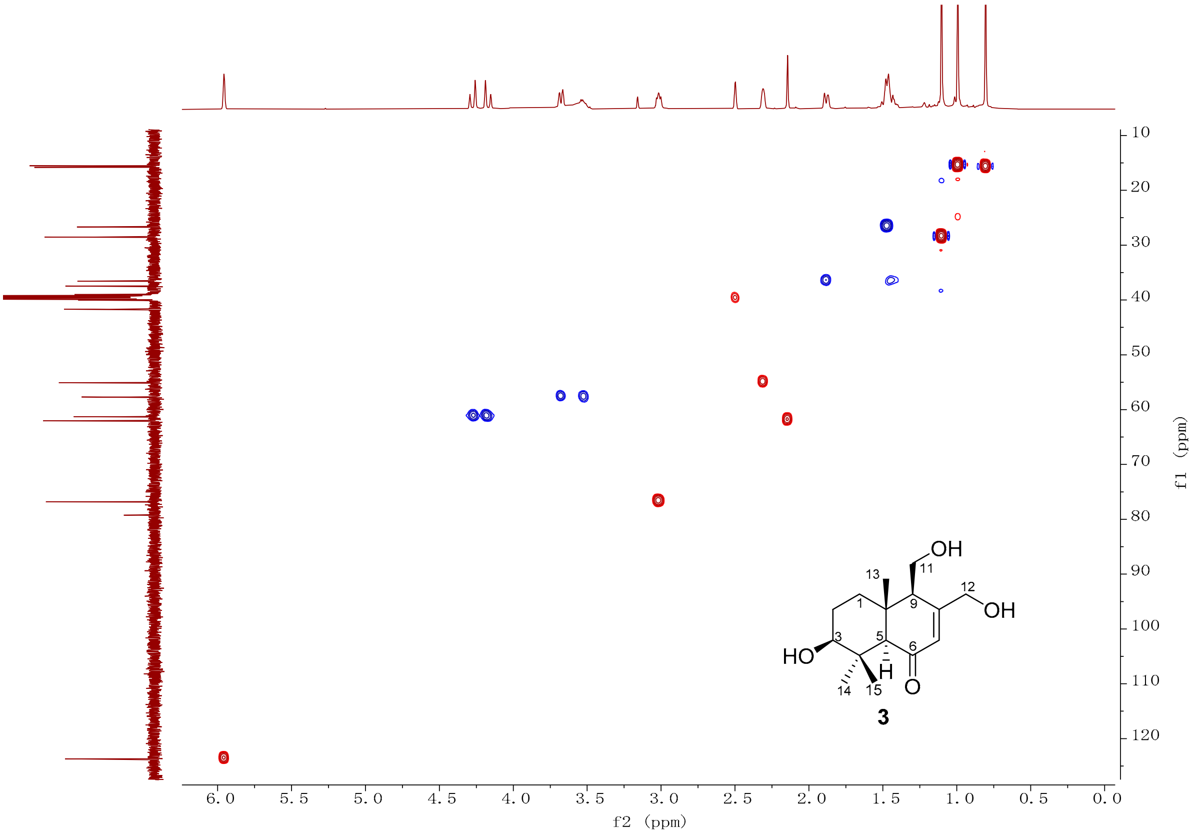


**Figure S28**. The HMBC spectrum of compound **3** in DMSO-$d_6$

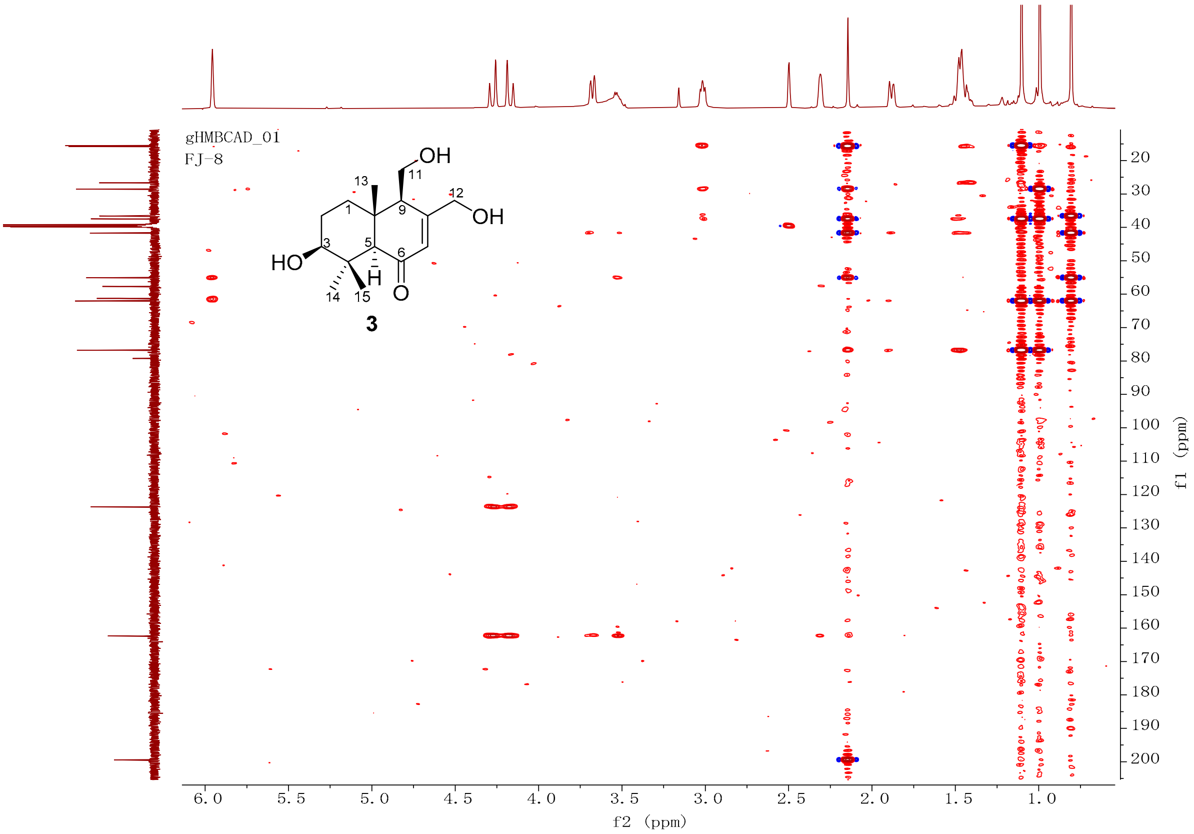

**Figure S29**. NOE difference spectrum of compound **3** in DMSO-$d_6$

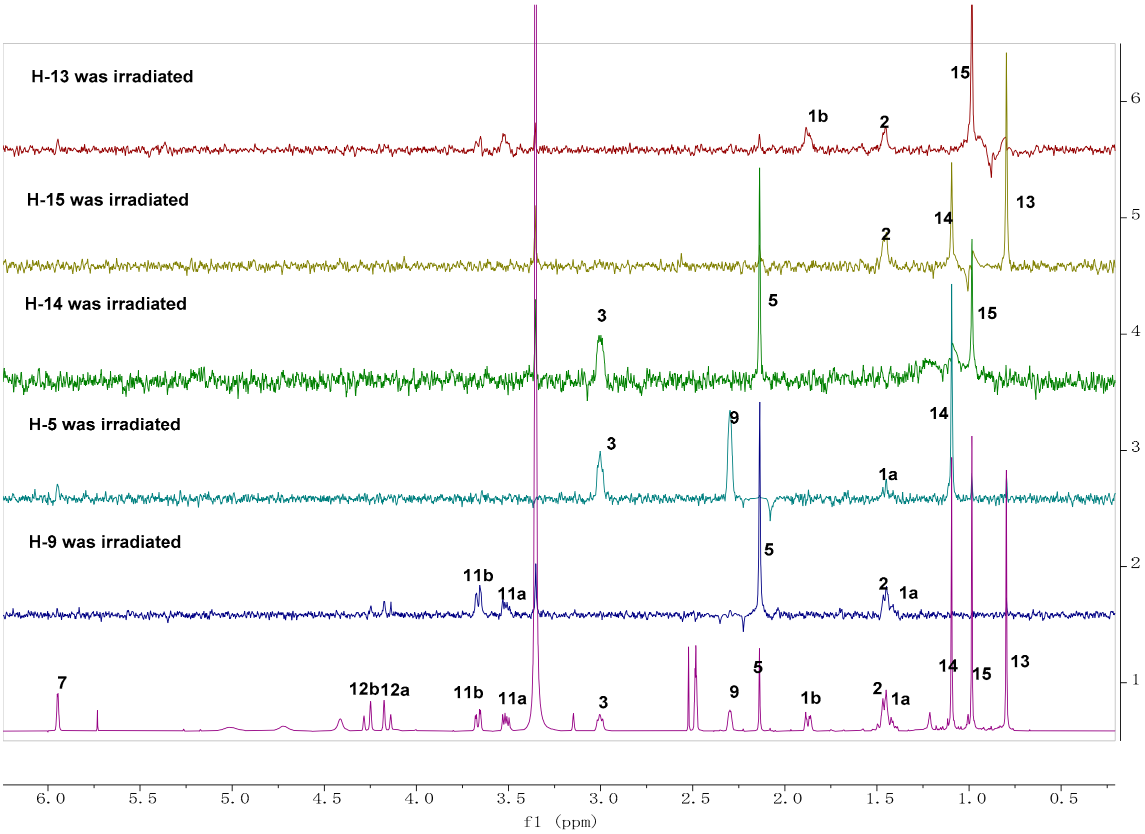


**Figure S30**. The ECD curve of compound **3**

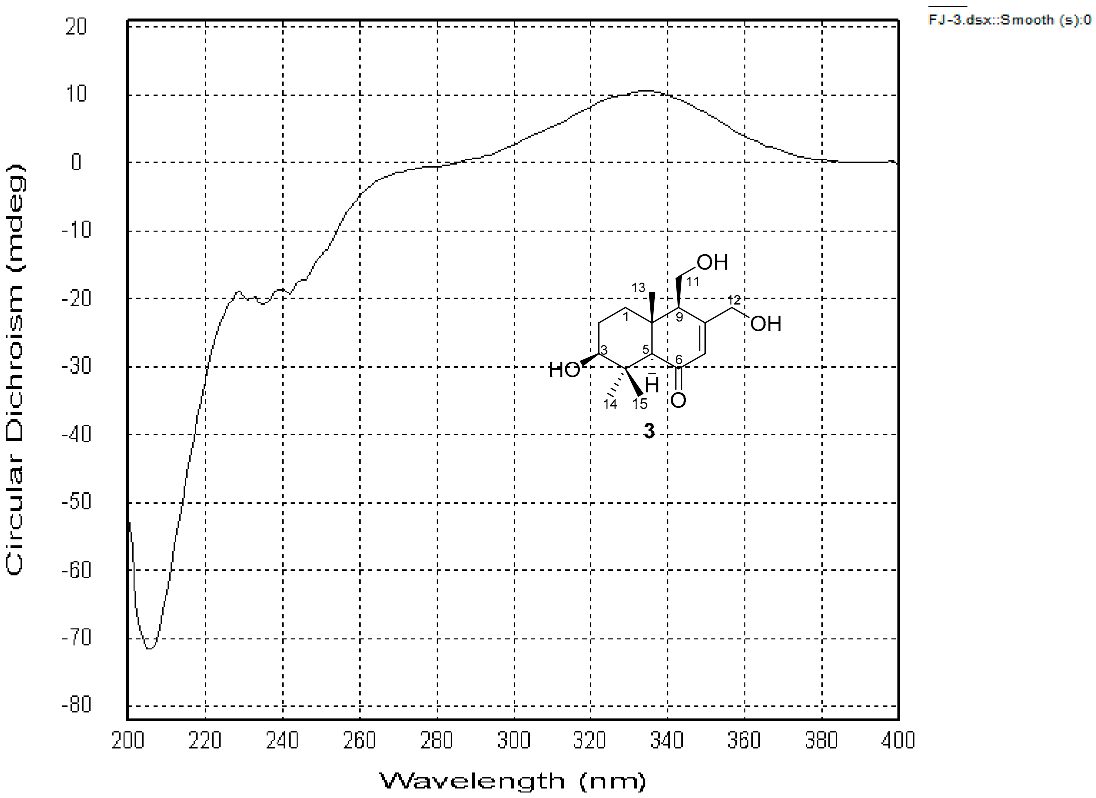

**Figure S31**. The ECD curves of **1**–**3** and **7**

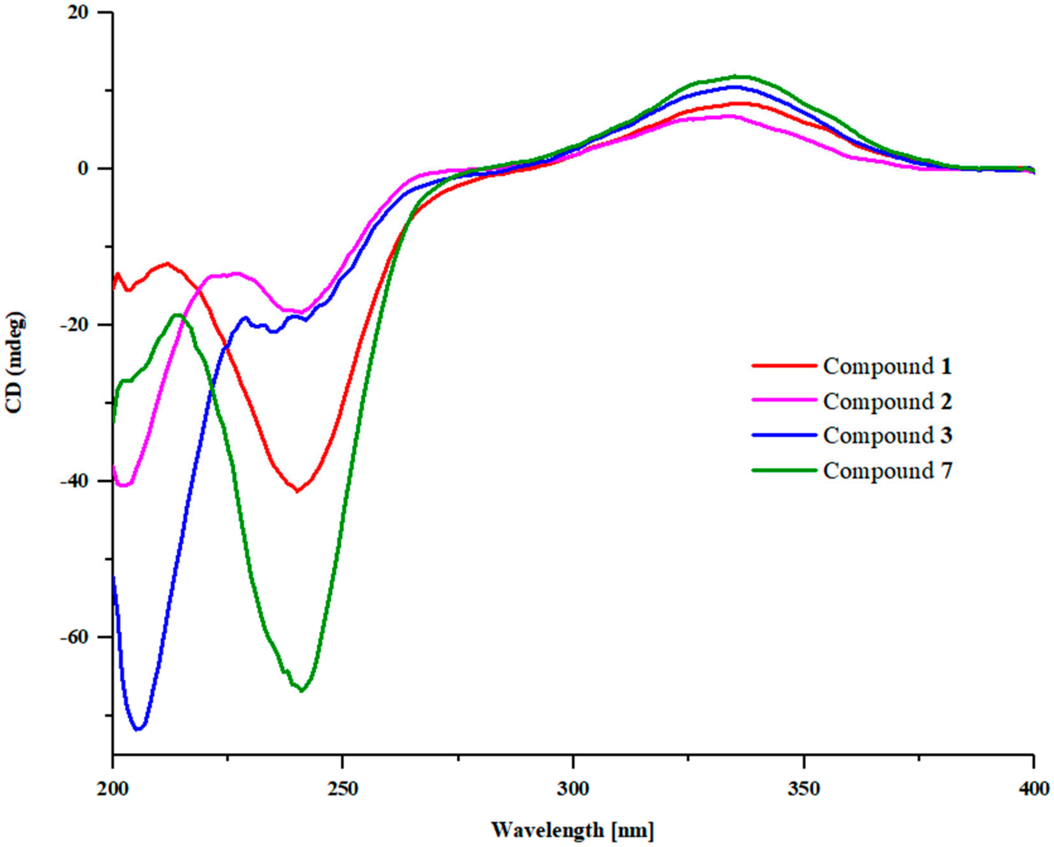


**Figure S32**. The HRESIMS spectrum of compound **4**

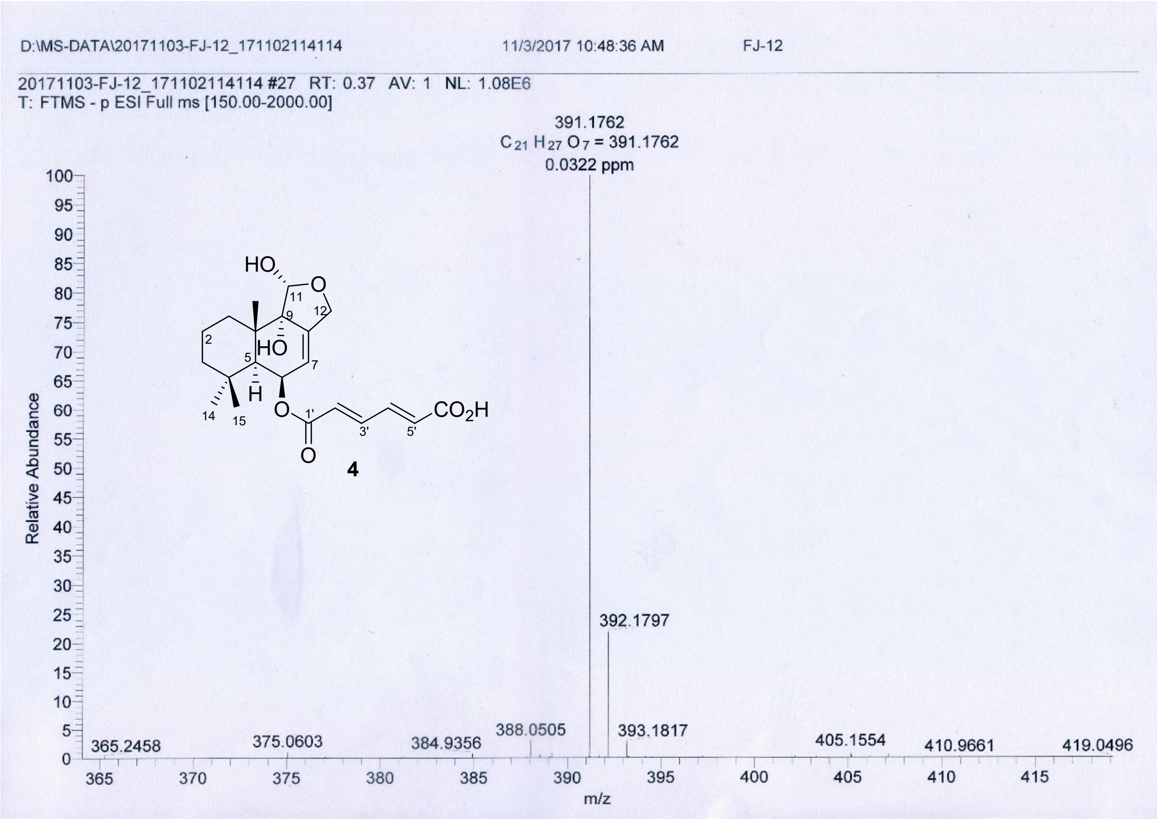

**Figure S33**. The IR spectrum of compound **4**

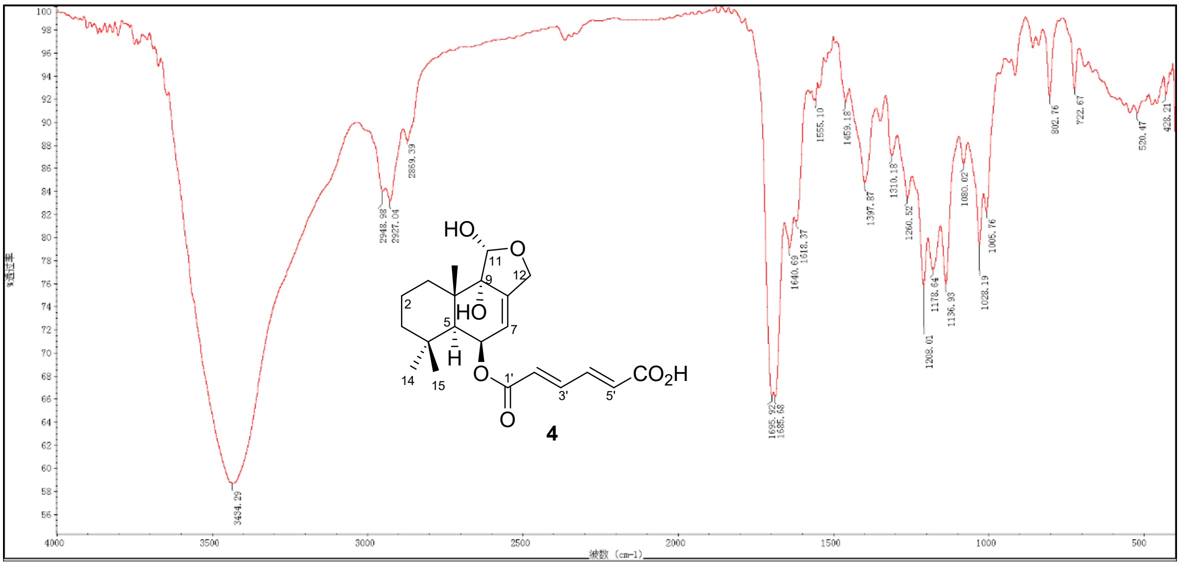

**Figure S34**. The $^{1}$H-NMR spectrum of compound **4** in DMSO-$d_6$

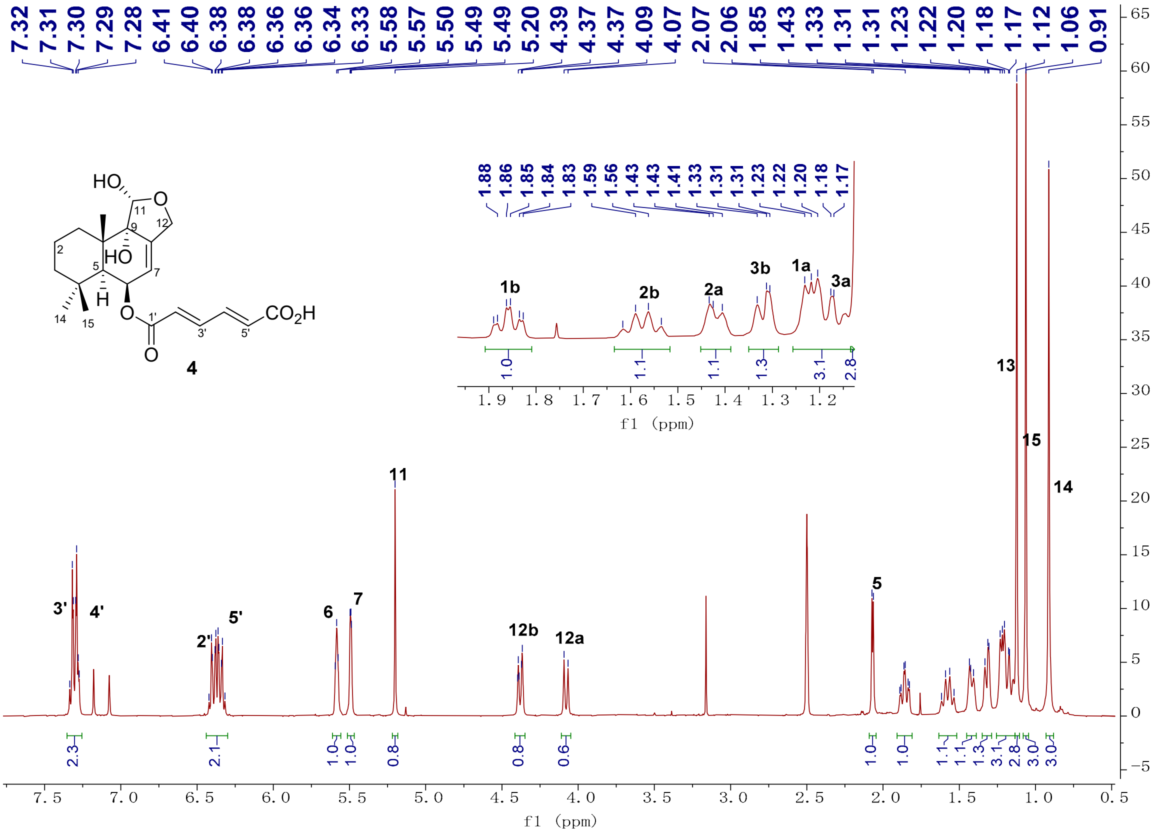

**Figure S35**. The $^{13}$C-NMR spectrum of compound **4** in DMSO-$d_6$

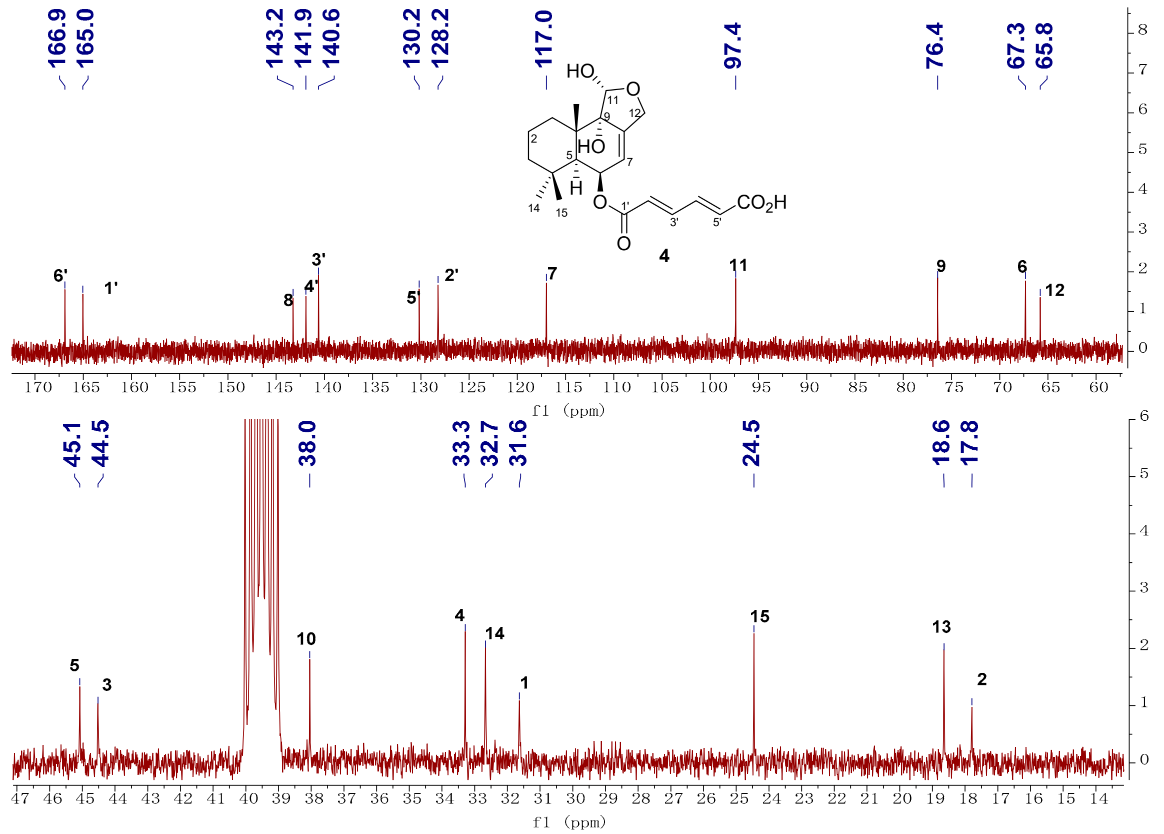


**Figure S36**. The DEPT spectrum of compound **4** in DMSO-$d_6$

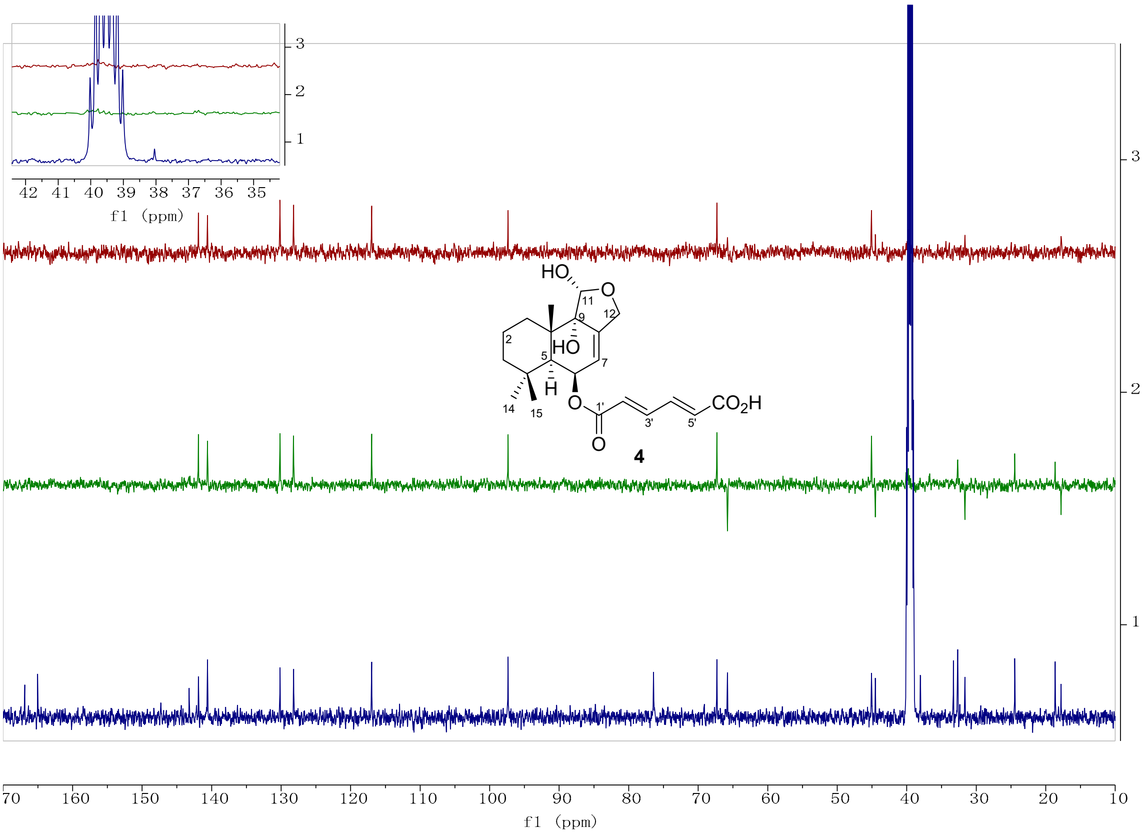

**Figure S37**. The $^1H$-$^1H$ COSY spectrum of compound **4** in DMSO-$d_6$

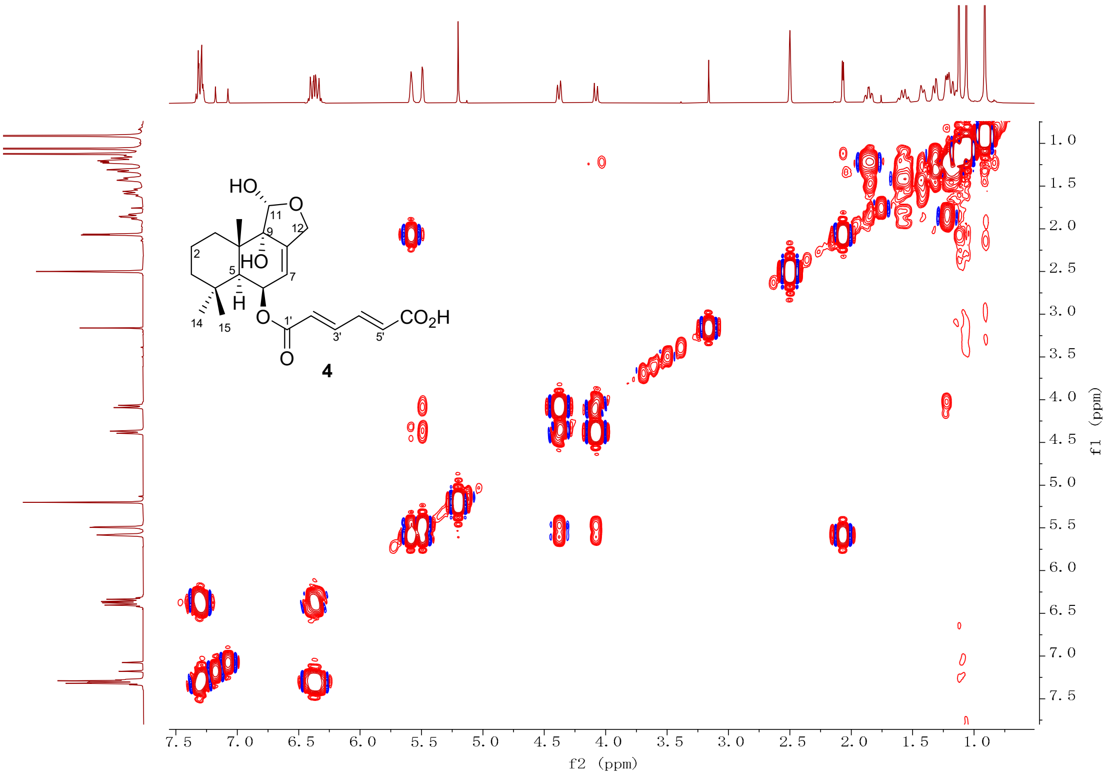


**Figure S38**. The HSQC spectrum of compound **4** in DMSO-$d_6$

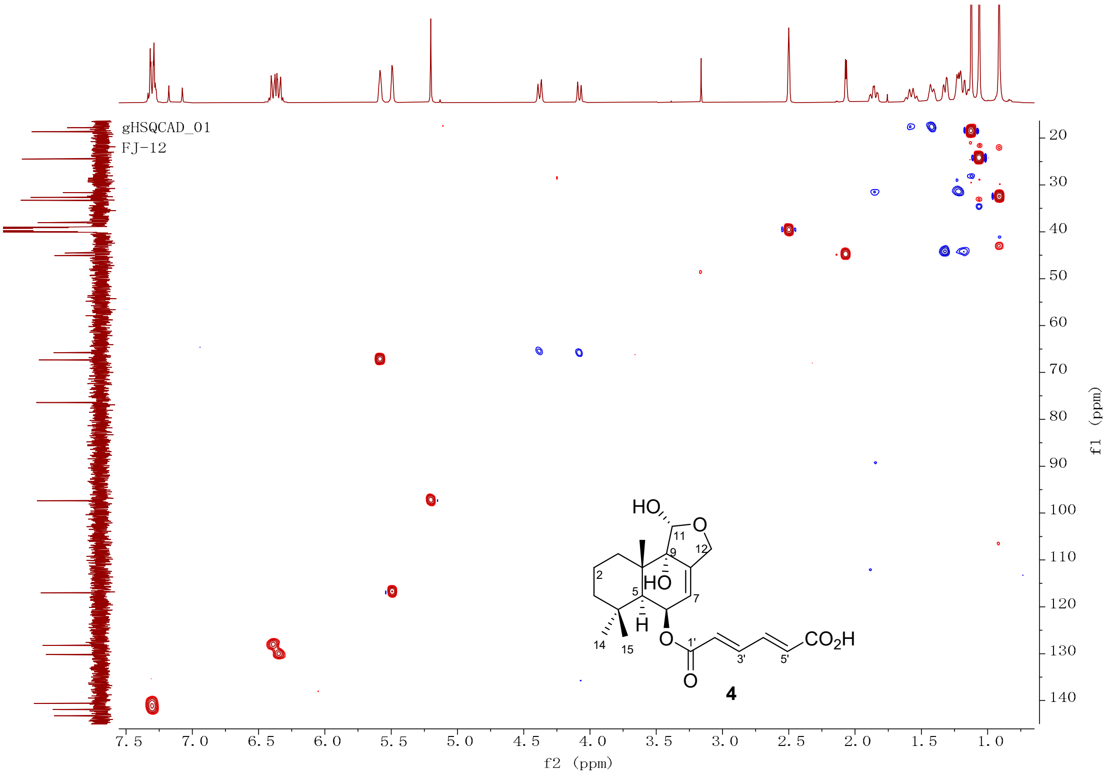

**Figure S39**. The HMBC spectrum of compound **4** in DMSO-$d_6$

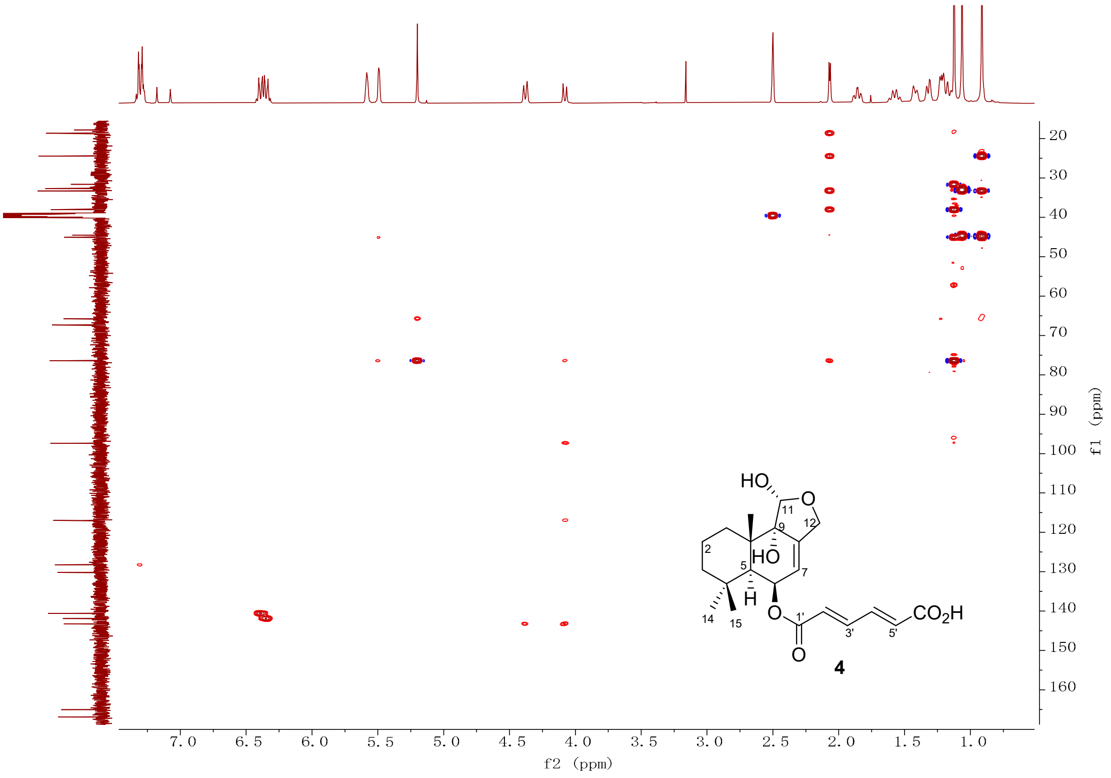

**Figure S40**. The NOESY spectrum of compound **4** in DMSO-$d_6$

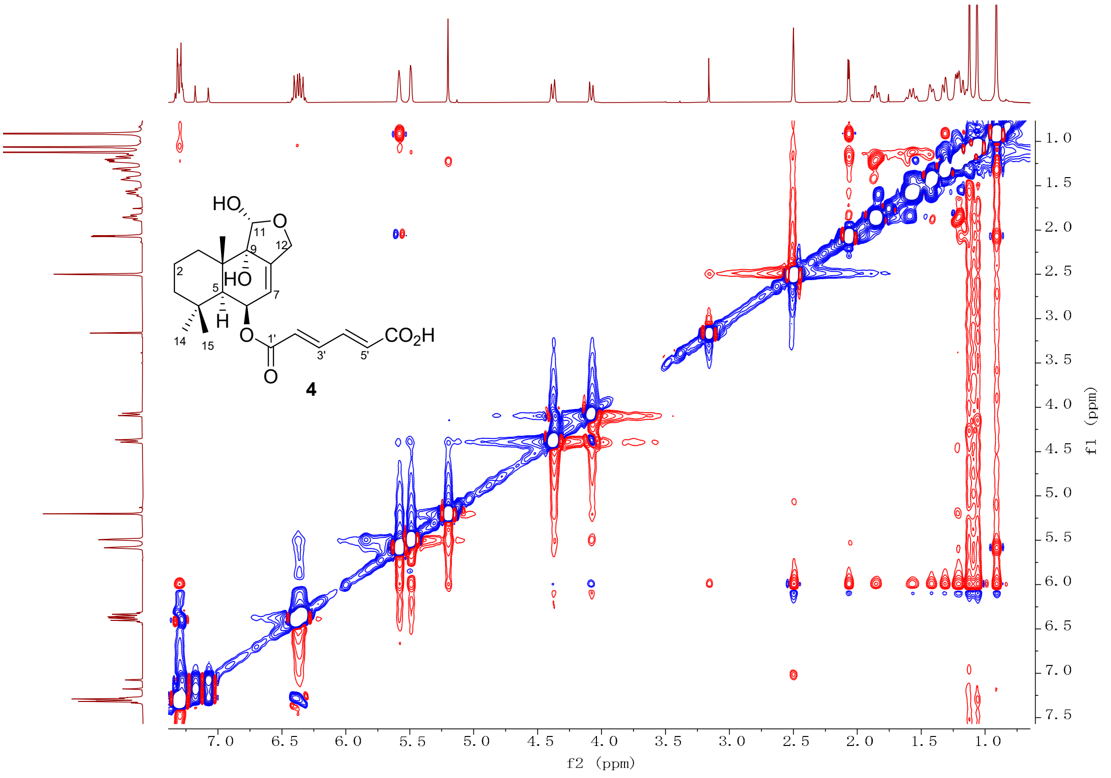

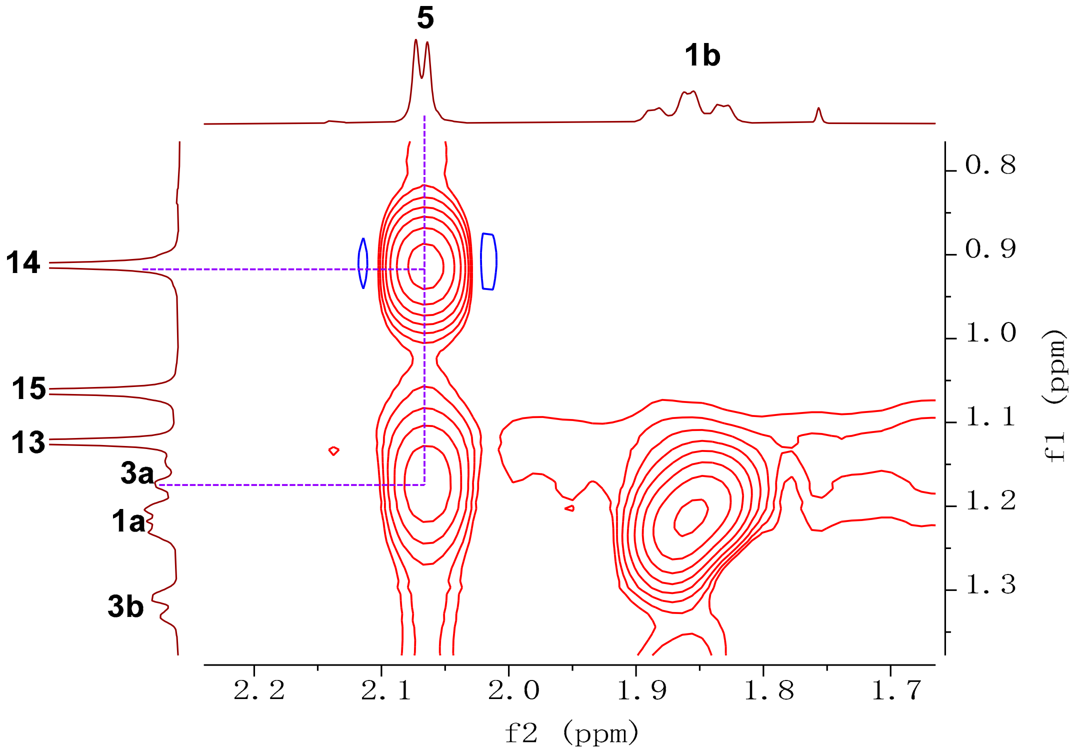


**Figure S41**. The ECD curve of compound **4**

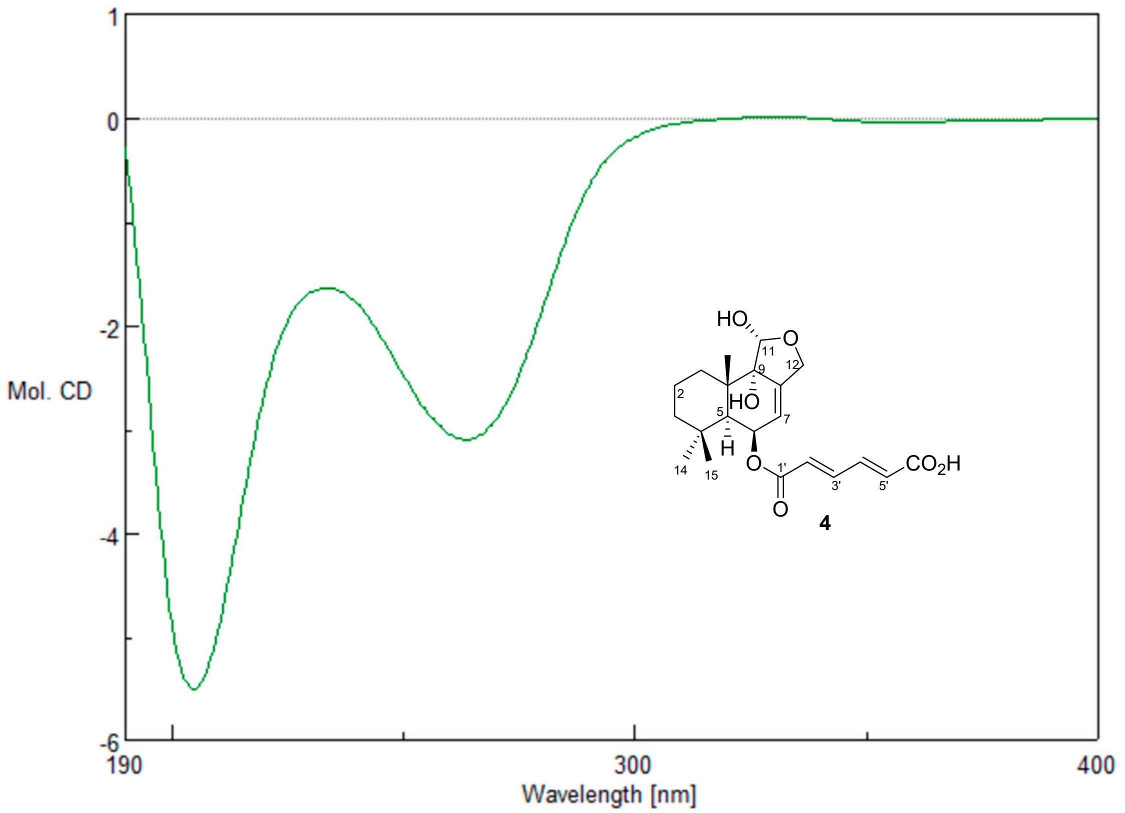

**Figure S42**. The ESIMS spectrum of compound **5**

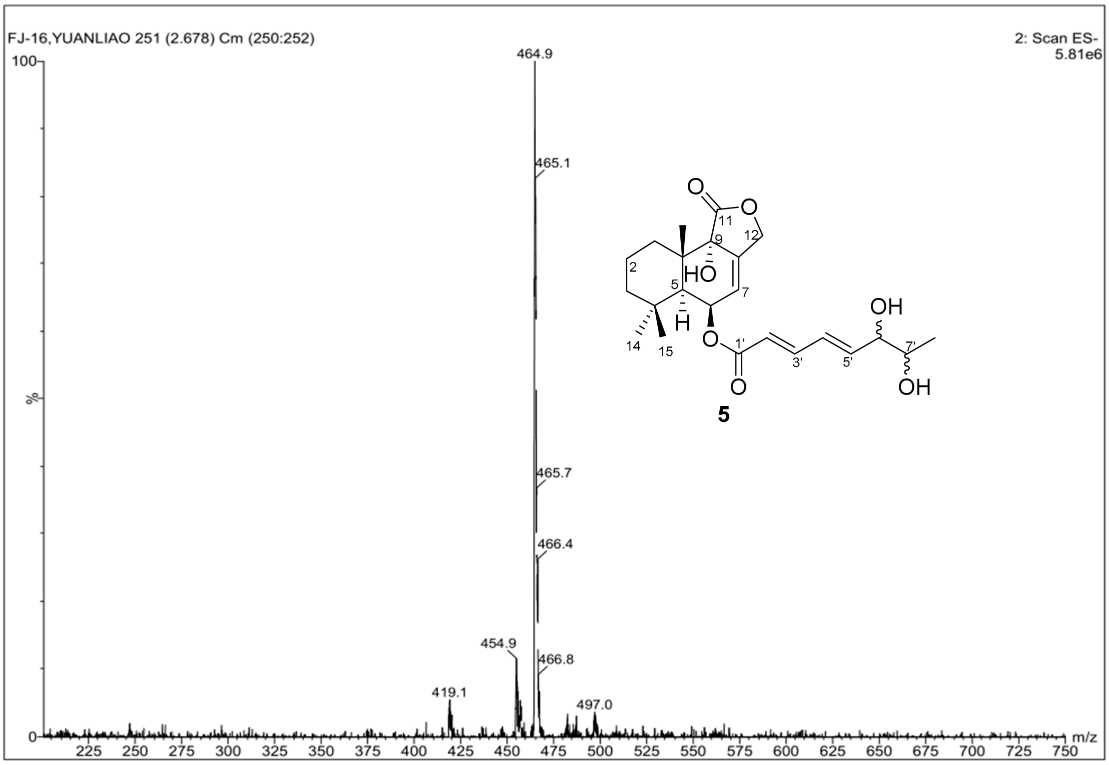


**Figure S43**. The $^{1}$H-NMR spectrum of compound **5** in DMSO-$d_6$

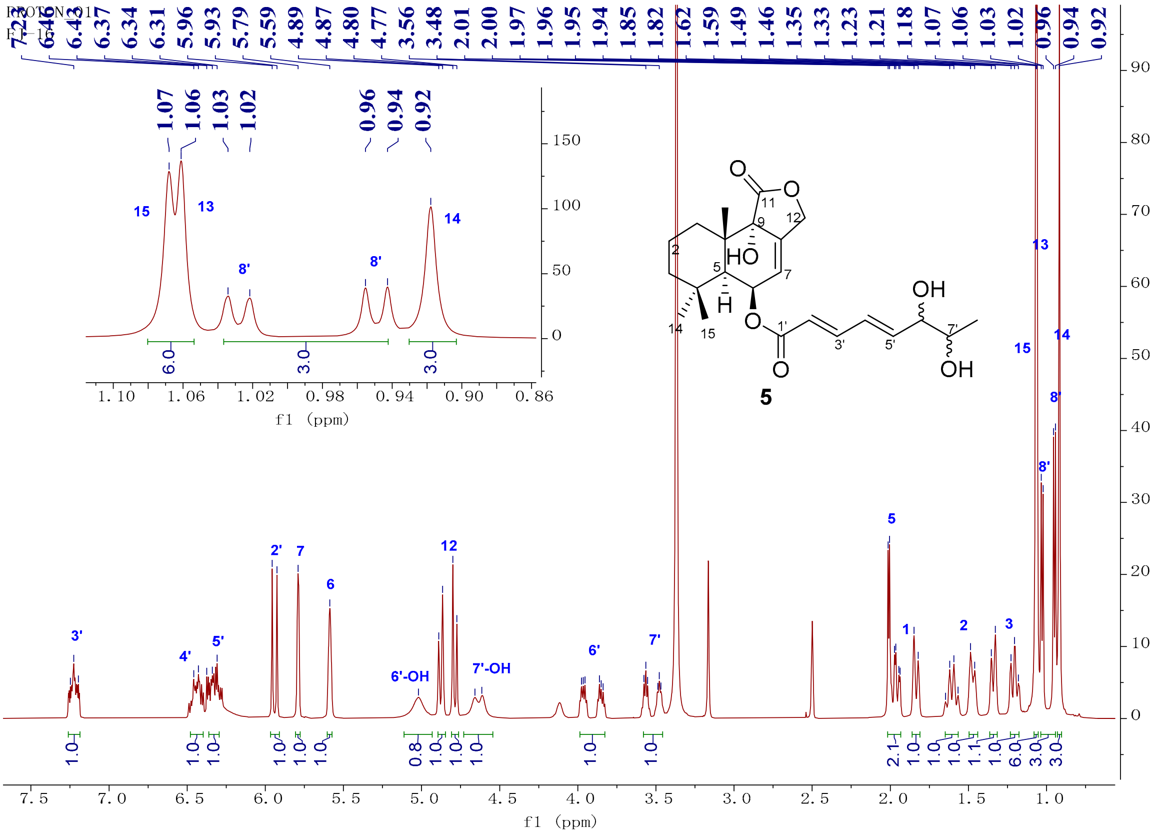

**Figure S44**. The $^{13}$C-NMR spectrum of compound **5** in DMSO-*d*$_6$

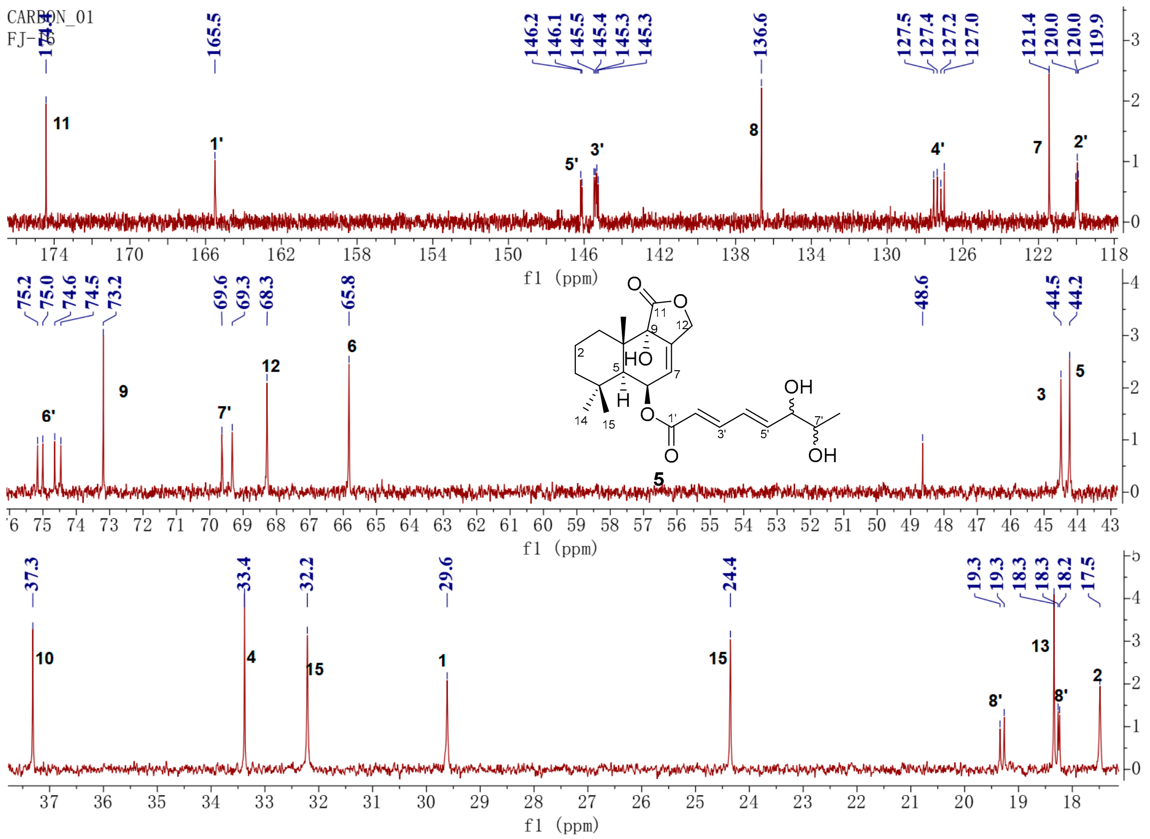


**Figure S45**. The [13]H-NMR spectrum of compound **5a** in DMSO-*d*$_6$

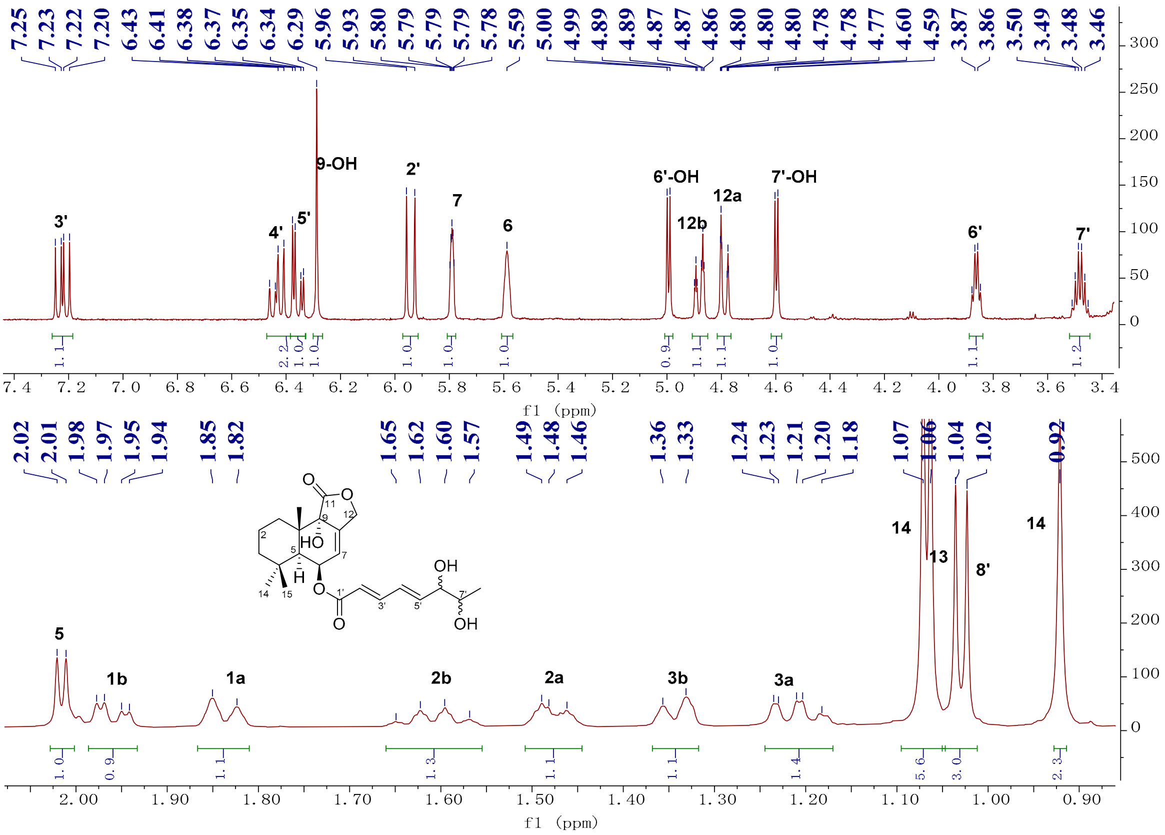

**Figure S46.** The $^{13}$C-NMR spectrum of compound **5a** in DMSO-$d_6$

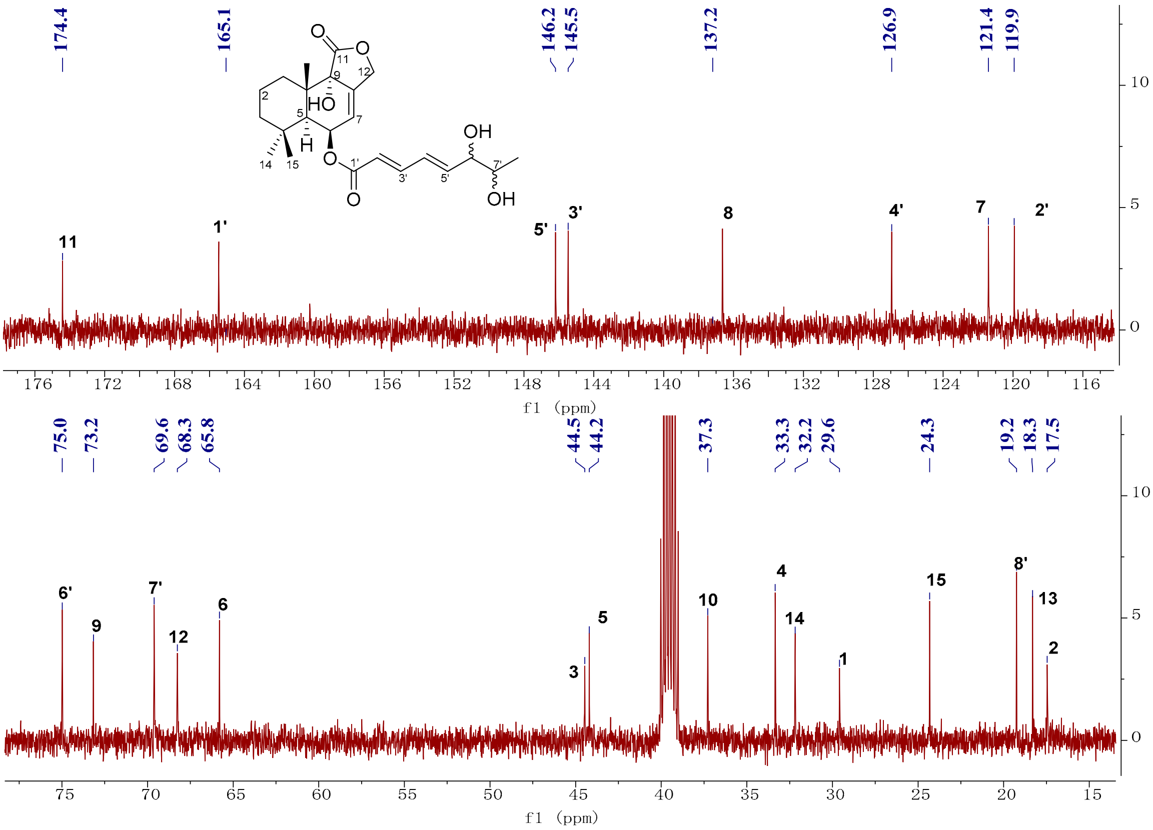


**Figure S47**. The $^{1}$H-$^{1}$H COSY spectrum of compound **5a** in DMSO-$d_6$

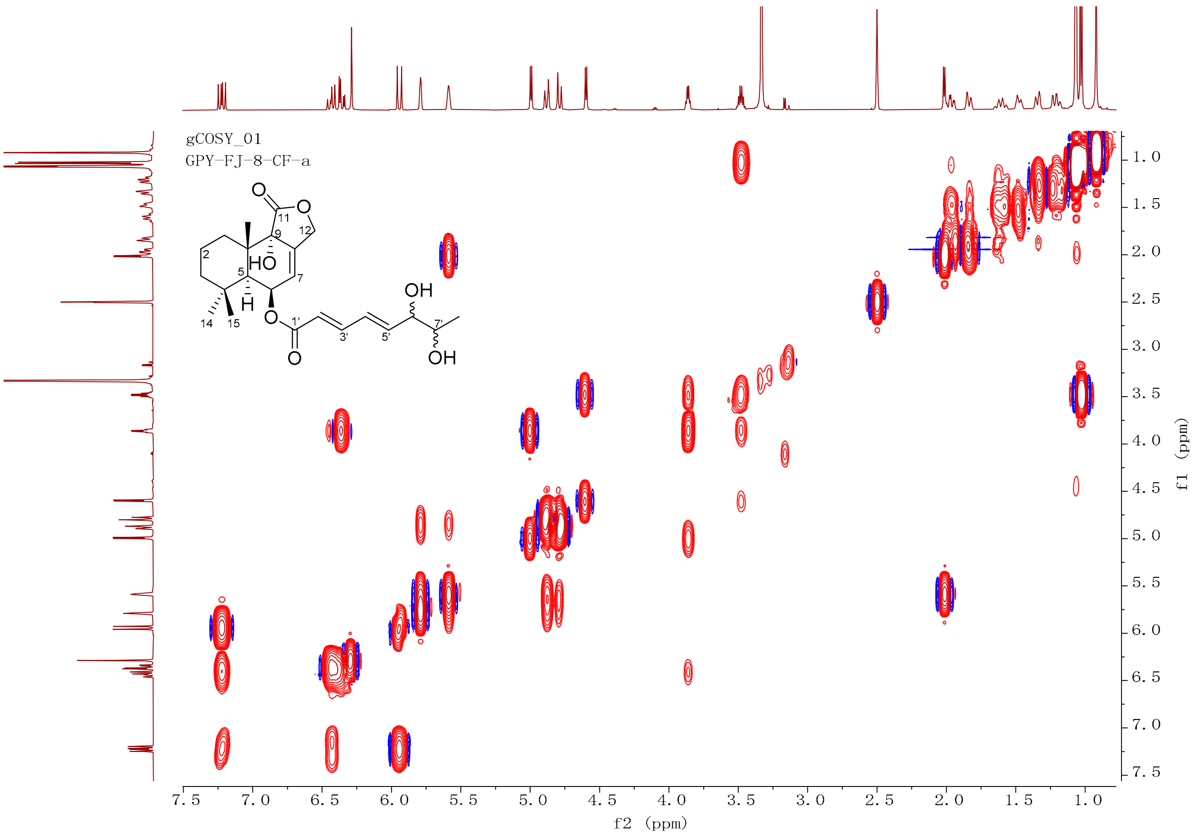

**Figure S48**. The HSQC spectrum of compound **5a** in DMSO-$d_6$

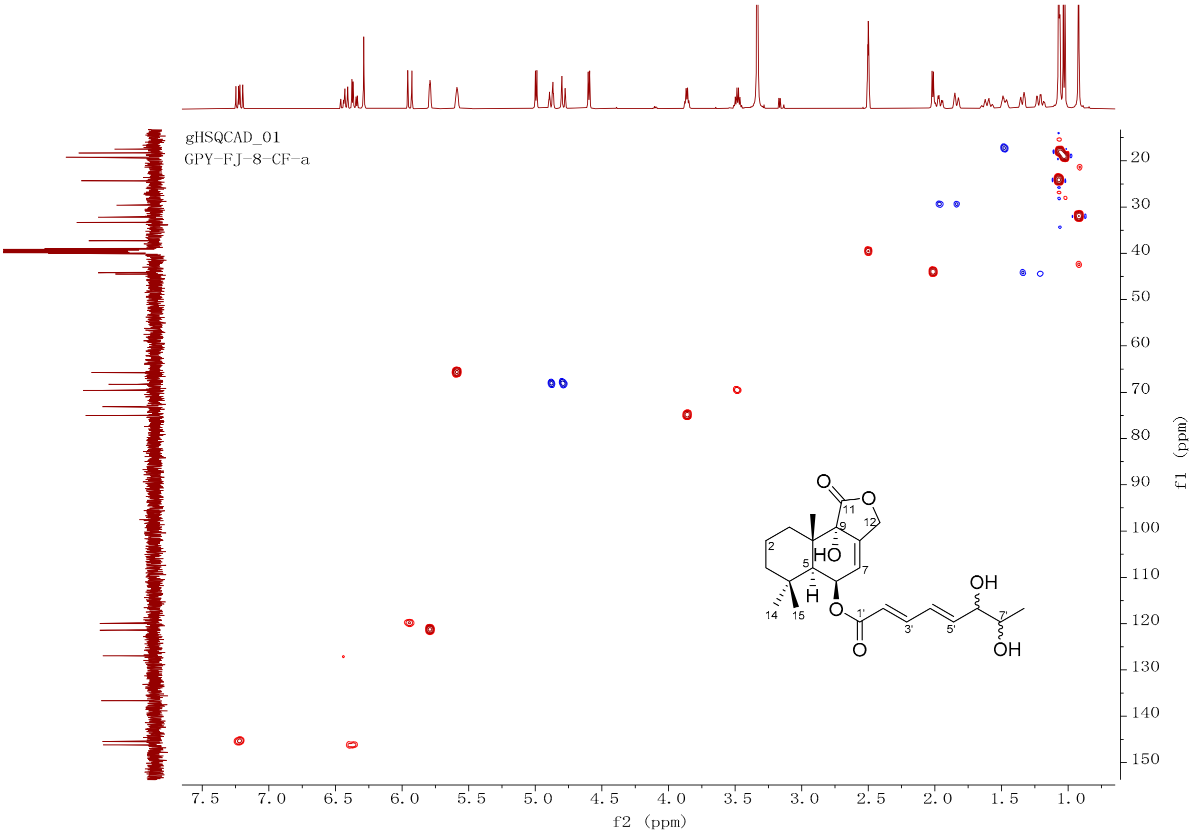


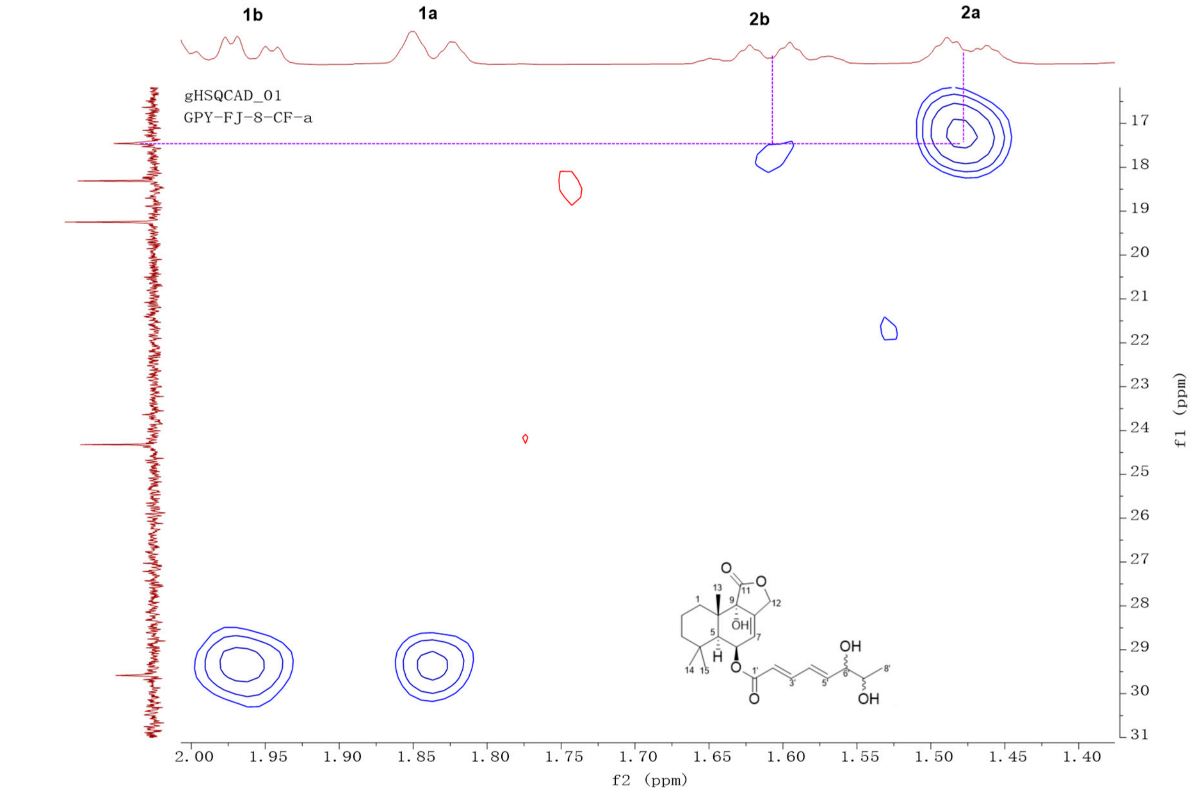

**Figure S49**. The HMBC spectrum of compound **5a** in DMSO-$d_6$

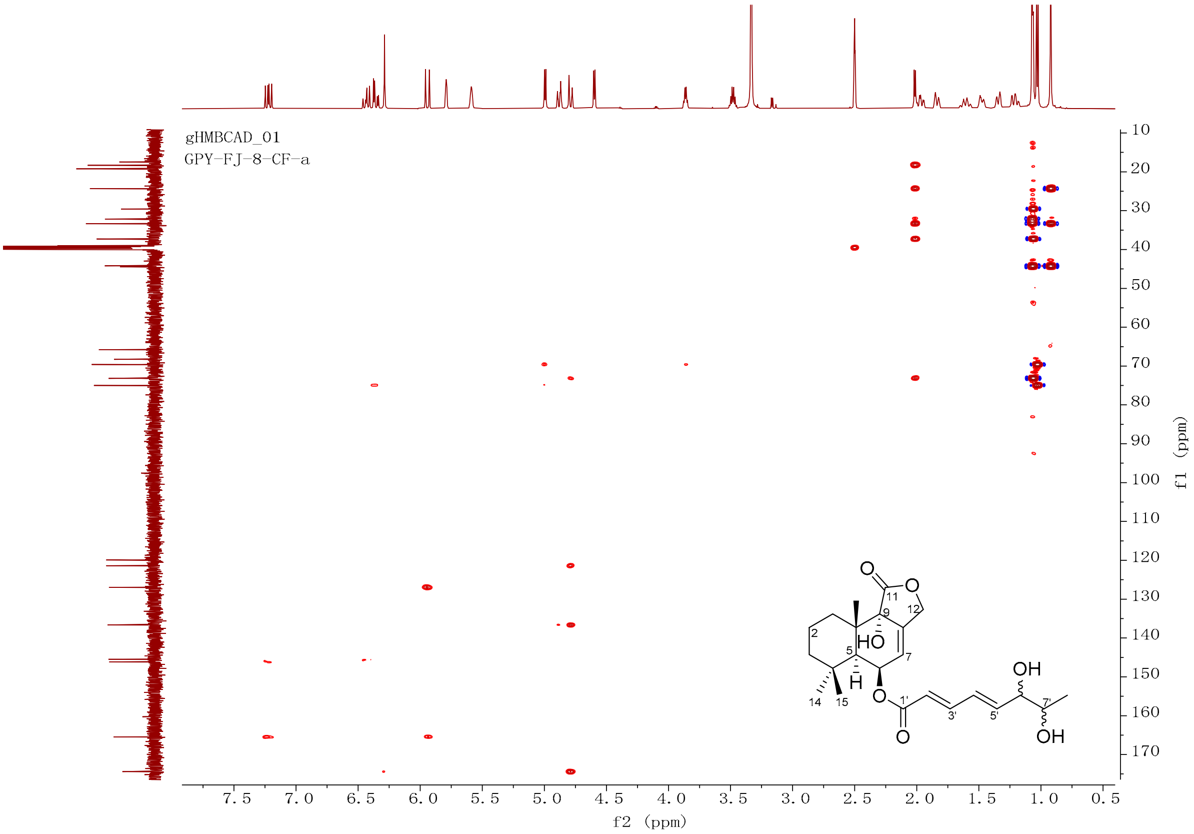

**Figure S50.** The $^1$H-NMR spectrum of compound **5b** in DMSO-$d_6$

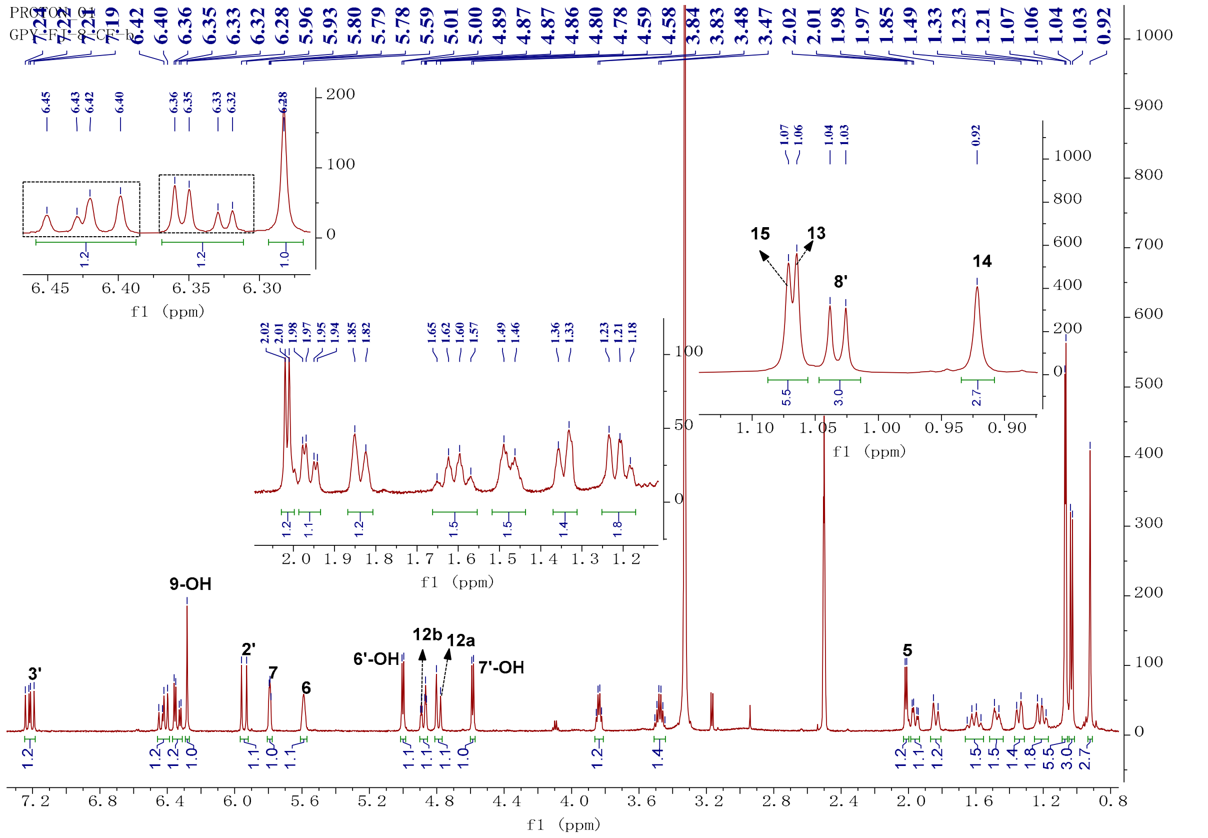

**Figure S51.** The $^{13}$C-NMR spectrum of compound **5b** in DMSO-$d_6$

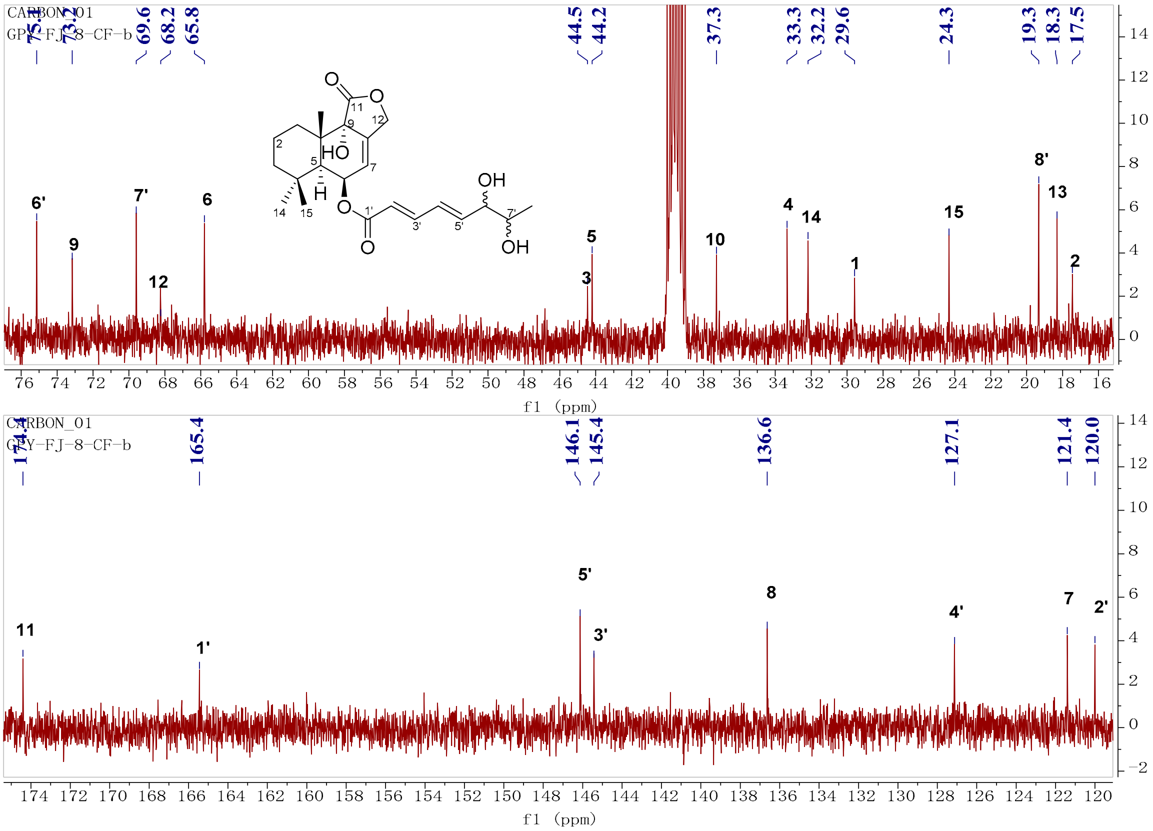

**Figure S52.** The $^{1}$H-NMR spectrum of compound **5c** in DMSO-$d_6$

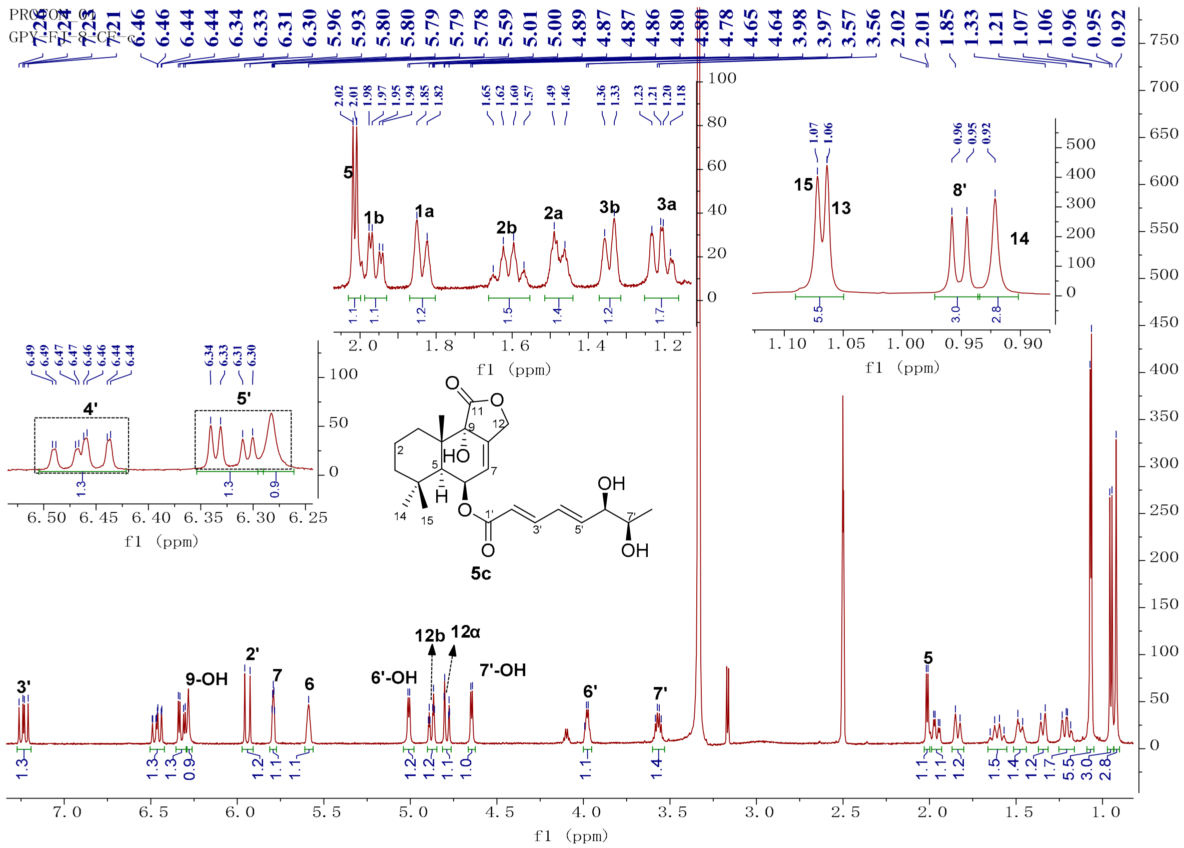

**Figure S53.** The $^{13}$C-NMR spectrum of compound **5c** in DMSO-$d_6$

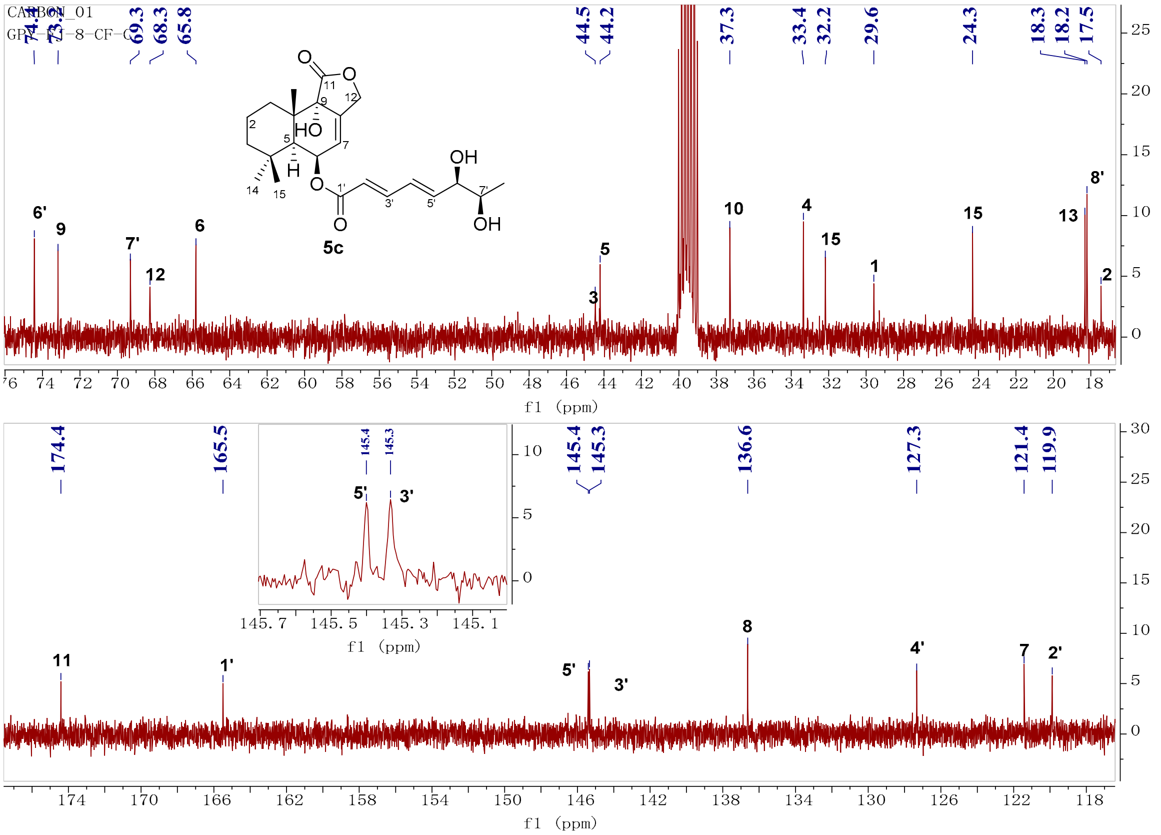


**Figure S54**. The $^{1}$H-$^{1}$H COSY spectrum of compound **5c** in DMSO-$d_6$

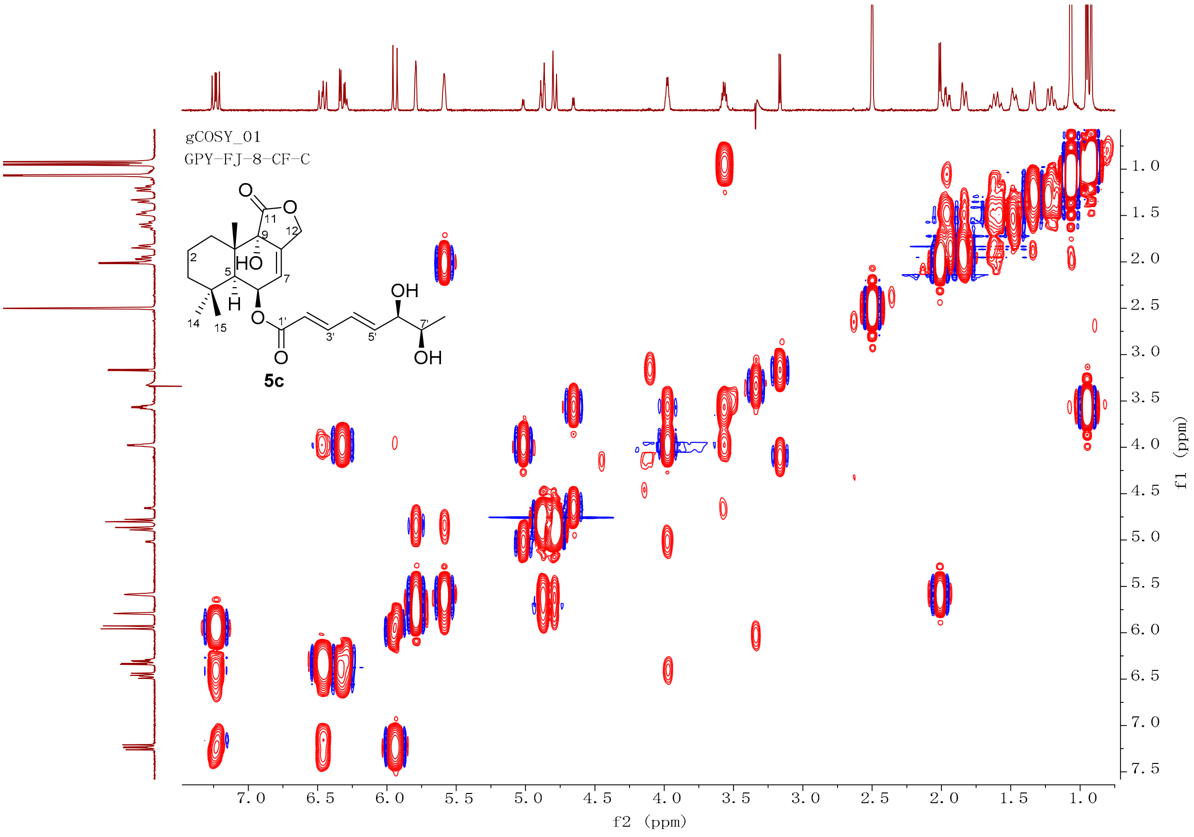

**Figure S55**. The HSQC spectrum of compound **5c** in DMSO-$d_6$

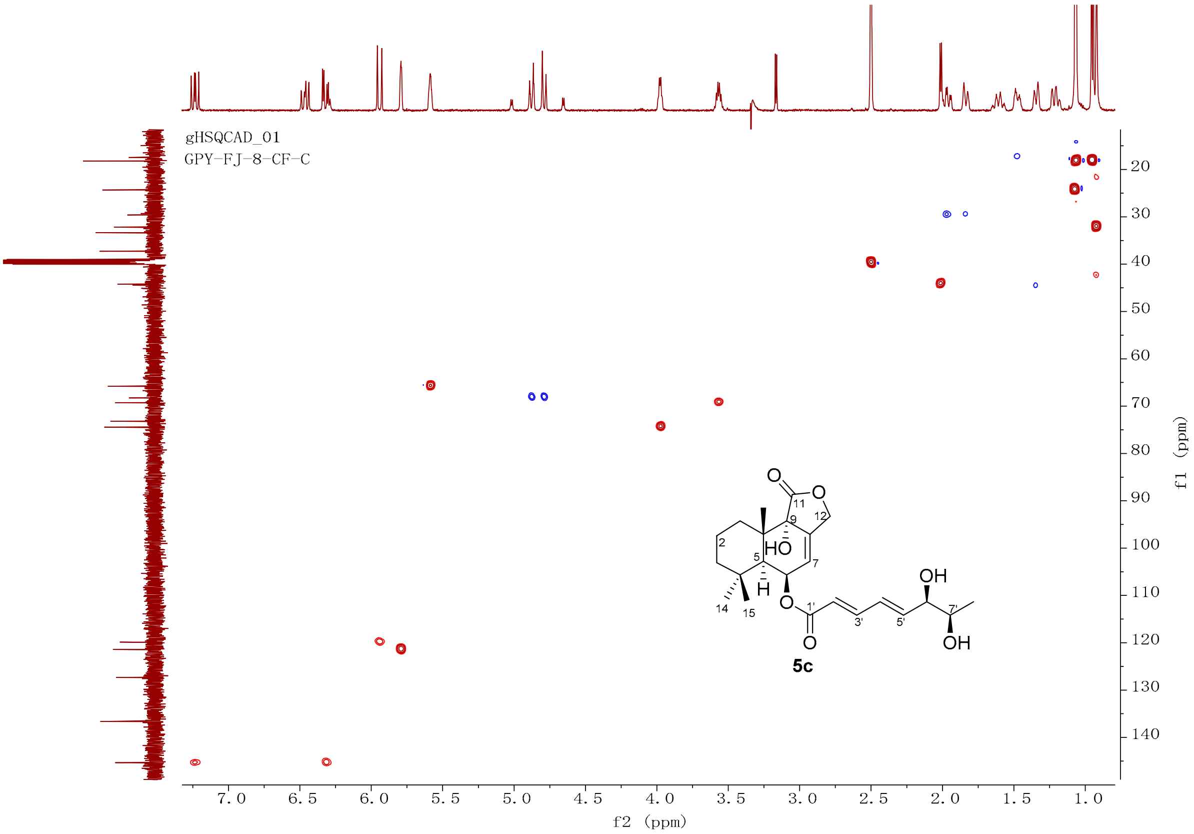


**Figure S56**. The HMBC spectrum of compound **5c** in DMSO-$d_6$

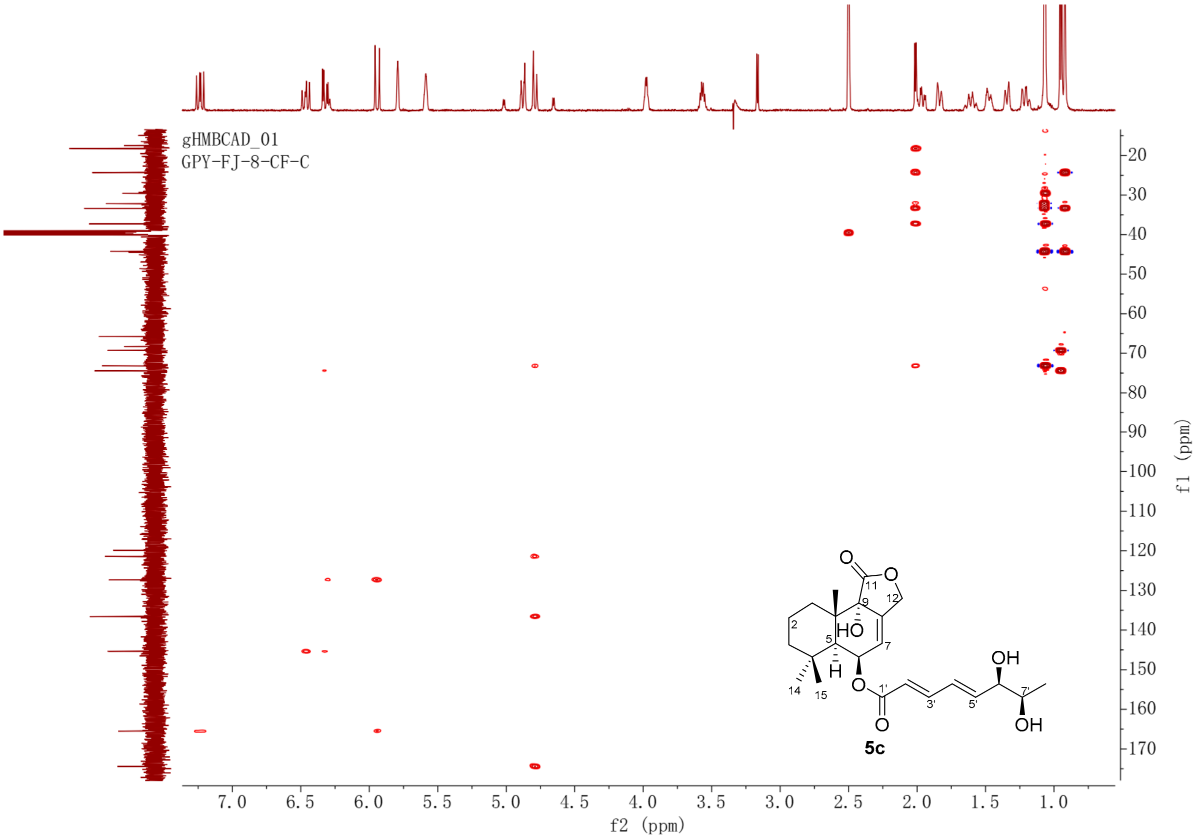

**Figure S57**. The $^{1}$H-NMR spectrum of compound **5d** in DMSO-$d_6$

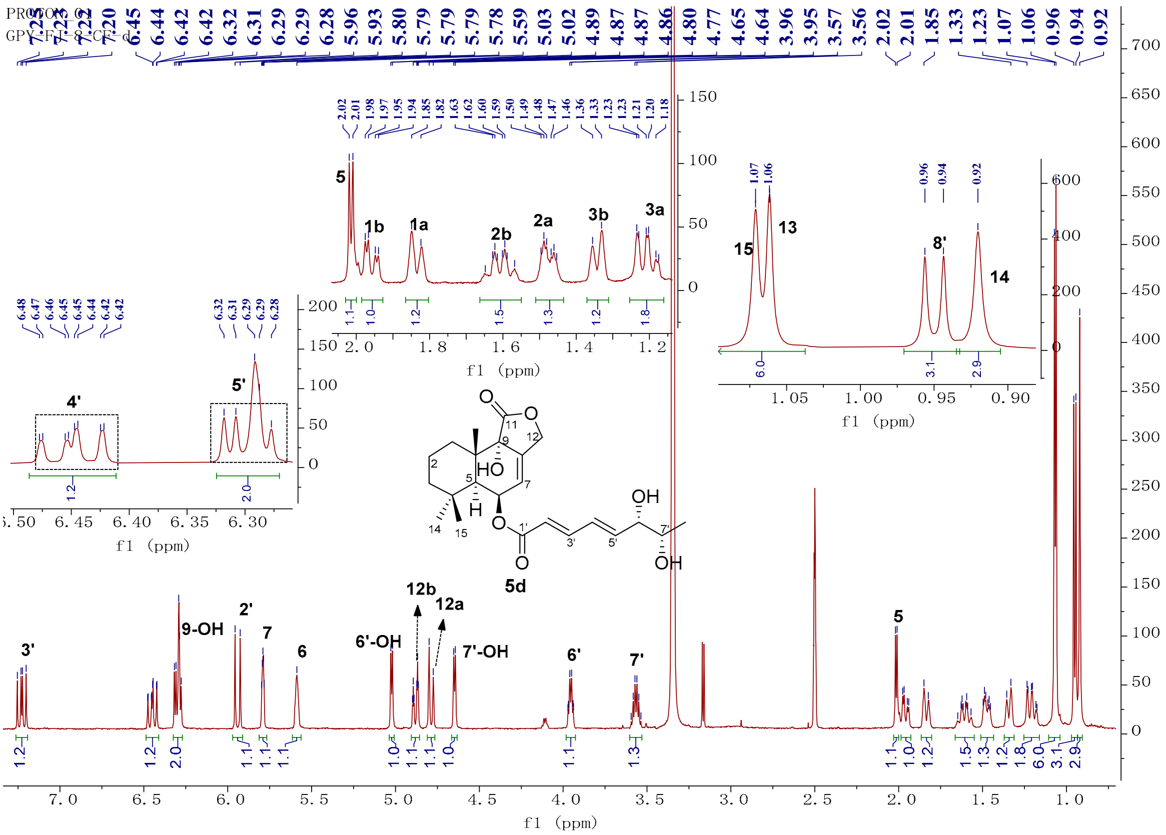


**Figure S58.** The $^{13}$C-NMR spectrum of compound **5d** in DMSO-$d_6$

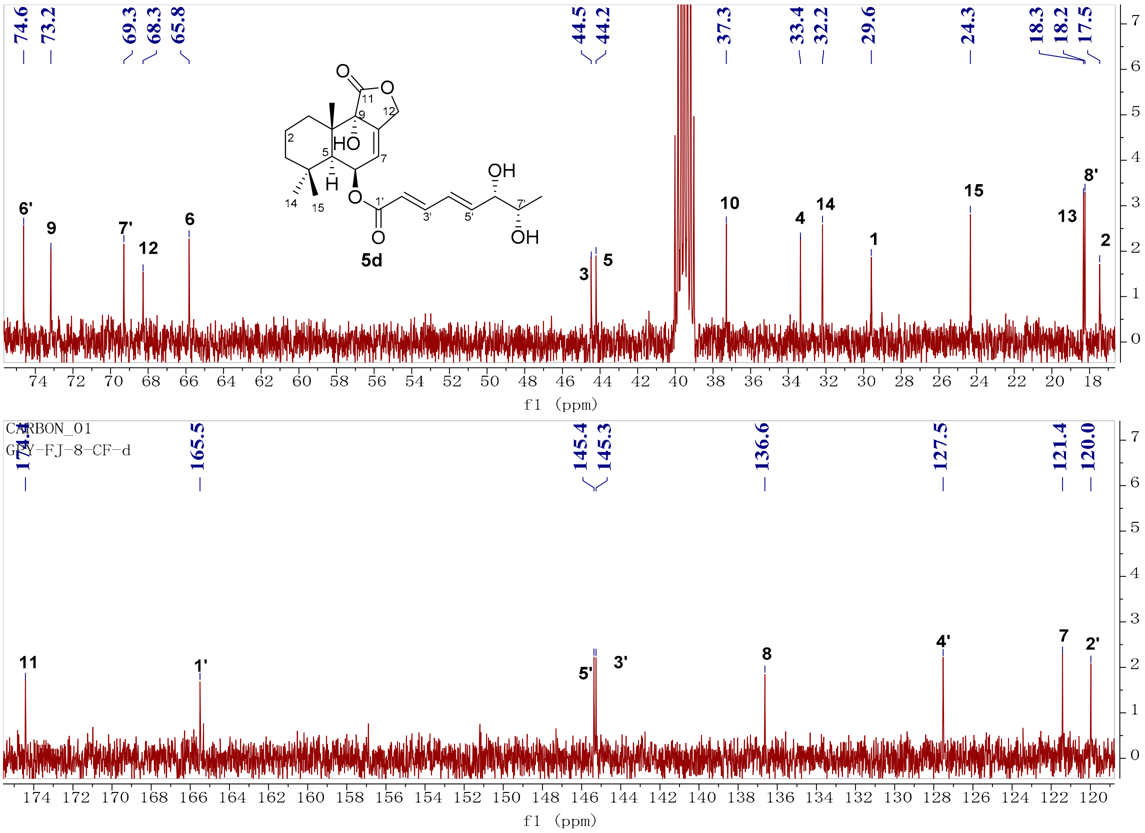

**Figure S59.** The [1H]C-NMR difference of compound **5a**, **5b**, **5c** and **5d** between $\delta_H$ 3.0-6.5

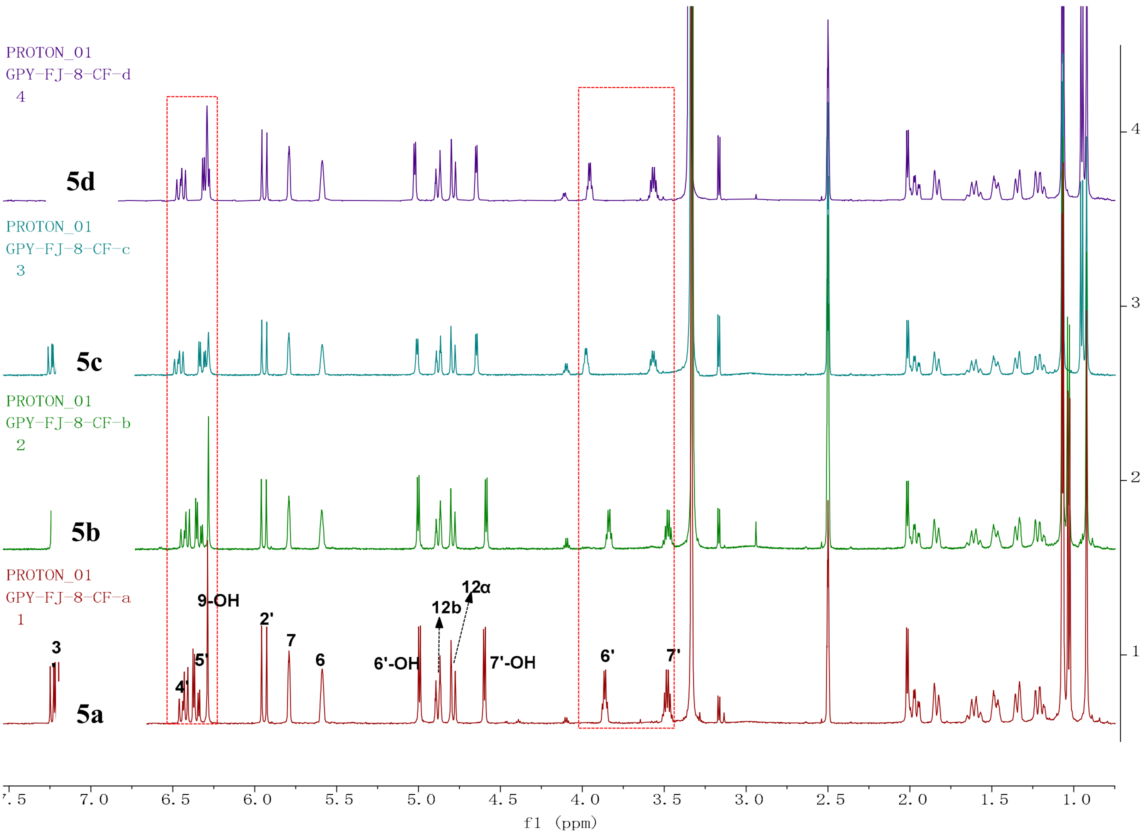


**Figure S60**. $^{13}$C-NMR difference of **5a**, **5b**, **5c** and **5d** between methyl carbon (C-8')

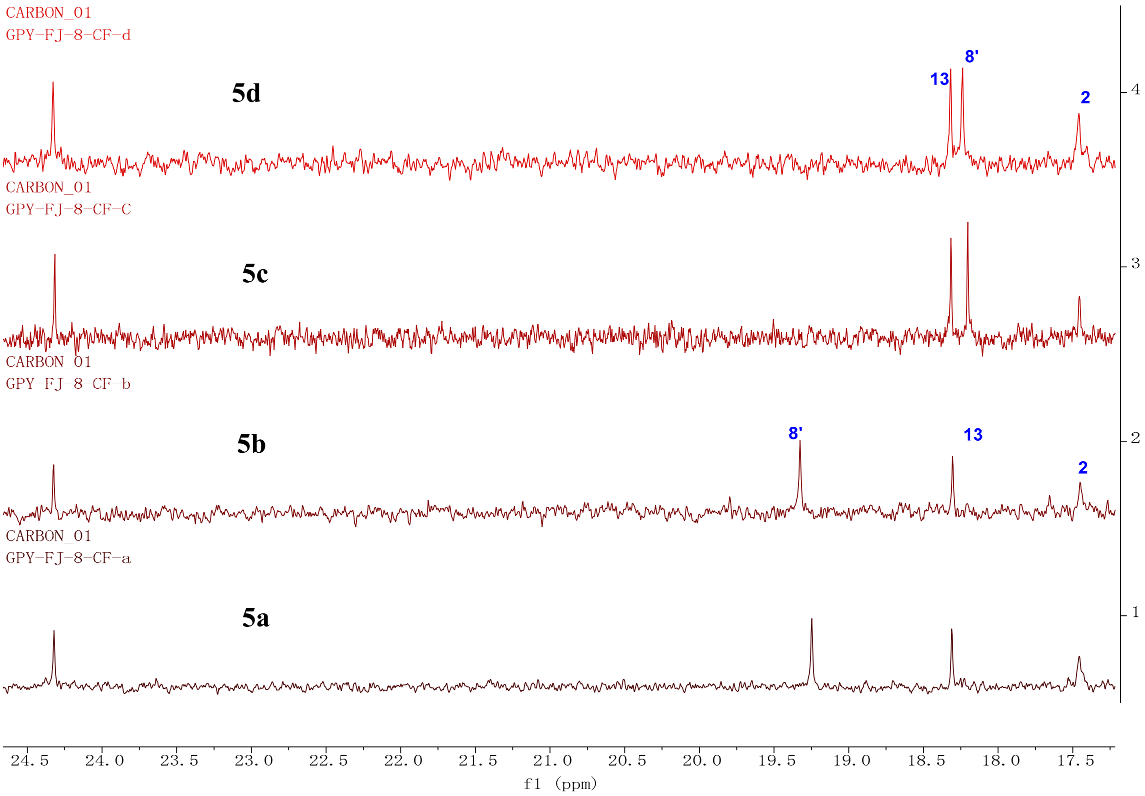

**Figure S61**. The ECD curve of compound **5a**

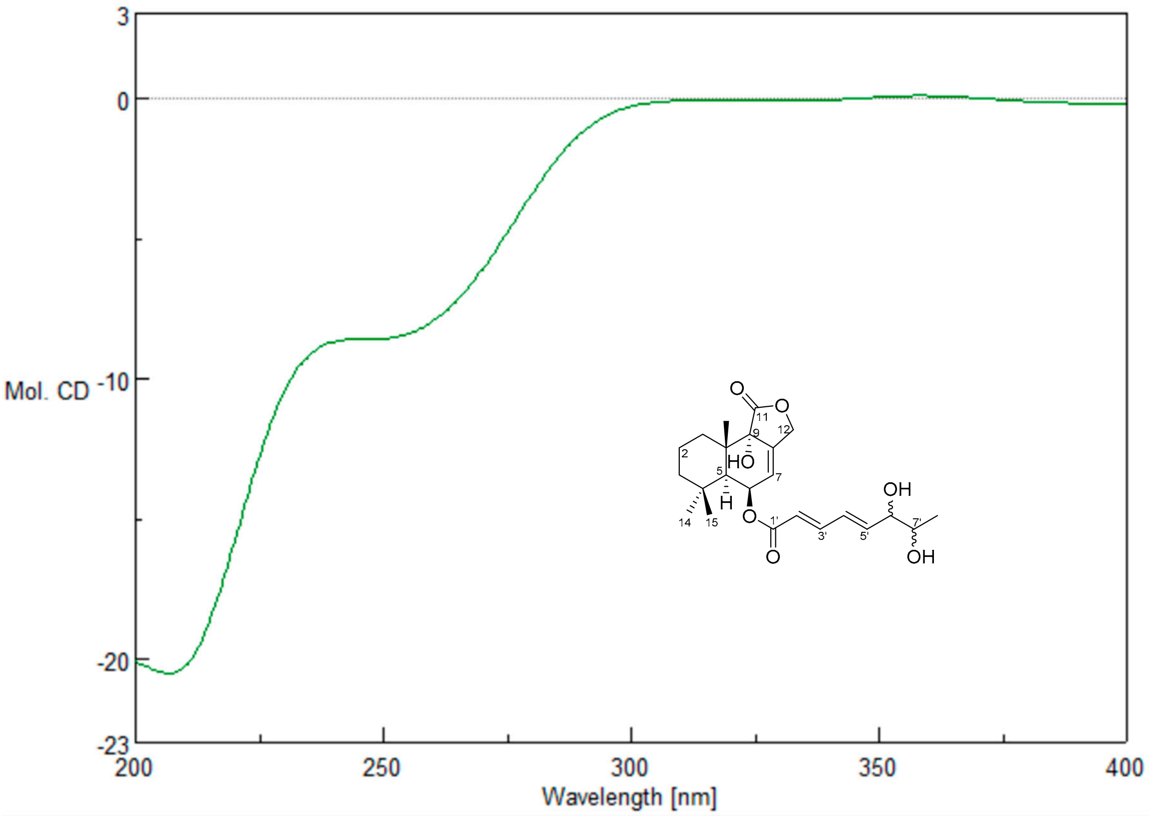


**Figure S62**. The ECD curve of compound **5b**

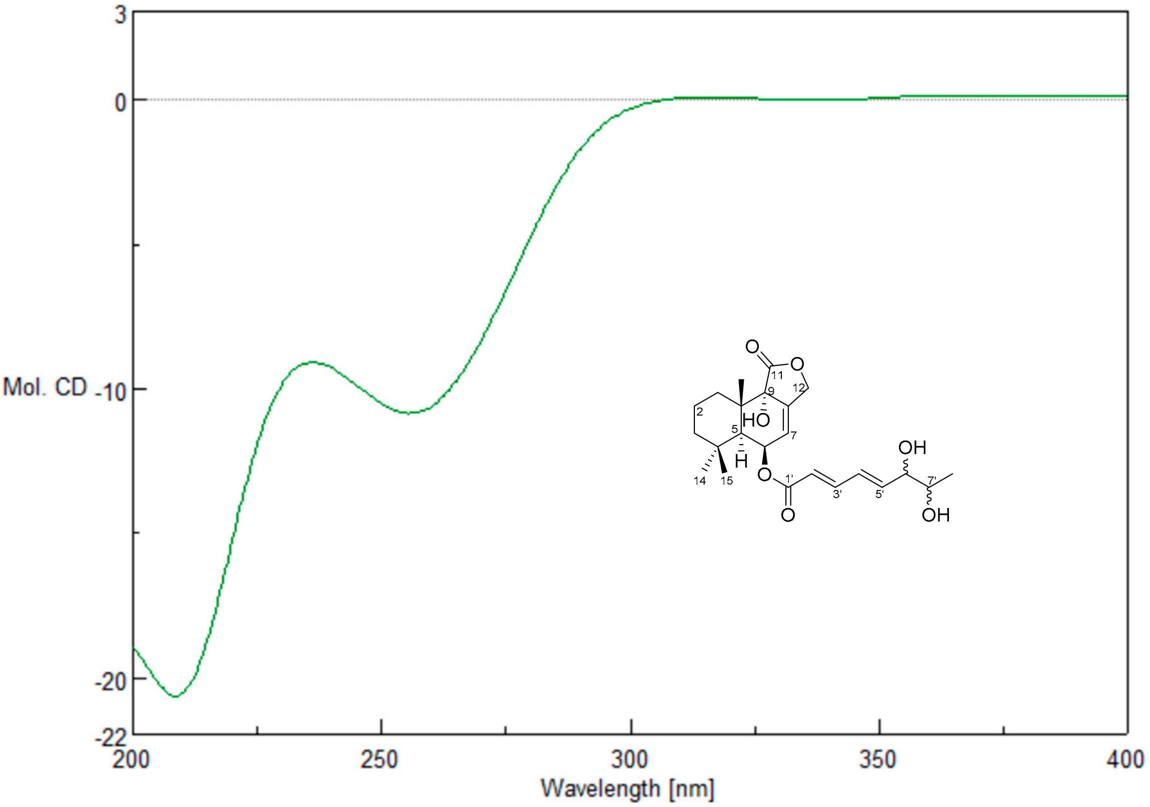

**Figure S63**. The ECD curve of compound **5c**

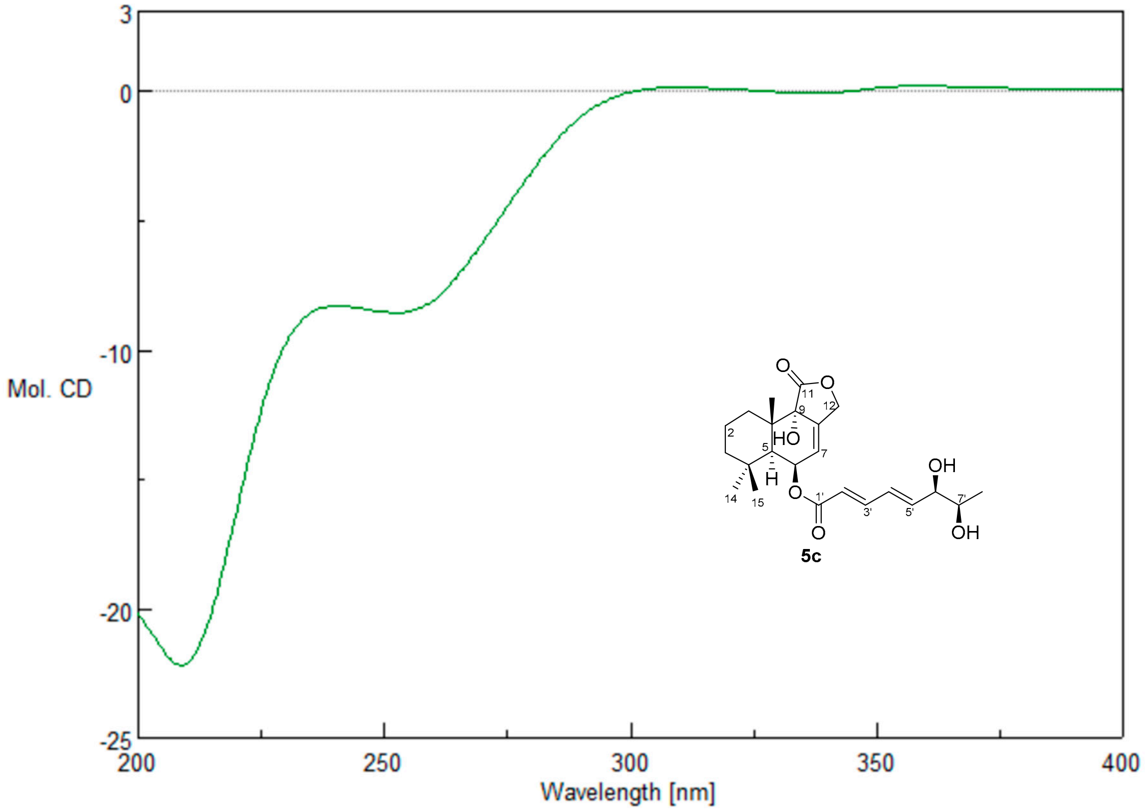


**Figure S64**. The ECD curve of compound **5d**

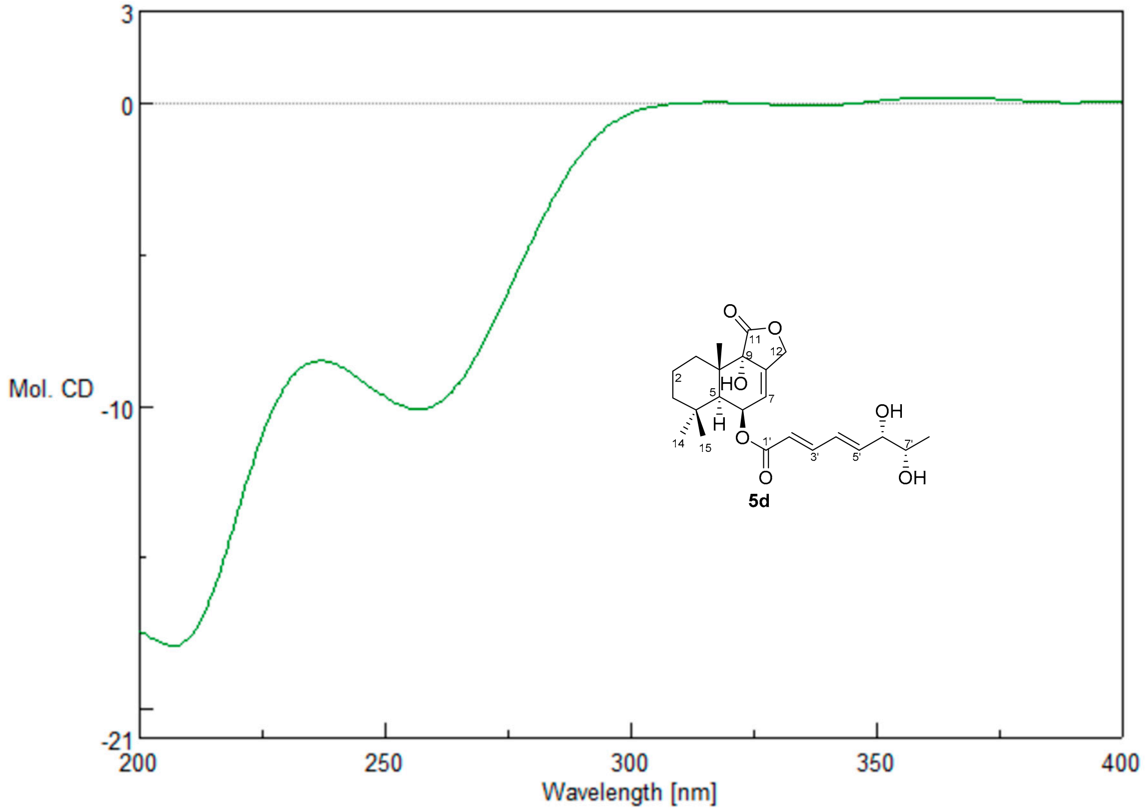

**Figure S65**. The ESIMS spectrum of compound **5e**

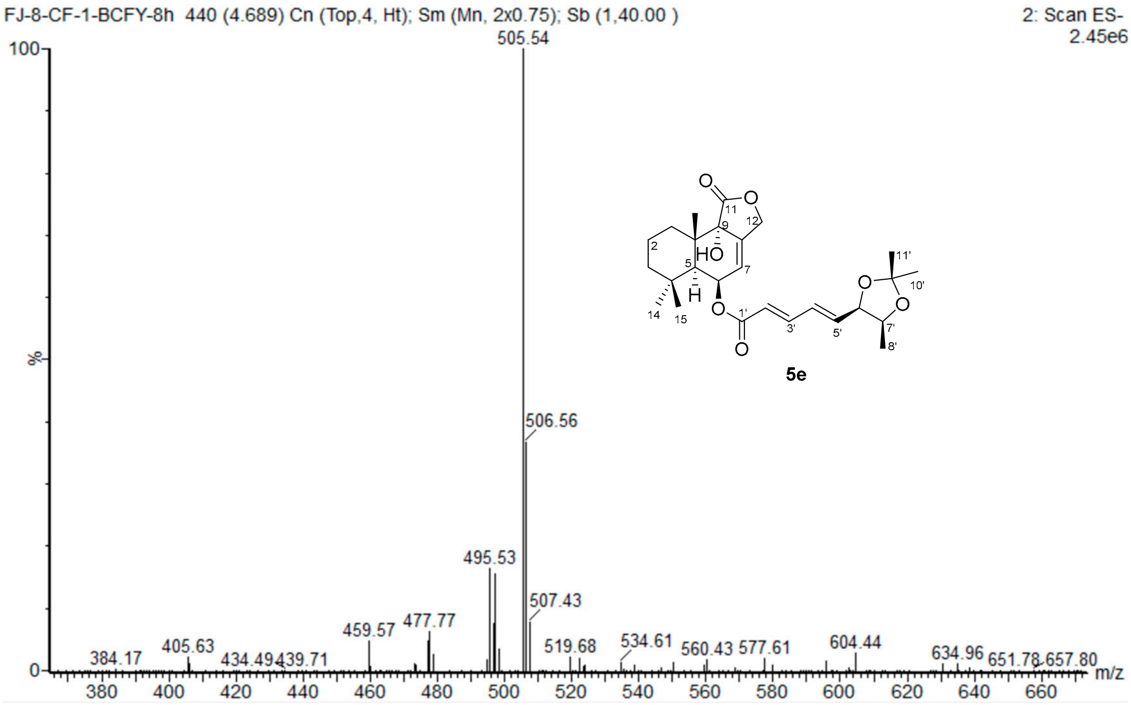

**Figure S66.** The $^{1}$H-NMR spectrum of compound **5e** in DMSO-$d_6$

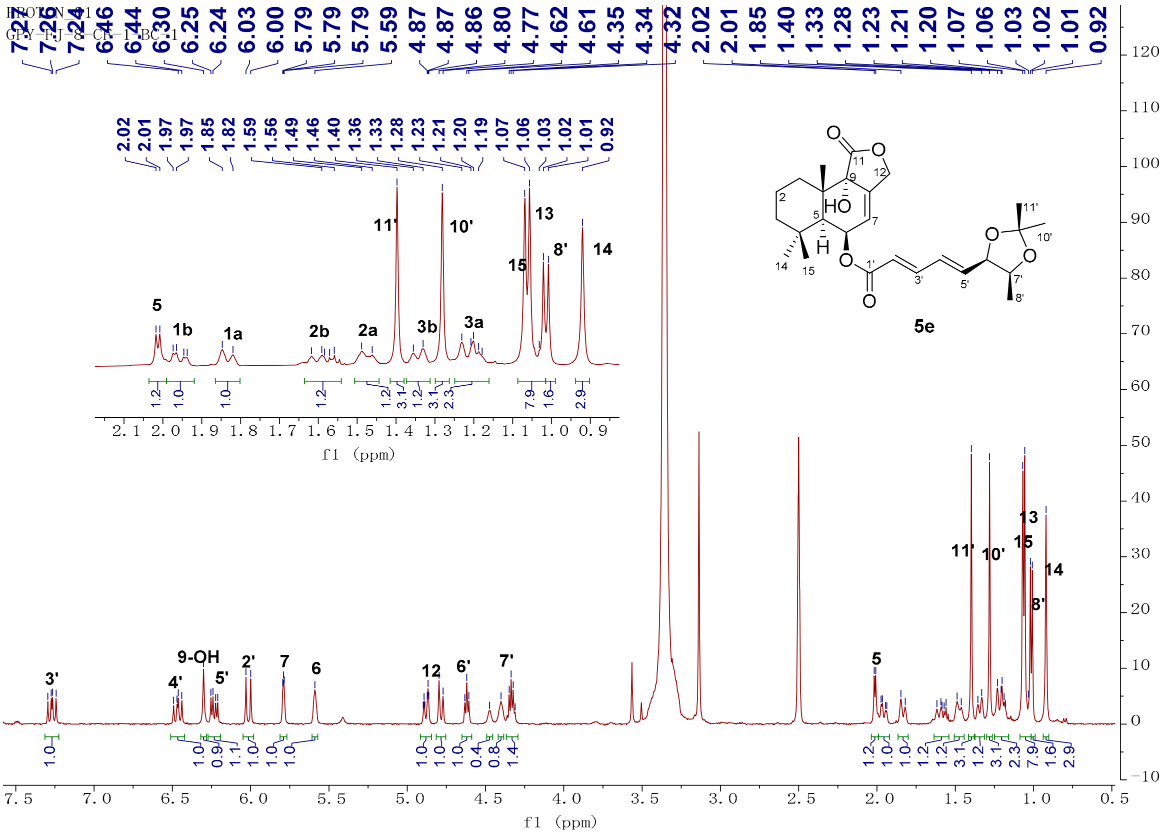

**Figure S67.** The $^{13}$C-NMR spectrum of compound **5e** in DMSO-$d_6$

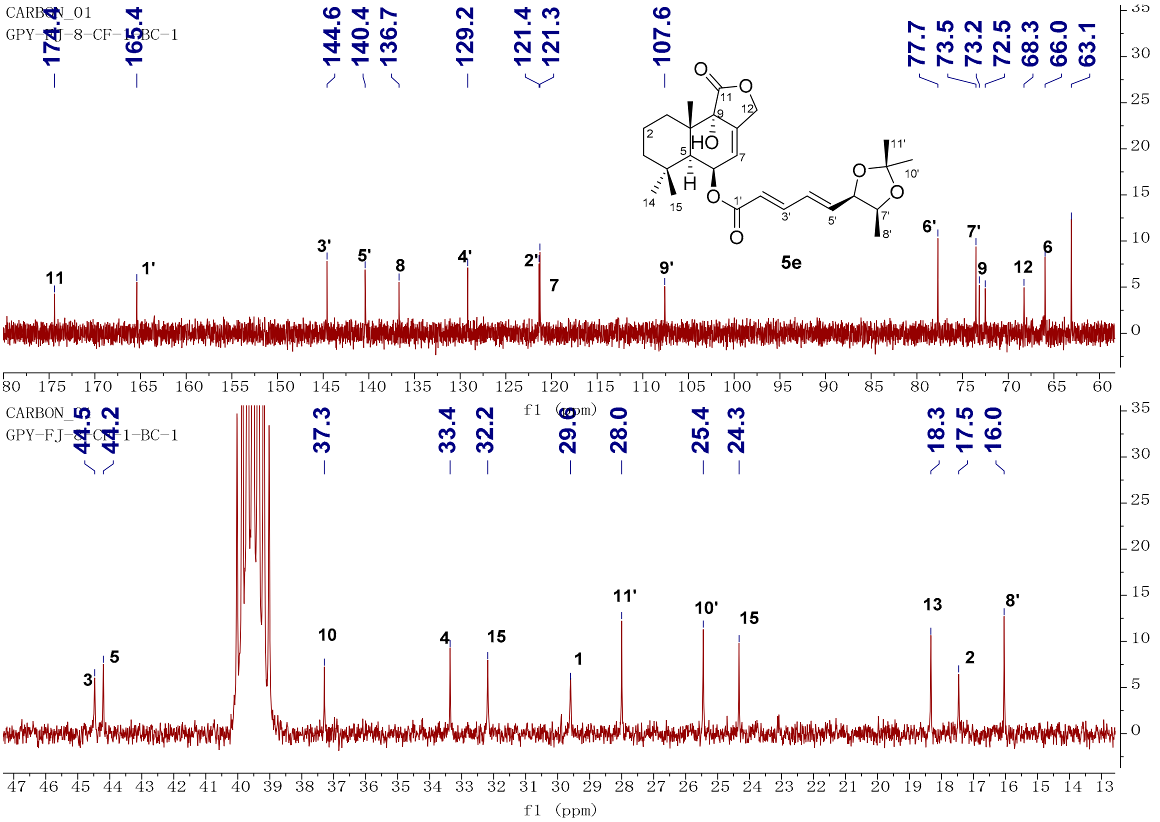


**Figure S68**. The $^{1}$H-$^{1}$H COSY spectrum of compound **5e** in DMSO-$d_6$

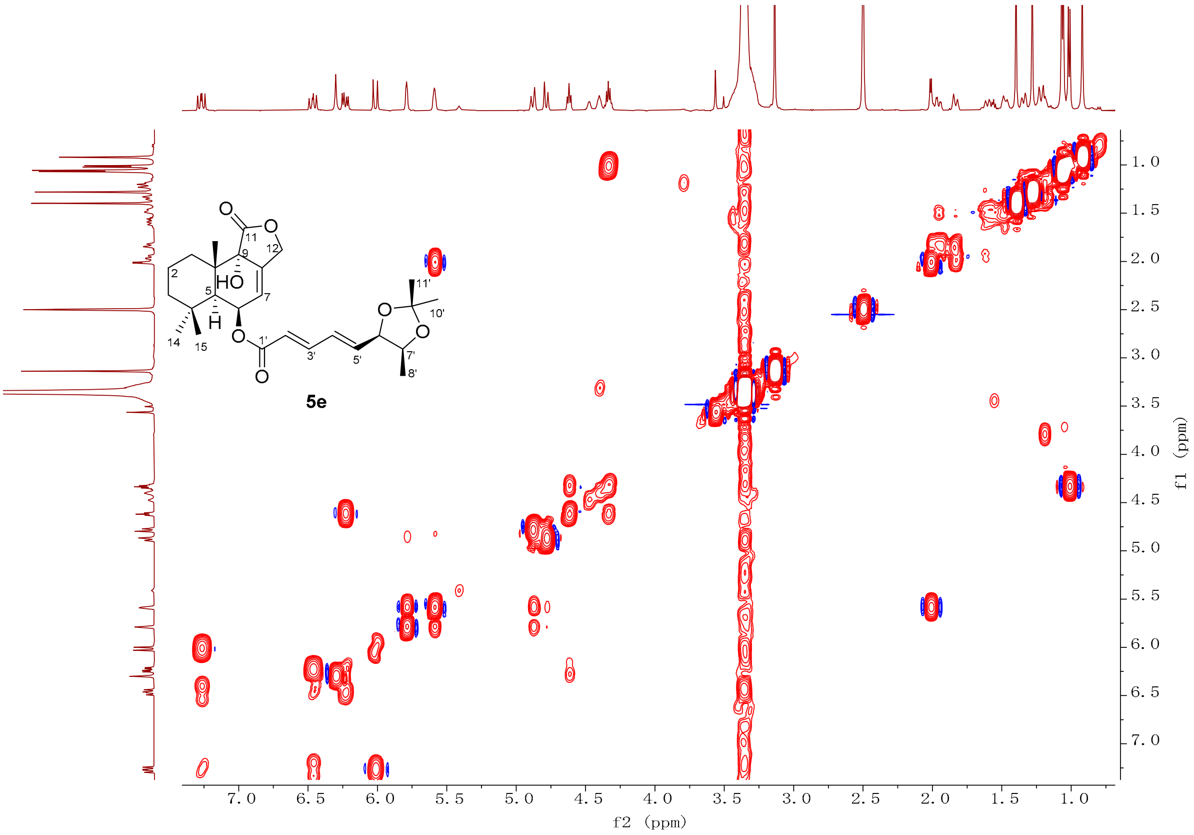

**Figure S69**. The HSQC spectrum of compound **5e** in DMSO-$d_6$

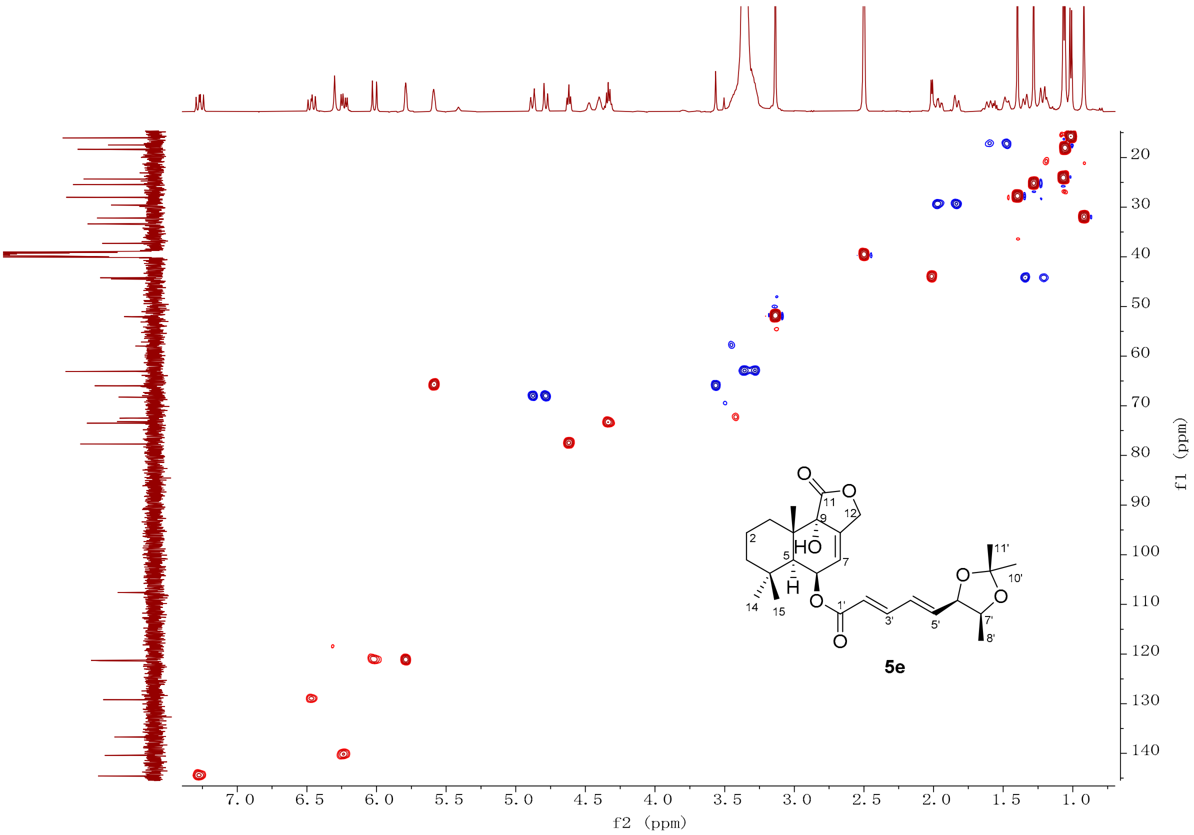


**Figure S70**. The HMBC spectrum of compound **5e** in DMSO-$d_6$

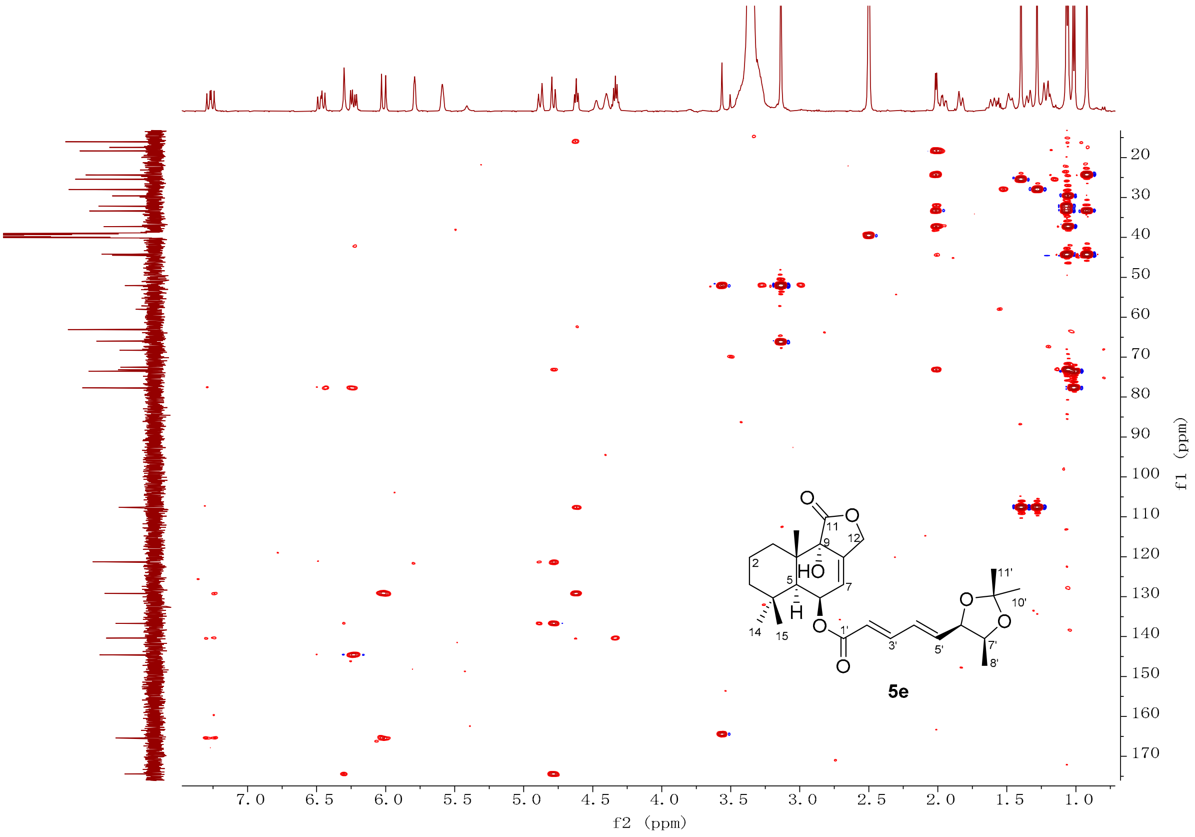

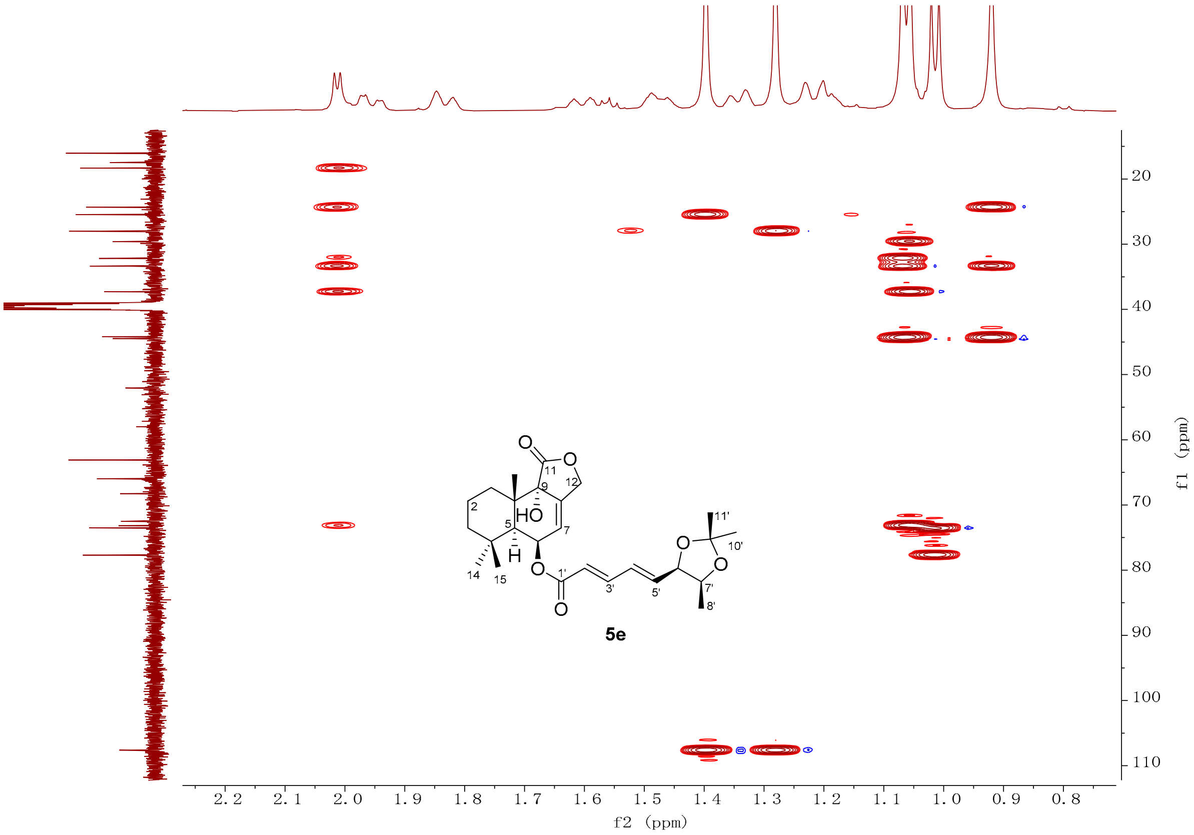


**Figure S71**. The NOESY spectrum of compound **5e** in DMSO-$d_6$

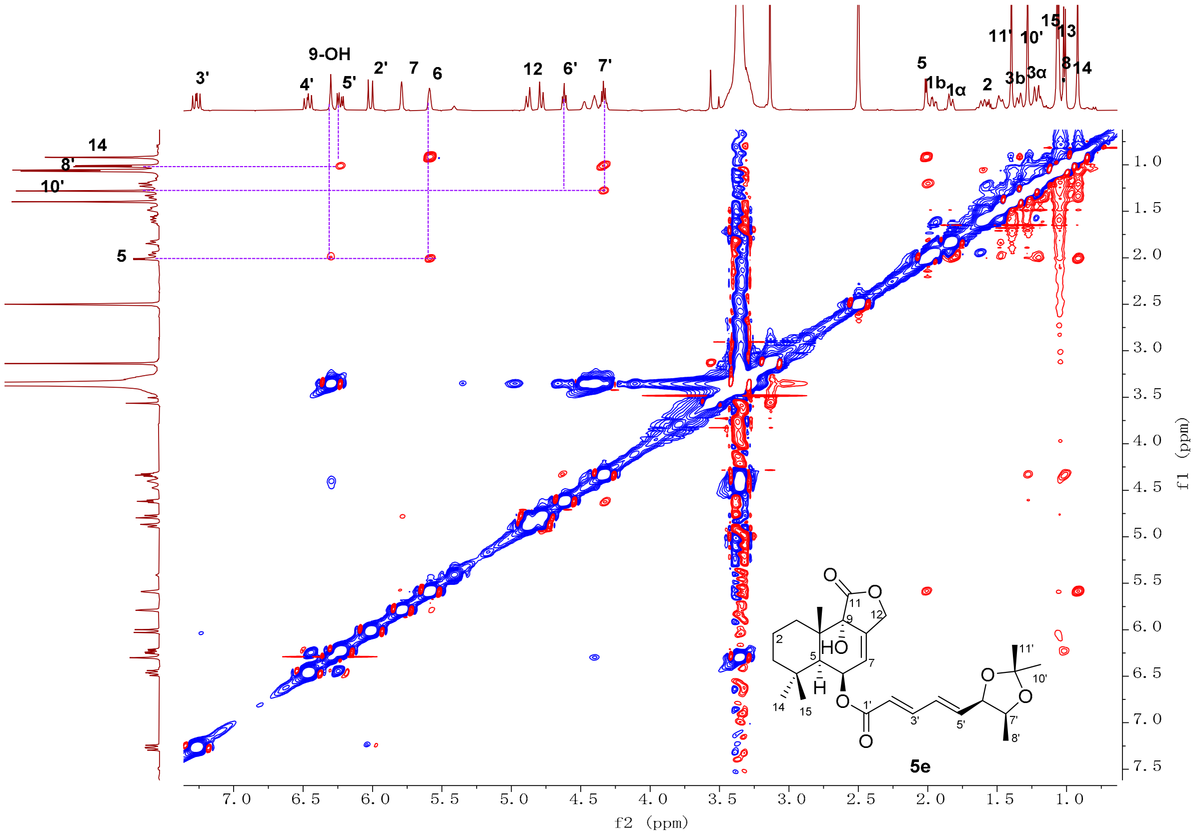

**Figure S72**. The ECD curve of compound **5e**

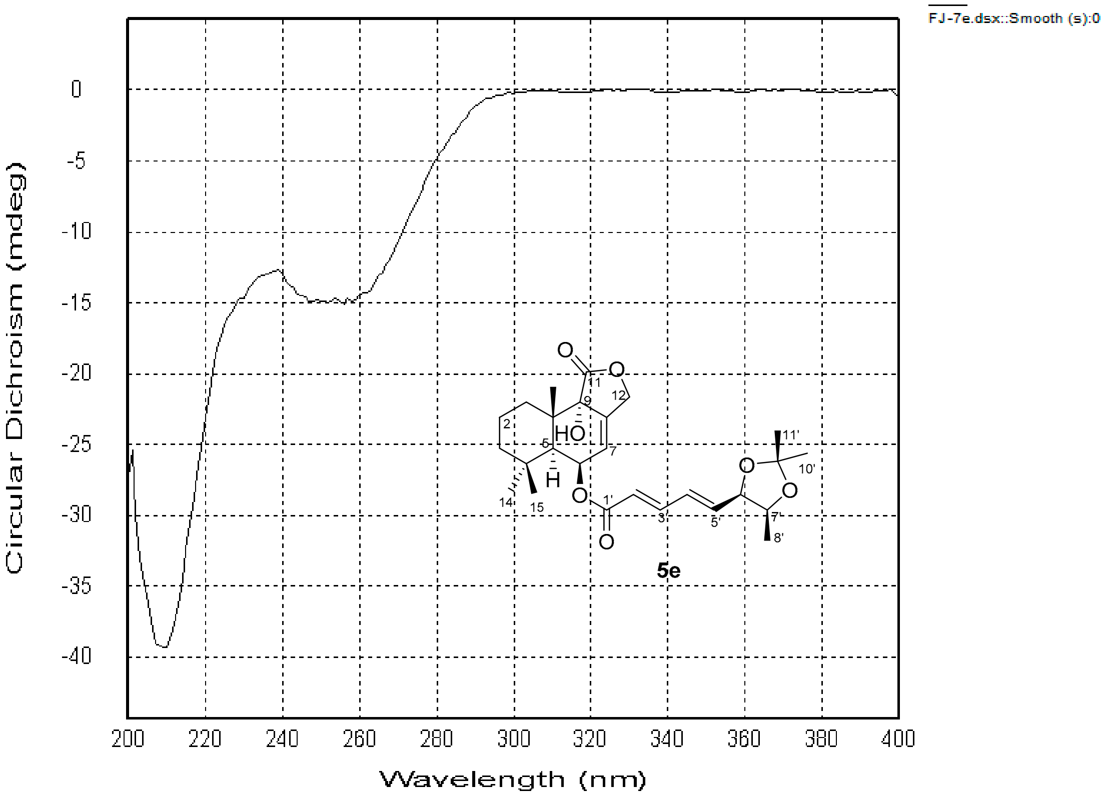


**Figure S73**. ECD curve for the complex of **5c** with $Mo_2(OAc)_4$ subtracted from the inherent ECD

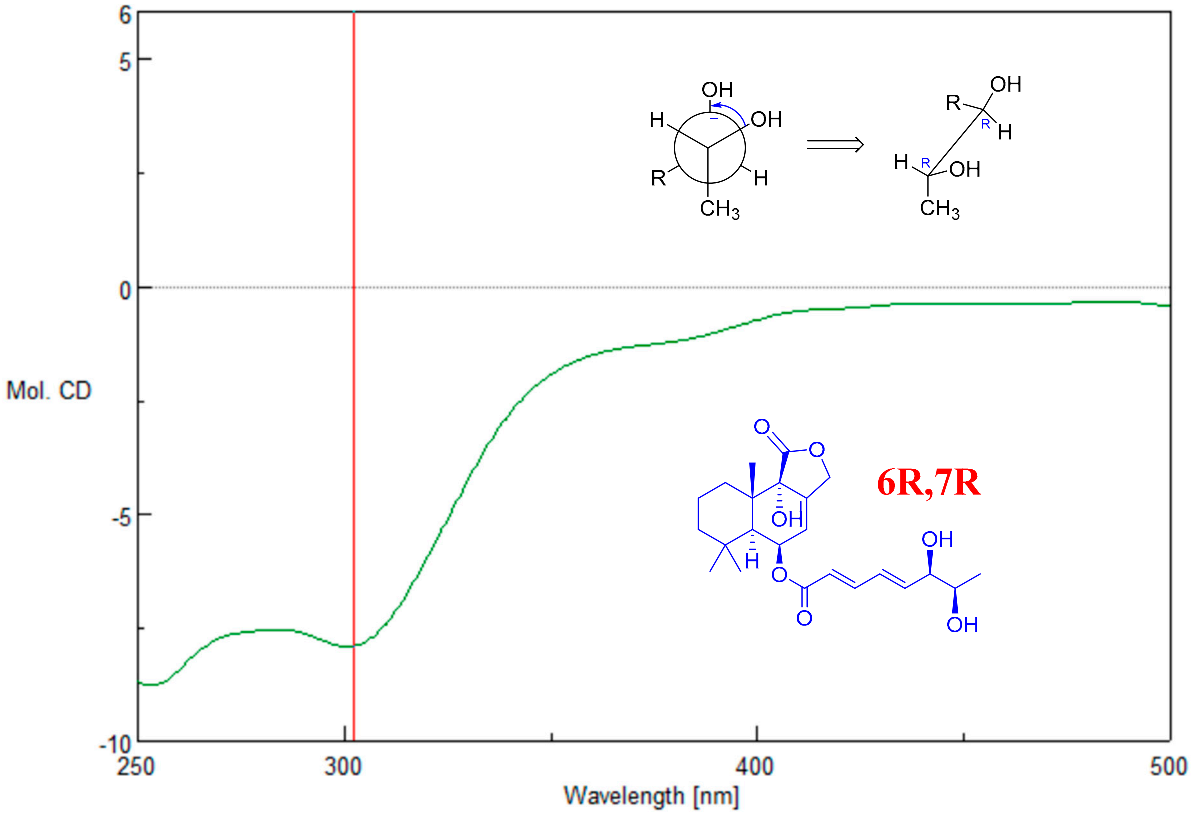

**Figure S74**. ECD curve for the complex of **5d** with $Mo_2(OAc)_4$ subtracted from the inherent ECD

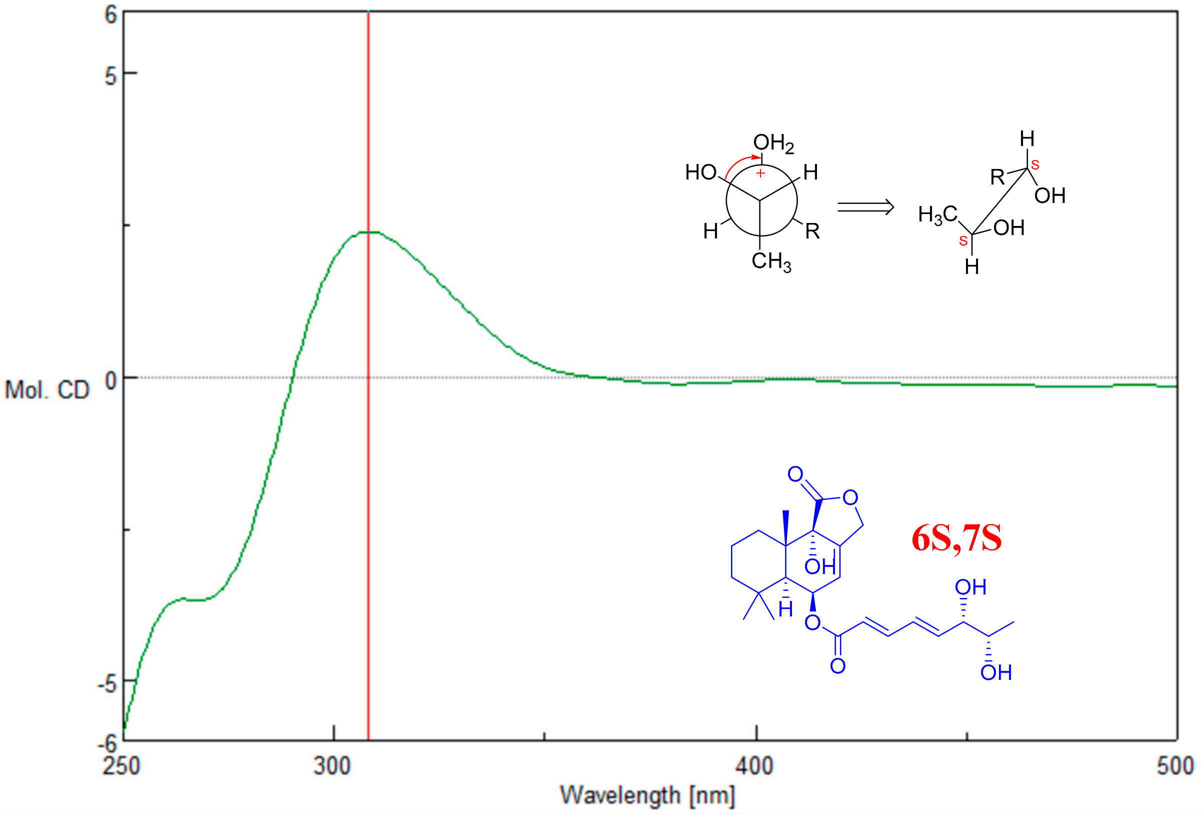


**Figure S75**. The $^{1}$H-NMR spectrum of compound **6** in DMSO-$d_6$

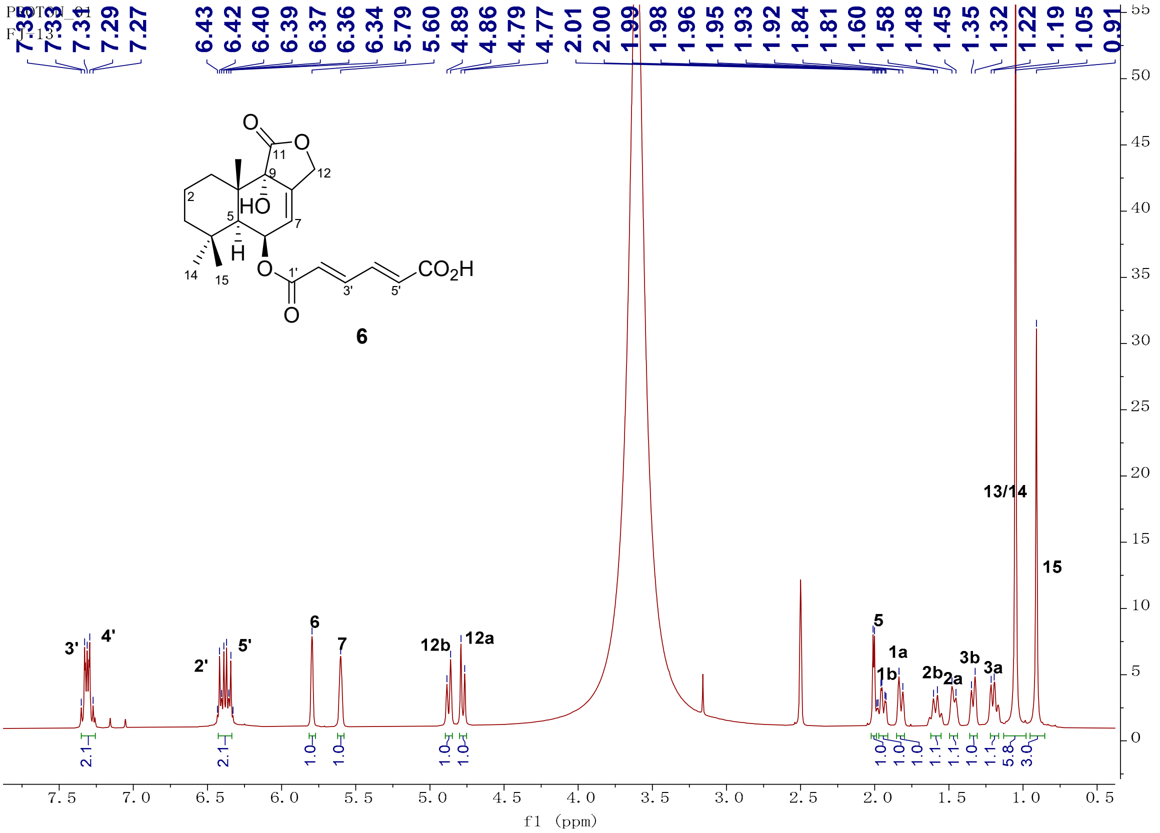

**Figure S76**. The $^{13}$C-NMR spectrum of compound **6** in DMSO-$d_6$

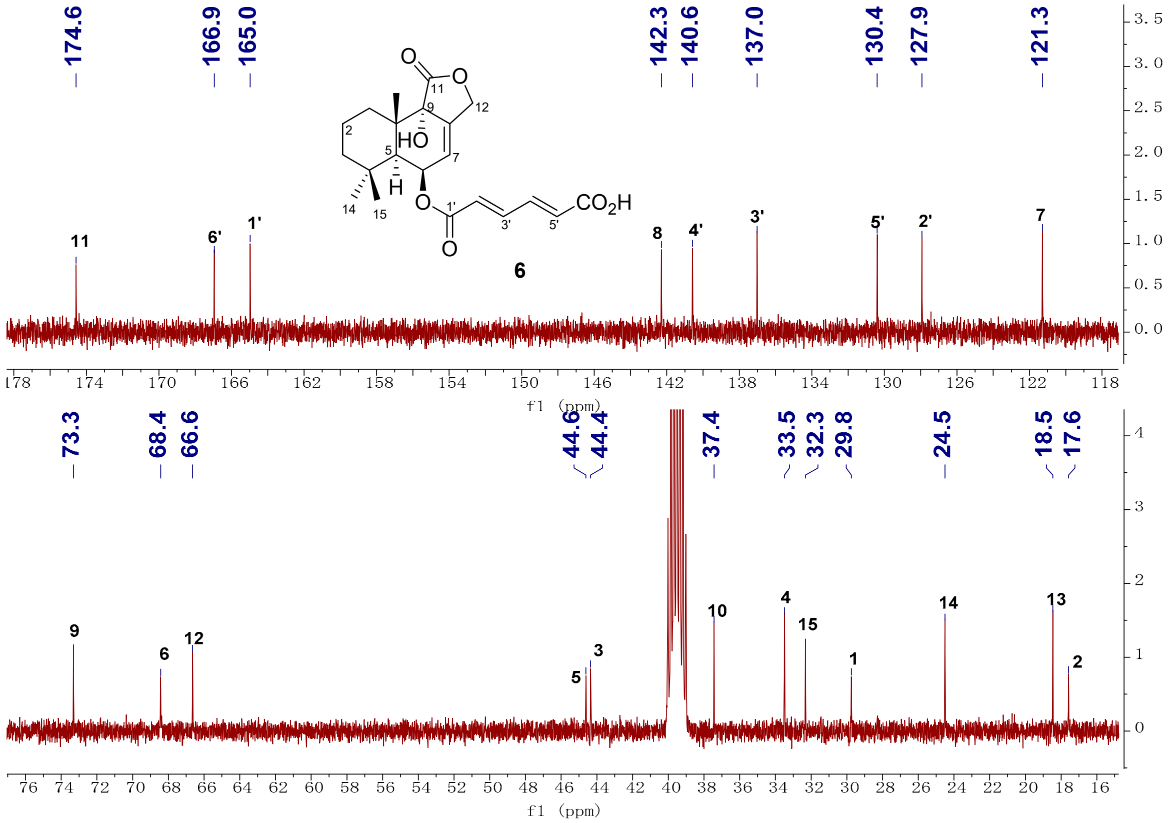


**Figure S77**. The ECD curve of compound **6**

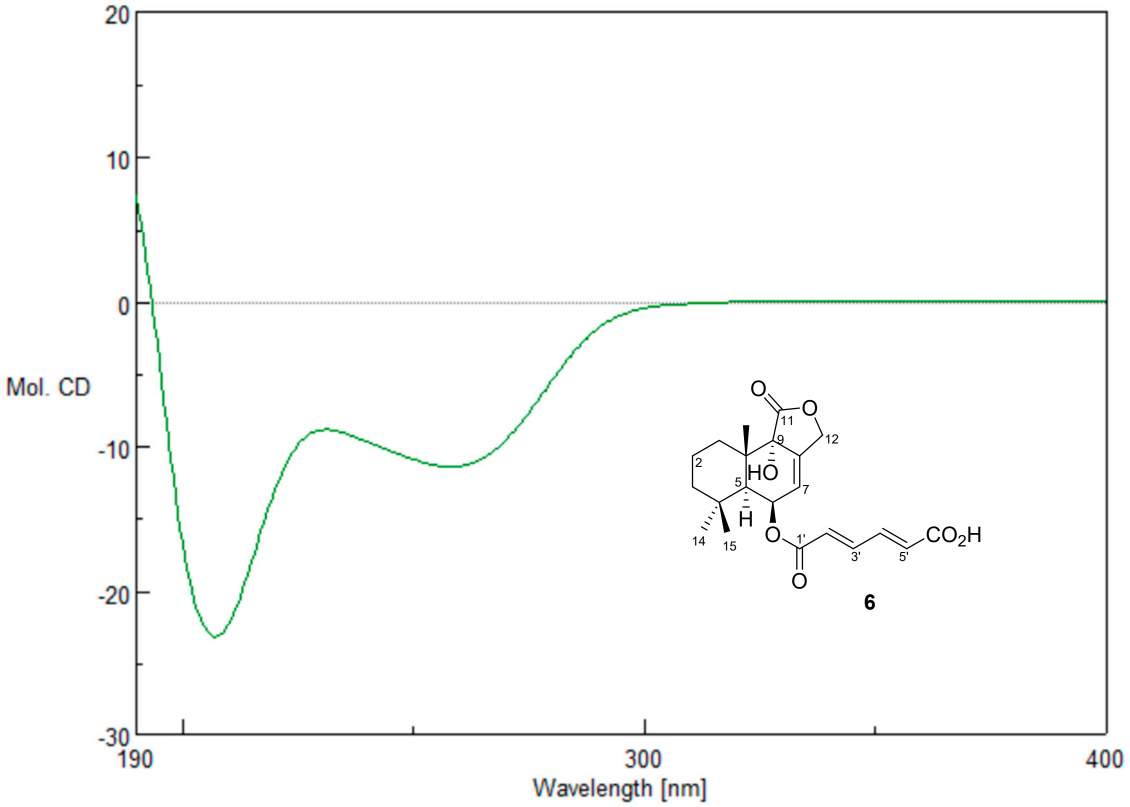

**Figure S78**. The ECD curves of **4**–**6**

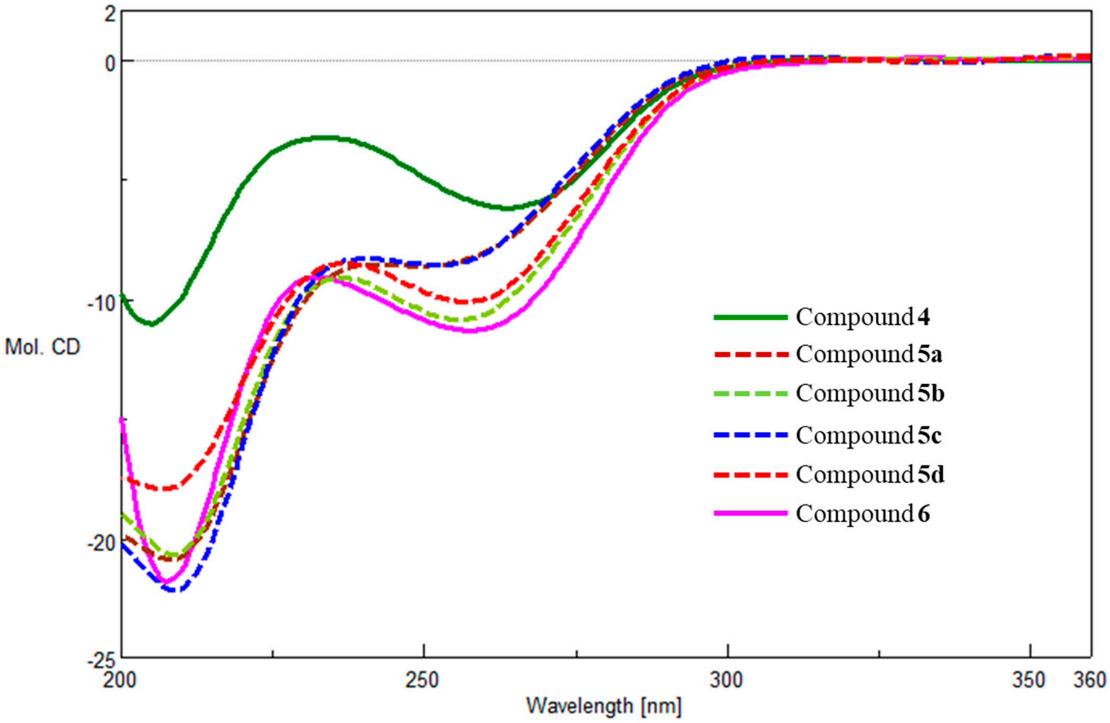


**Figure S79**. The $^{1}$H-NMR spectrum of compound **7** in DMSO-$d_6$

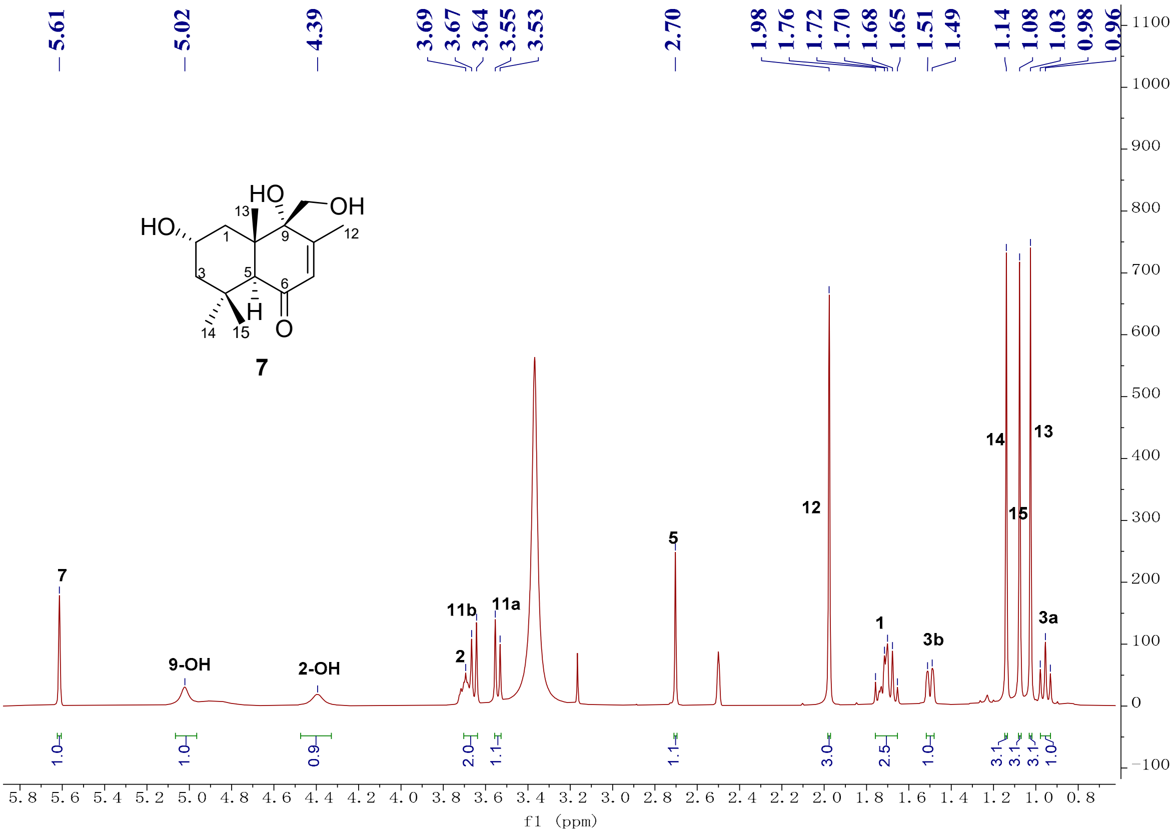

**Figure S80**. The $^{13}$C-NMR spectrum of compound **7** in DMSO-$d_6$

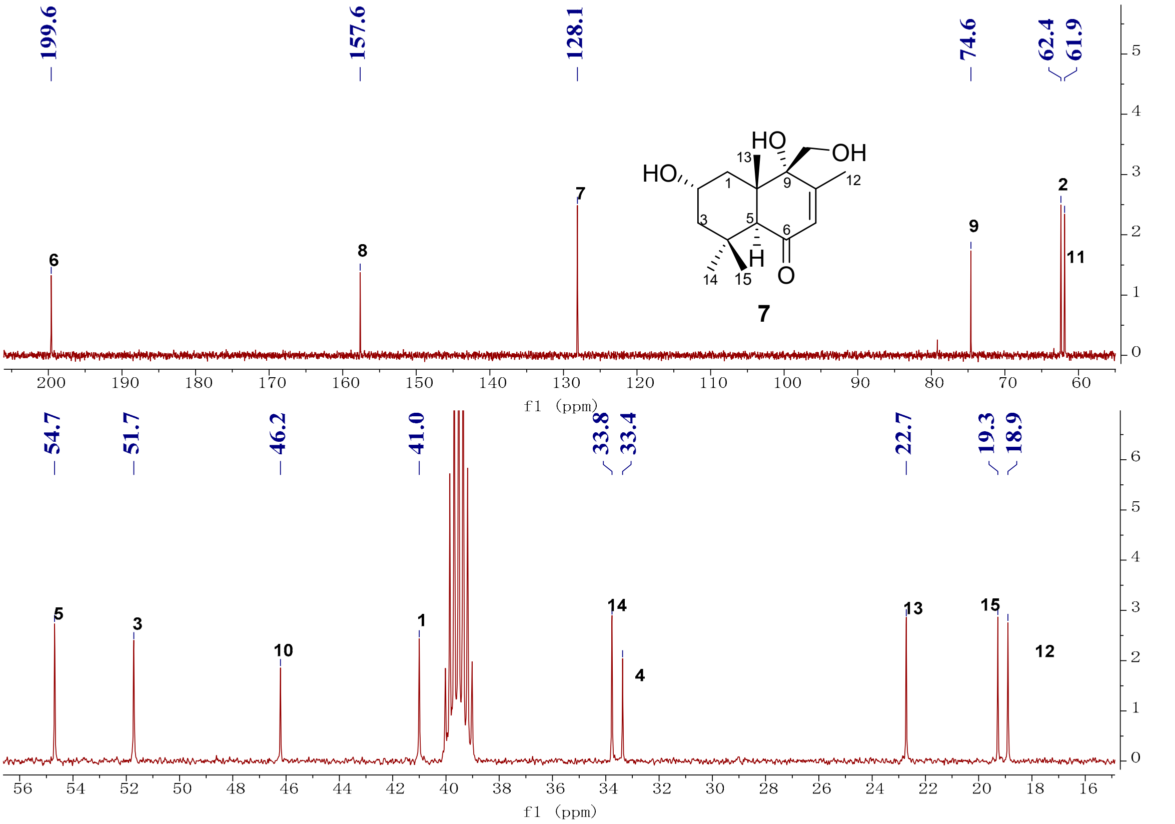


**Figure S81**. The NOESY spectrum of compound **7** in DMSO-$d_6$

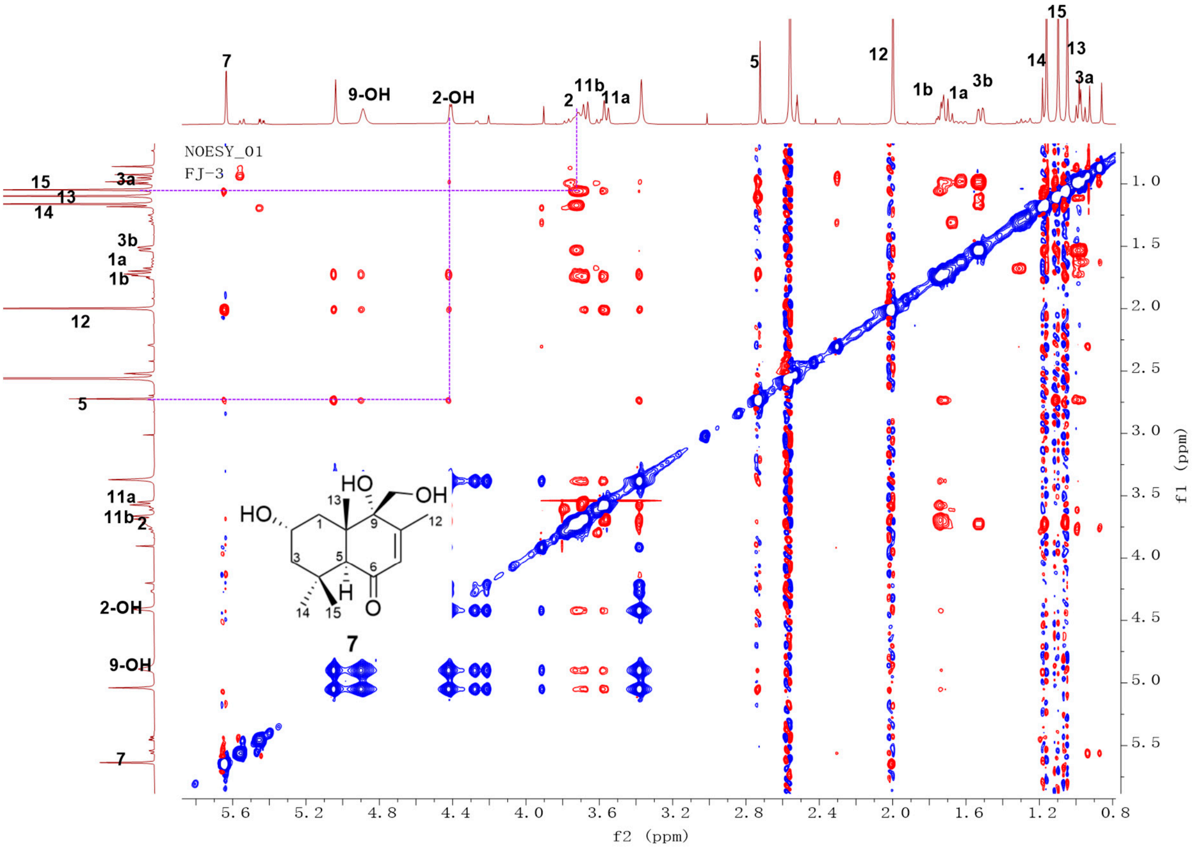

**Figure S81**. The ECD curve of compound **7**

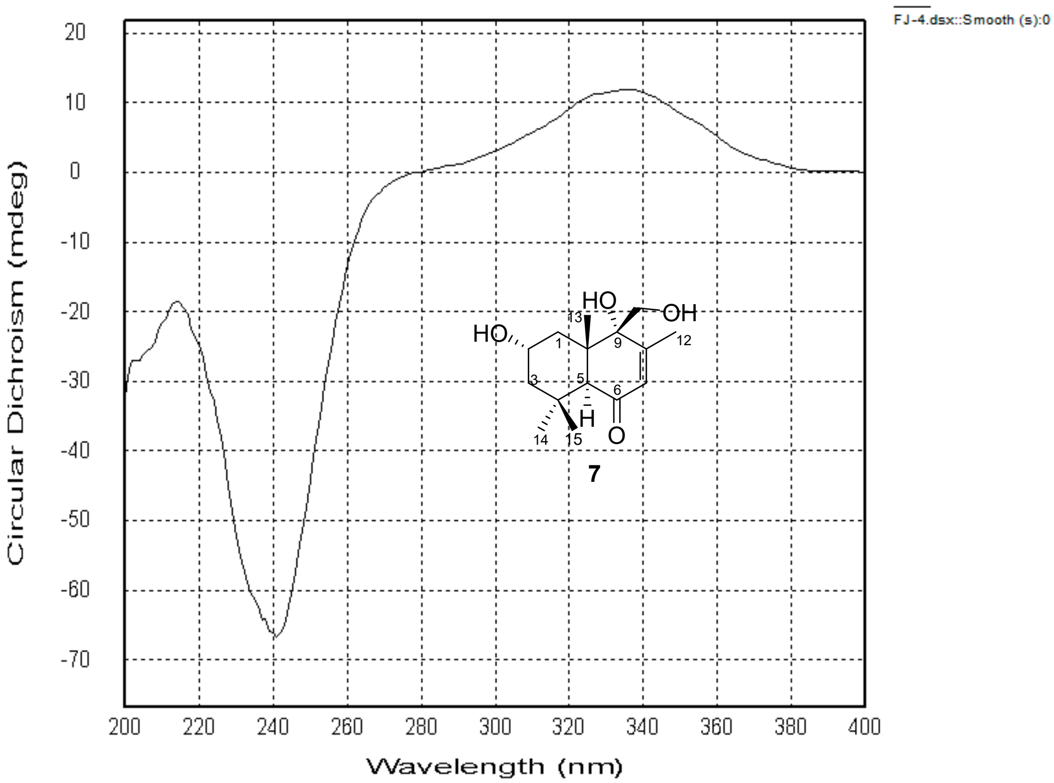


**Figure S83**. The HPLC separation and purification profiles of **5a-5d**
(YMC-pack ODS-A, 50%MeOH-$H_2O$, 3 mL/min)

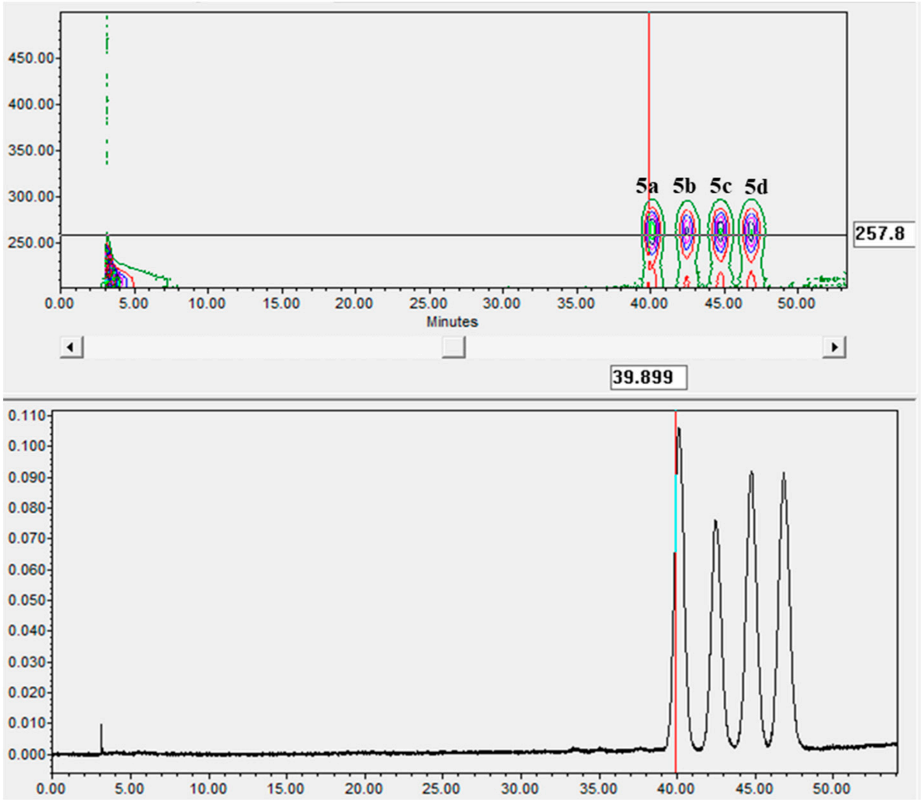

**Figure S84**. The HPLC profile of compound **1**
(YMC-pack ODS-A, 25min 10-100% MeCN-$H_2O$, 1 mL/min)

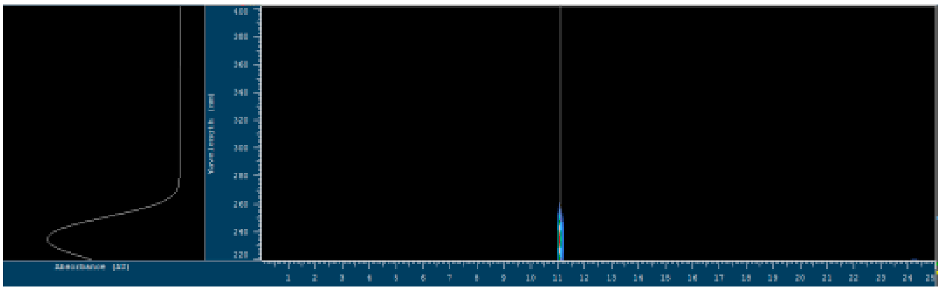

**Figure S85**. The HPLC profile of compound **2**
(YMC-pack ODS-A, 25min 10-100% MeCN-$H_2O$, 1 mL/min)

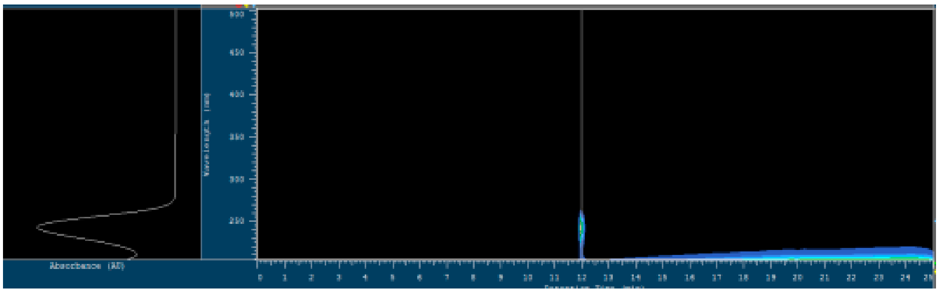

**Figure S86**. The HPLC profile of compound **3**
(YMC-pack ODS-A, 15% MeCN-$H_2O$, 1 mL/min)

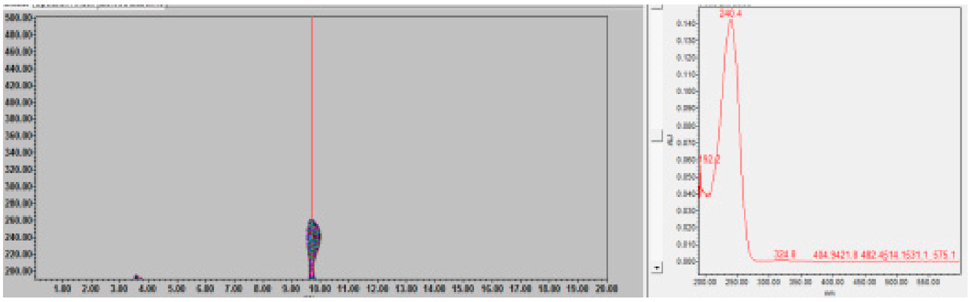

**Figure S87**. The HPLC profile of compound **4**
(YMC-pack ODS-A, 25min 10-100% MeCN-$H_2O$, 1 mL/min)

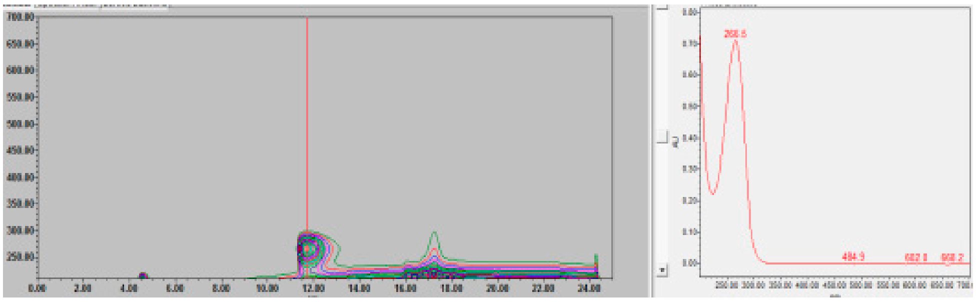

**Figure S88**. The HPLC profile of compound **5** (YMC-pack ODS-A, 35% MeCN-$H_2O$, 1 mL/min)

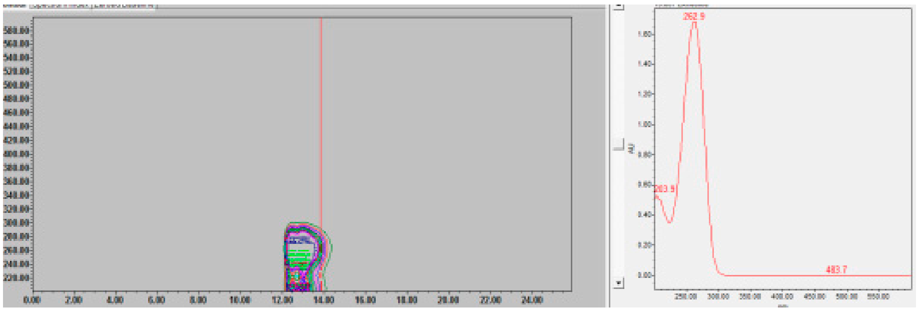

**Figure S89**. The HPLC profile of compound **6** (YMC-pack ODS-A, 25min 10-100% MeCN-$H_2O$, 1 mL/min)

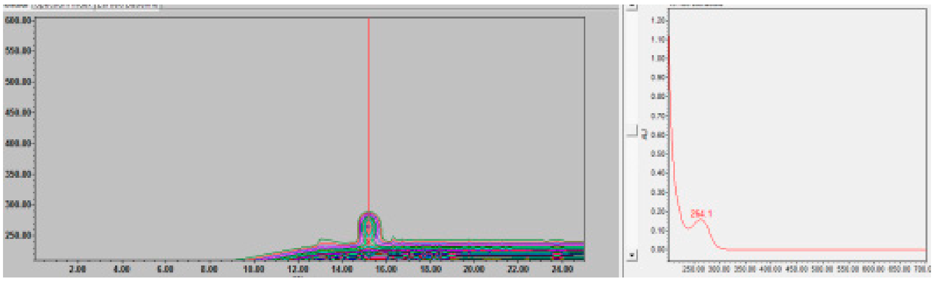

**Figure S90**. The HPLC profile of compound **7** (YMC-pack ODS-A, 20% MeCN-$H_2O$, 1 mL/min)

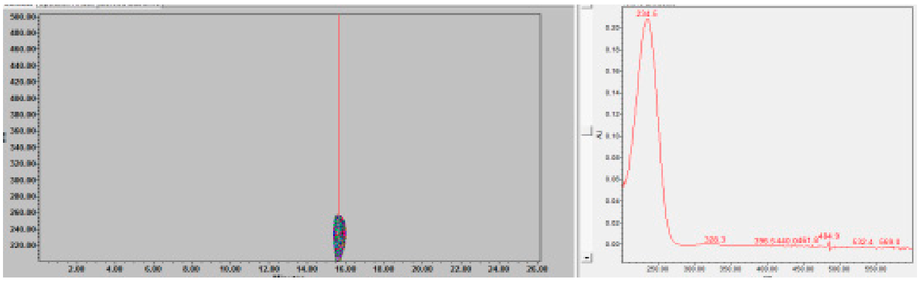

**Figure S91**. The HPLC profile of compound **7** and ustusol B (in ref. 4) and their mixture.
(YMC-pack ODS-A, 25min 10-100% MeCN-$H_2O$, 1 mL/min)

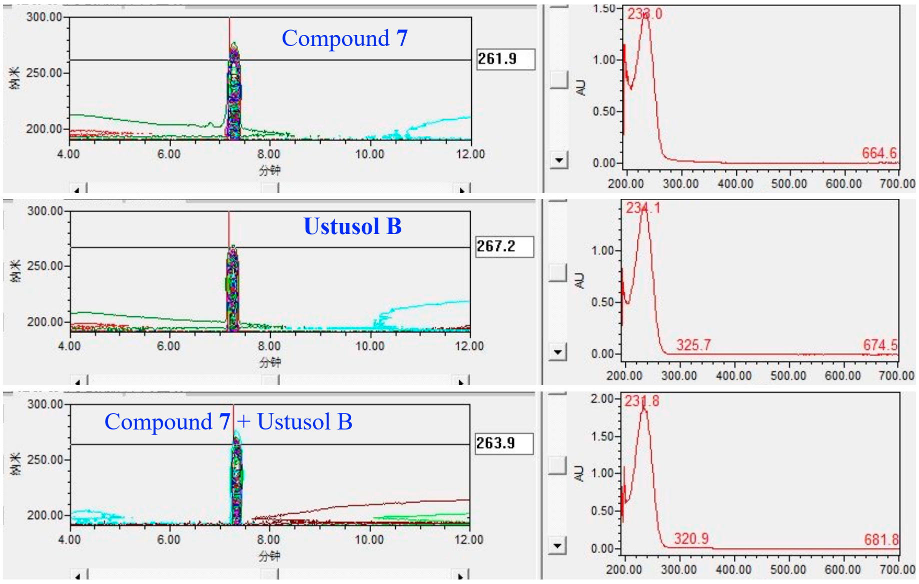

**Table S1**. $^{1}H$ and $^{13}C$ NMR Data for Compound **7** and Ustusol B in DMSO-$d_6$ (TMS, $\delta$ ppm)

| Ustusol B [a] | | | Compound **7** | |
|---|---|---|---|---|
| 1 | 41.0, $CH_2$ | 1.70, m | 41.0, $CH_2$ | 1.71–1.65, m |
| | | | | 1.76–1.71, m |
| 2 | 62.4, CH | 3.71, m | 62.4, CH | 3.68–3.72, m |
| 3 | 51.7, $CH_2$ | 0.96, br.t (12.0) | 51.7, $CH_2$ | 0.96, t (11.9) |
| | | 1.51, m | | 1.50, dd (11.9, 3.8) |
| 4 | 33.4, qC | | 33.4, C | |
| 5 | 54.7, CH | 2.71, s | 54.7, CH | 2.70, s |
| 6 | 199.6, qC | | 199.6, C | |
| 7 | 128.1, CH | 5.62, d (1.1) | 128.1, CH | 5.61, s |
| 8 | 157.6, qC | | 157.6, C | |
| 9 | 74.6, qC | | 74.6, C | |
| 10 | 46.2, qC | | 46.2, C | |
| 11 | 61.9, $CH_2$ | 3.66, dd (11.5, 4.8) | 61.9, $CH_2$ | 3.53, d (11.5) |
| | | 3.55[b], dd (11.5,4.8) | | 3.64, d (11.5) |
| 12 | 19.3, $CH_3$ | 1.98, br s | 19.2, $CH_3$ | 1.98, s |
| 13 | 18.9, $CH_3$ | 1.08, s | 18.9, $CH_3$ | 1.08, s |
| 14 | 33.8, CH | 1.15, s | 33.8, $CH_3$ | 1.14, s |
| 15 | 22.7, $CH_3$ | 1.03, s | 22.7, $CH_3$ | 1.03, s |
| 2-OH | | 4.41, d (5.1) | | 4.39, s |
| 9-OH | | 5.02, s | | 5.02, s |

[a] from *J. Nat. Prod*. 2009, 72, 1761–1767. [b] revised 3.55 from 0.96.

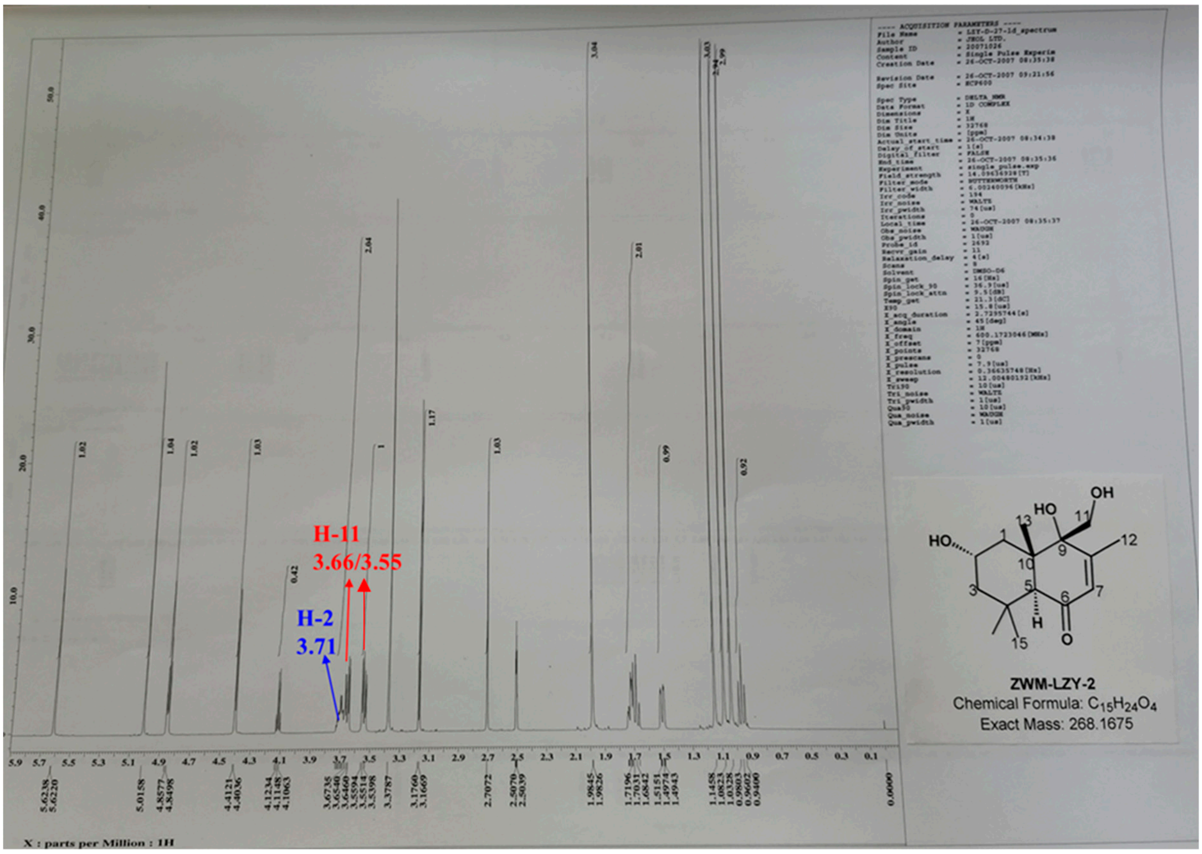


($^{1}H$ (600 MHz) of ustusol B from *J. Nat. Prod*. 2009, 72, 1761–1767)
